# Dispersal patterns in a medium‐density Irish badger population: Implications for understanding the dynamics of tuberculosis transmission

**DOI:** 10.1002/ece3.5753

**Published:** 2019-11-13

**Authors:** Aoibheann Gaughran, Teresa MacWhite, Enda Mullen, Peter Maher, David J. Kelly, Margaret Good, Nicola M. Marples

**Affiliations:** ^1^ Department of Zoology School of Natural Sciences Trinity College Dublin Dublin Ireland; ^2^ Trinity Centre for Biodiversity Research Trinity College Dublin Dublin Ireland; ^3^ Department of Agriculture, Food and the Marine Dublin Ireland; ^4^ Department of Culture, Heritage and the Gaeltacht National Parks and Wildlife Service Dublin Ireland

**Keywords:** badger, dispersal, movement ecology, ranging behavior, tuberculosis

## Abstract

European badgers (*Meles meles*) are group‐living mustelids implicated in the spread of bovine tuberculosis (TB) to cattle and act as a wildlife reservoir for the disease. In badgers, only a minority of individuals disperse from their natal social group. However, dispersal may be extremely important for the spread of TB, as dispersers could act as hubs for disease transmission. We monitored a population of 139 wild badgers over 7 years in a medium‐density population (1.8 individuals/km^2^). GPS tracking collars were applied to 80 different individuals. Of these, we identified 25 dispersers, 14 of which were wearing collars as they dispersed. This allowed us to record the process of dispersal in much greater detail than ever before. We show that dispersal is an extremely complex process, and measurements of straight‐line distance between old and new social groups can severely underestimate how far dispersers travel. Assumptions of straight‐line travel can also underestimate direct and indirect interactions and the potential for disease transmission. For example, one female disperser which eventually settled 1.5 km from her natal territory traveled 308 km and passed through 22 different territories during dispersal. Knowledge of badgers' ranging behavior during dispersal is crucial to understanding the dynamics of TB transmission, and for designing appropriate interventions, such as vaccination.

## Graphical abstract

Only a minority of badgers disperse, and the process has rarely been recorded in detail. However, dispersers could act as important hubs for tuberculosis transmission. Our GPS tracking data reveal the process of dispersal in much greater detail than ever before. They show that measurements of straight‐line distance between old and new social groups severely underestimate how far dispersers travel, the potential for intraspecific interactions and the potential for disease transmission. Understanding the process of dispersal is crucial to understanding disease dynamics and designing appropriate interventions.

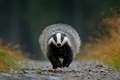

## 1 INTRODUCTION

**Figure 1:**
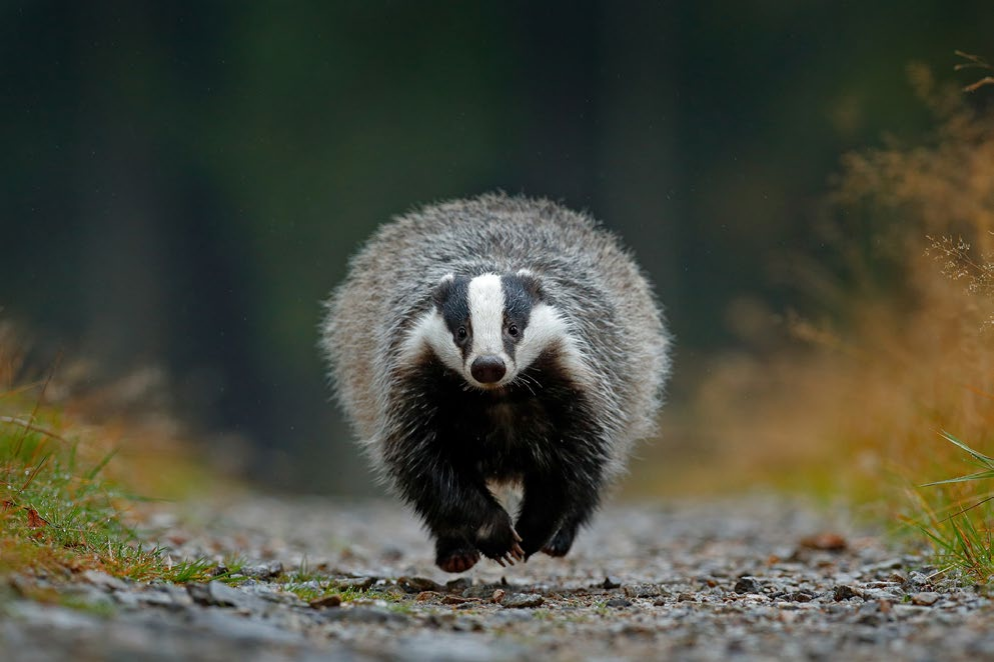
The European badger (*Meles meles*), a group‐living mustelid. Photograph credit: Ondrej Prosicky

Knowledge of ranging behavior and animal movement is particularly important where infectious diseases are difficult to control (Conner & Miller, 2004). European badgers (*Meles meles*, Figure 1) are highly susceptible to *Mycobacterium bovis*, the causative agent of tuberculosis (TB) (Gormley & Costello, 2003). In both Ireland and the UK, badgers have been implicated in the spread of *M. bovis* to cattle and in acting as a wildlife reservoir for bovine TB (Corner, Murphy, & Gormley, 2011; Godfray et al., 2013; Murphy, Gormley, Costello, O'Meara, & Corner, 2010). In order to understand the dynamics of a disease, and to control it successfully, as complete a picture as possible of the movement capabilities of the carrier species is required (Conner & Miller, 2004). The organization of badgers into territorial social groups arguably limits the spread of TB because it lowers disease transmission rates between groups (Cheeseman, Wilesmith, Stuart, & Mallinson, 1988; Davis, Abbasi, Shah, Telfer, & Begon, 2015; Delahay, Langton, Smith, Clifton‐Hadley, & Cheeseman, 2000; Rozins et al., 2018). However, badger movements into and out of neighboring social groups are associated with increased prevalence of TB in these groups (Riordan, Delahay, Cheeseman, Johnson, & Macdonald, 2011; Rogers et al., 1998). Therefore, the movement of badgers is of direct importance to the transmission of TB infection both between individual badgers (O'Mahony, 2015; Weber et al., 2013) and between badgers and cattle (Eves, 1999; Griffin et al., 2005; Martin et al., 1997; Mullen et al., 2015; Woodroffe et al., 2009).

One of the most extensive, but least studied, movements in an animal's life is their dispersal. Dispersal can be defined in different ways—the movement from birth site to breeding site, movement between successive breeding sites and a permanent movement regardless of subsequent reproductive success (Greenwood, 1980). In mammals, dispersal tends to be male‐biased, coupled with female philopatry (Greenwood, 1980). However, in badgers, both sexes are philopatric, and the majority of individuals never permanently leave their natal social group. Indeed, most badger groups are formed by the retention of both sexes (Frantz, Do Linh San, Pope, & Burke, 2010; Woodroffe, Macdonald, & Silva, 1995). However, a minority of badgers do disperse. Therefore, dispersal may be extremely important for the transmission of TB among badgers. By interacting with many other individuals from different social groups, dispersers may act as hubs for disease transmission and contribute disproportionately to disease transmission (Weber et al., 2013).

Although some dispersal does occur in badgers, because it is rarely recorded, little is known about the triggers for and mechanisms underlying dispersal (Byrne, Davenport, O'Keeffe, & Paddy Sleeman, 2012; Roper, 2010). Patterns of dispersal also vary considerably. Contrasting patterns of sex differences have been reported, some studies finding a male bias (Cheeseman, Cresswell, Harris, & Mallinson, 1988; Kruuk & Parish, 1987), others a female bias (Woodroffe et al., 1995), and others found no sex‐bias in the tendency to disperse (Macdonald, Newman, Buesching, & Johnson, 2008). Similarly, there is conflicting evidence regarding a sex difference in the distance dispersed; some studies have found that females disperse further (Christian, 1994; Huck, Frantz, Dawson, Burke, & Roper, 2008; Woodroffe et al., 1995), and others have found that males move further (Pope, Domingo‐Roura, Erven, & Burke, 2006) or that there is no sex‐bias (Cheeseman, Cresswell, et al., 1988). A comparative study of two populations found that the sex‐biases in dispersal tendency and distance moved varied with differing densities and ecologies. Badgers moved further, and dispersal was female‐biased at lower densities compared with no sex‐bias at higher densities (Frantz et al., 2010). In Ireland, a large‐scale study on long‐distance movements using mark–recapture methods found that males moved more frequently, but females moved further away (Byrne et al., 2014). However, it was not possible to distinguish between short‐term extra‐territorial excursions and permanent dispersal events in that study.

To date, most studies on dispersal have been based on radio‐tracking and trapping data which, by their nature, give only a snapshot of badger movement. Most studies include dispersal as part of badger movement in general (Byrne et al., 2014; Cheeseman, Cresswell, et al., 1988; Christian, 1994; Macdonald et al., 2008), and few have focussed solely on dispersal (Roper, Ostler, & Conradt, 2003; Woodroffe et al., 1995). The process of dispersal, as inferred from radio‐tracking five badgers, has been described as lengthy and complex, being characterized by exploratory forays and gradual movement from one group to another (Roper et al., 2003). Here, we present results from a long‐term GPS tracking study (Gaughran, 2018; Gaughran et al., 2018; MacWhite, Maher, Mullen, Marples, & Good, 2013; Mullen et al., 2015) of a medium‐density population in Ireland. This provided an opportunity to examine the behavior of a larger number of dispersing individuals, in some cases over the entire course of their dispersal. This allowed us to elucidate the patterns of dispersal observed in this population. We discuss the possible triggers for dispersal and the implications our findings have for the understanding and management of TB in badgers.

## 2 MATERIALS AND METHODS

The study was conducted in Co. Wicklow, Ireland (52.924130, −6.117960). A description of the study area and details of the trapping and handling of badgers are given in Gaughran et al. (2018). The study area was a matrix of undulating agricultural land (75%), with patches of mixed and coniferous woodland (14%) with small residential areas and farmyards scattered throughout (7%). The population density of 1.8 badgers/km^2^ remained stable over the study period (Gaughran et al., 2018). Badgers were tracked using Tellus Light GPS collars that weighed 240 g (Followit Wildlife, Lindsberg, Sweden). Data collection began in April 2010 and continued until October 2016, when all collars were removed. We aimed to capture as many badgers as possible within each social group. Collars were programmed to record four GPS locations a night, at 21:00, 23:00, 01:00, and 02:00, for each collared badger (following Gaughran et al., 2018; MacWhite et al., 2013). Data were visualized in ArcMap (ArcGIS version 10.4.1).

Badgers were sexed at each trapping event. Age was determined by dentition (Hancox, 1988; da Silva & Macdonald, 1989) and general appearance. Age cohorts were defined as follows: cub (a badger in its first year); yearling (a badger in its second year), young adult (a 2‐ or a 3‐year old), older adult (a 4‐ or a 5‐year old), and aged adult (badger > 5 years old). A social group was defined as the group of badgers that were regularly trapped at the same main sett and whose home ranges overlapped during the time period in question (following (Macdonald et al., 2008; Woodroffe et al., 2016). Thus, badgers were assigned to a social group based on their most frequent trapping location and, if collared, their GPS tracking data.

Dispersers were defined and confirmed retrospectively, as those badgers that were in the process of moving permanently from one social group to another social group. The majority of dispersers were identified using GPS (Figure S1) and/or trapping records. Four dispersers were identified using genetic data (Appendix S1). Where we had GPS records for dispersal events, trajectory maps, which show the sequence of GPS locations, were made using the Point to Line tool in ArcMap (Figure S2.1–S2.13). When a badger was not wearing a collar during the dispersal event itself, maps were made for pre‐ and post‐dispersal periods, or of exploratory forays (Figure S2.14–S2.16). Social group territory boundaries were mapped in ArcMap (Gaughran, 2018), and the centroids were used to estimate the straight‐line distance moved by dispersers. We estimated the number of social groups between the new and old territories by counting how many social groups were intersected by the straight line. If a badger moved to an adjacent social group, this parameter was recorded as zero. Some collared badgers dispersed outside the study area, so the total number of social groups crossed was unknown. Accordingly, the straight‐line distance between the centroid of their original territory and the centroid of the polygon encompassing their GPS locations in their new location was calculated. We divided the straight‐line distance by 1,313 m as this was the mean distance between main setts in the study area (*SD* ± 455 m), to estimate the number of social groups crossed by the badger.

A second analysis was also conducted to describe the trajectory each badger took during dispersal, considering each day's travel rather than just the start and end points of the dispersal event. The creation of trajectories using the GPS locations of badgers allowed us to estimate much more accurately the distances traveled during the process of dispersal. Although these distances are underestimates, as they are derived from the straight‐line distances between only four GPS locations a night, they are less negatively biased than straight‐line distances between social groups. We counted the number of territories crossed by these trajectories. Where trajectories fell outside the known study area, we applied a 1.3 km^2^ grid (based on the mean distance between main setts) to the map and used the grid squares crossed by trajectories as a proxy for estimating the number of territories crossed by the dispersing badgers.

Ethical approval for the project was granted by Trinity College Dublin's Animal Research Ethics Committee (Project No. 290516) and the Health Products Regulatory Authority (Project No. 7024754). Badgers were captured under licences (NPWS Nos. 101/2009, 04/2010, 13/2010, C123/2010, 03/2011, C040/2011, C03/2013, C005/2013, and C001/2015) as required by the Wildlife Act, 1976. Both cage traps and stopped‐restraints conformed to national legislation for humane trapping defined in the Wildlife Act, 1976, Regulations 2003 (S.I. 620 of 2003).

Statistical analysis was carried out in R (version 3.4.0, R Core Team, 2018). Due to a low sample size of individual dispersers (*N* = 25), and because the assumptions of normality and homogeneity of variance were not met, we used Pearson's chi‐square tests (“stats” package) or Fisher's exact tests (“stats” package). To test for a difference between the sexes in their tendency to disperse, all badgers trapped within the study area were categorized as either dispersers or nondispersers, and as male or female. To investigate patterns within dispersers, the data for the number of social groups crossed were amalgamated into a two‐level categorical variable, *destination* (two levels: “next door” and “further away”). The data were tested for interactions between sex and dispersal status, sex and destination, age cohort and destination, and sex and age cohort.

## 3 RESULTS

During the course of the study, 139 badgers were trapped (63 males and 76 females). Of these, 23 resident badgers were identified as dispersers (Table 1). Of the dispersal events identified, 19 were based on GPS and/or trapping records and four inferred from genetic evidence (Table 1, Appendix S1). In addition, two individuals were assumed to be immigrants from outside the study area (Table 1). The immigrants appeared in the study population as adults having never been trapped before. Twelve resident badgers were wearing GPS collars at the time of dispersal/attempted dispersal and one nonresident was collared when trapped while immigrating into the study area. Three more resident badgers wore collars before and after dispersal, but not during the event itself.

**Table 1:** Details of all badgers in the study area that dispersed during the study period, including the two immigrants. Straight‐line estimates were derived from the centroid of old and new social groups. Full trajectory estimates were derived by summing the distance between consecutive GPS locations

Badger	Sex	Year	Age	Straight‐line		Full trajectory	
				Distance (km)	No. SGs crossed	Distance traveled (km)	No. additional SGs visited
F01^a^	F	2014	1	3.097	3	NA	NA
F02^b^	F	2016	5	1.166	0	NA	NA
F03^c^	F	2012	3	0.756	0	6	1
F04^a^	F	2017	2	1.428	0	NA	NA
F05^c^	F	2011	4	1.268	0	NA	NA
F06^c^	F	2013	5	2.06	3	NA	NA
F07^a^	F	NA	NA	1.612	1	NA	NA
F08^d^	F	2016	1	7.609	5	272	16
F09	F	2013	2	4.15	2	75.094	9
F10	F	2015	1	10.544	7	147.62	19
F11	F	2015	2	1.52	1	307.737	22
F12	F	2016	1	2.16	1	66.648	24
M01	M	2011	2	0.777	0	31.222	2
M02^d^	M	2013	2	4.66	3	111.509	6
M03^a^	M	2011	1	1.444	0	NA	NA
M04	M	2013	2	1.468	0	20.43	2
M05^c^	M	2011	3	1.857	0	NA	NA
M06^b^	M	2016	1	1.428	0	NA	NA
M07	M	2015	4	1.243	0	210.776	6
M08^d^	M	2015	2	1.095	0	137.759	3
M09^d^, ^e^	M	2016	OA	NA	NA	110.705	11
M10^c^	M	NA	NA	1.089	0	NA	NA
M11^b^	M	2016	3	1.13	0	NA	NA
M12^e^	M	2015	YA	NA	NA	NA	NA
M13	M	2011	3	0.777	0	59	2

Abbreviations: NA, no GPS data available; OA, Older adult; YA, Younger adult.

a Dispersal inferred from genetic data (Appendix S1).

b Dispersal inferred from trapping data.

c Badgers not wearing GPS collars during dispersal, but collared before and after.

d Exploratory forays only recorded by GPS collar or badger died during the process of dispersal.

e Immigrant, location of natal group unknown.

Of our study population, 16% of females (12/76) and 21% of males, including both immigrants, (13/63) dispersed. There was no sex‐bias in the tendency to disperse (Pearson's chi‐square, *χ* ^2^ = 0.26898, *df* = 1, and *p*‐value = .604). Over a quarter (26%) of badgers dispersed as yearlings (*N* = 6), 57% dispersed as young adults (*N* = 13) while 17% dispersed as older adults (*N* = 4). Dispersal age was unknown for two badgers. Neither cub nor aged adult dispersers were recorded. The median straight‐line distance for dispersal was 1.4 km away from the natal group (range 756 m–10.5 km, mean = 2.4 km, *SD* ± 2.3 km, Table S1). The majority of dispersers moved to adjacent social groups (61%, *N* = 14), (median = 0 social groups, range 0–7 social groups, mean = 1 social group, *SD* ± 1.9 social group, Table S1). Dispersal generally occurred in the first half of the year. Of the badgers that were wearing GPS collars (*N* = 13) at the time of dispersal, or attempted dispersal, 77% (*N* = 10) began the process of dispersal in either January or February. One male, M08, was in the process of dispersal when collared in April but may have begun his dispersal earlier. M09, an immigrant, was collared in August, but it is unknown when he left his natal group. No badger was recorded commencing dispersal in the second half of the year.

### 3.1 Sex and age biases in dispersal

**Figure 2:**
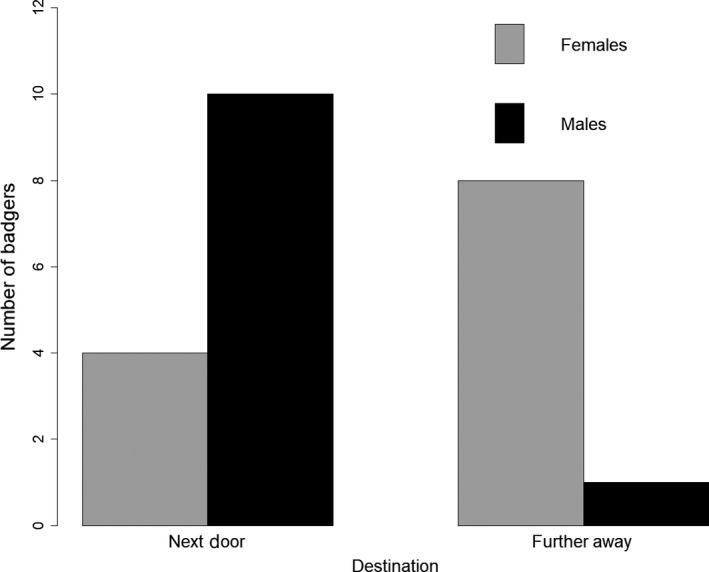
Barplot of dispersal destination in badgers. The number of individuals that moved either next door or further away for female (gray bars) and male (black bars) badgers

More females (67%, *N* = 8) than males (9%, *N* = 1) moved to social groups further away than to adjacent social groups (Fisher's exact test, *p* < .01, Figure 2). Female dispersers were less likely to move next door (33%, *N* = 4) than to move further away (43%, *N* = 8). In contrast, male dispersers were much more likely to move next door 91% (*N* = 10) than to move further away (9%, *N* = 1).

Our data suggest that yearlings (4/6 = 67%) were the most likely to move further away from home and that this tendency decreased with age (young adults 31% and older adults 25%), but there was no statistically significant difference in dispersal distance between age cohorts (Fisher's exact test, two‐sided, *p* = .426).

Of the dispersers, 42% of females and 73% of males were young adults, making this age cohort the most frequent to disperse. Older adults were the least frequent age cohort to disperse (25% of female dispersers and 9% of male dispersers). One‐third of female dispersers and 18% of male dispersers moved as yearlings. While males appeared most likely to disperse as young adults and females appeared likely to disperse at any age, no age‐sex interaction was found (Fisher's exact test, two‐sided, *p* = .318).

### 3.2 The process of dispersal

**Figure 3:**
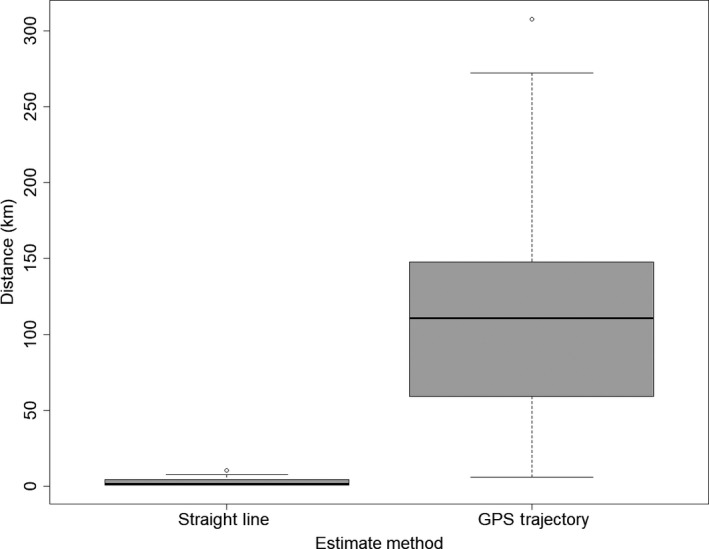
Boxplot of comparing the two methods for estimating dispersal distance (km) in badgers: straight‐line estimate and GPS trajectory estimate

**Figure 4:**
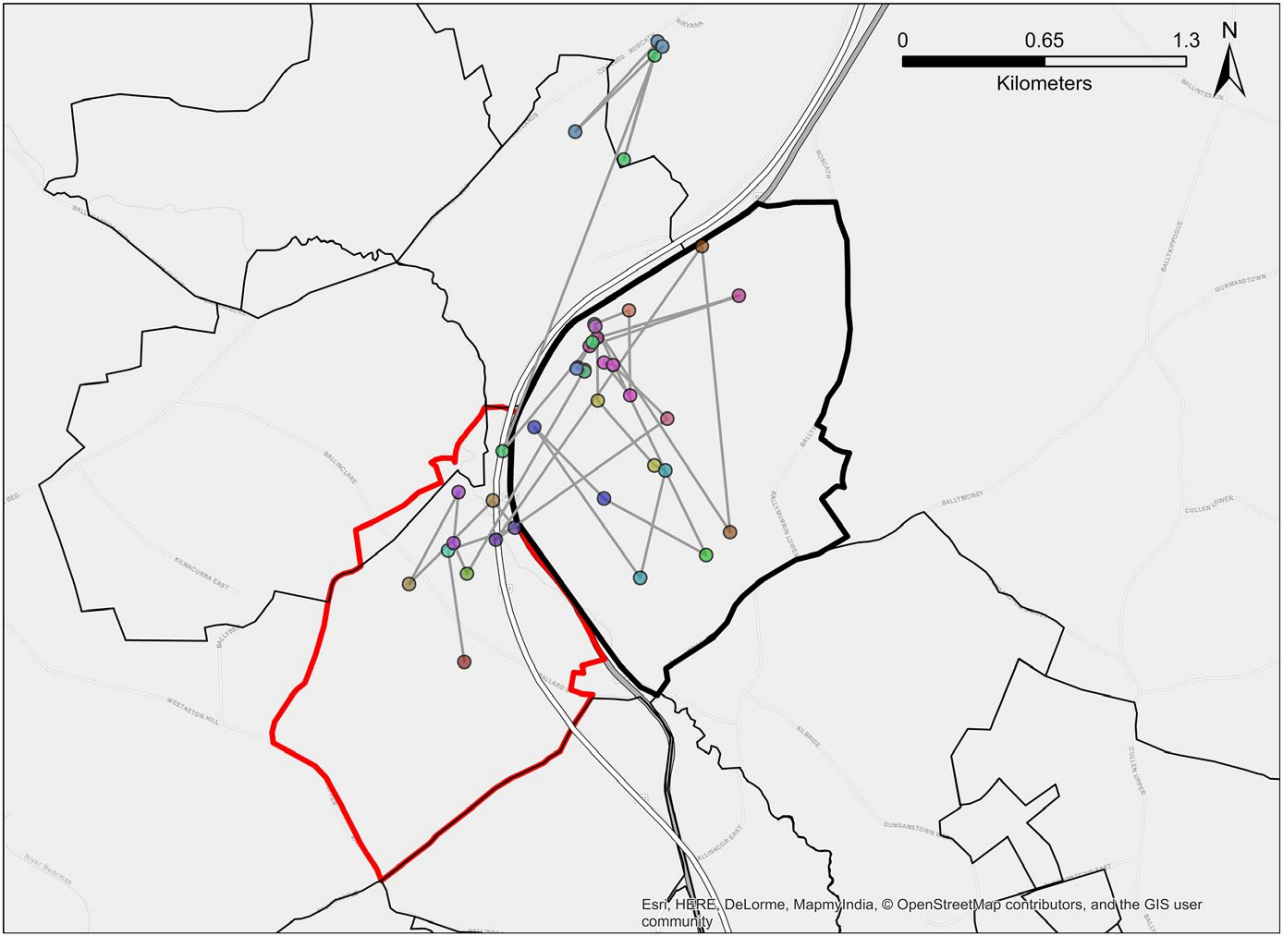
A simple dispersal trajectory. GPS locations for a male, M04, in February 2013. He dispersed overnight on the 10th of February, making only two excursions back to his natal group. During this time, he traveled 20.5 km through an area of approximately 2 km^2^, visiting approximately additional two social groups. Filled circles represent GPS locations, with different colors indicating different nights. Thin gray lines join consecutive GPS locations. The thick red line outlines the natal territory boundary, the thick black line represents the new territory boundary, and thin black lines outline other territory boundaries. The average distance between main setts in the study area was 1.3 km^2^

Where we had full dispersal trajectories (*N* = 13), the mean of the straight‐line distances between natal and new social groups was 3.1 km, whereas the mean of the full trajectory distances was 120 km (Figure 3). Of these 13 individuals, 8 (62%) traveled through more than five additional territories and 7 (54%) traveled distances of more than 100 km. Furthermore, GPS data revealed that the process of dispersal showed a spectrum of complexity and was rarely the same for two badgers. For example, in February 2013, a male badger, M04, dispersed to an adjacent territory overnight. Following dispersal, he made only two excursions back to his natal territory (Figure 4). Extraterritorial excursions were also made to the north of his new territory. During that month, he traveled 20.5 km through an area of approximately 2 km^2^, visiting two other social groups' territories. In contrast, the more complex examples of dispersal typically took place over periods longer than a single night, to either adjacent or further territories.

**Figure 5:**
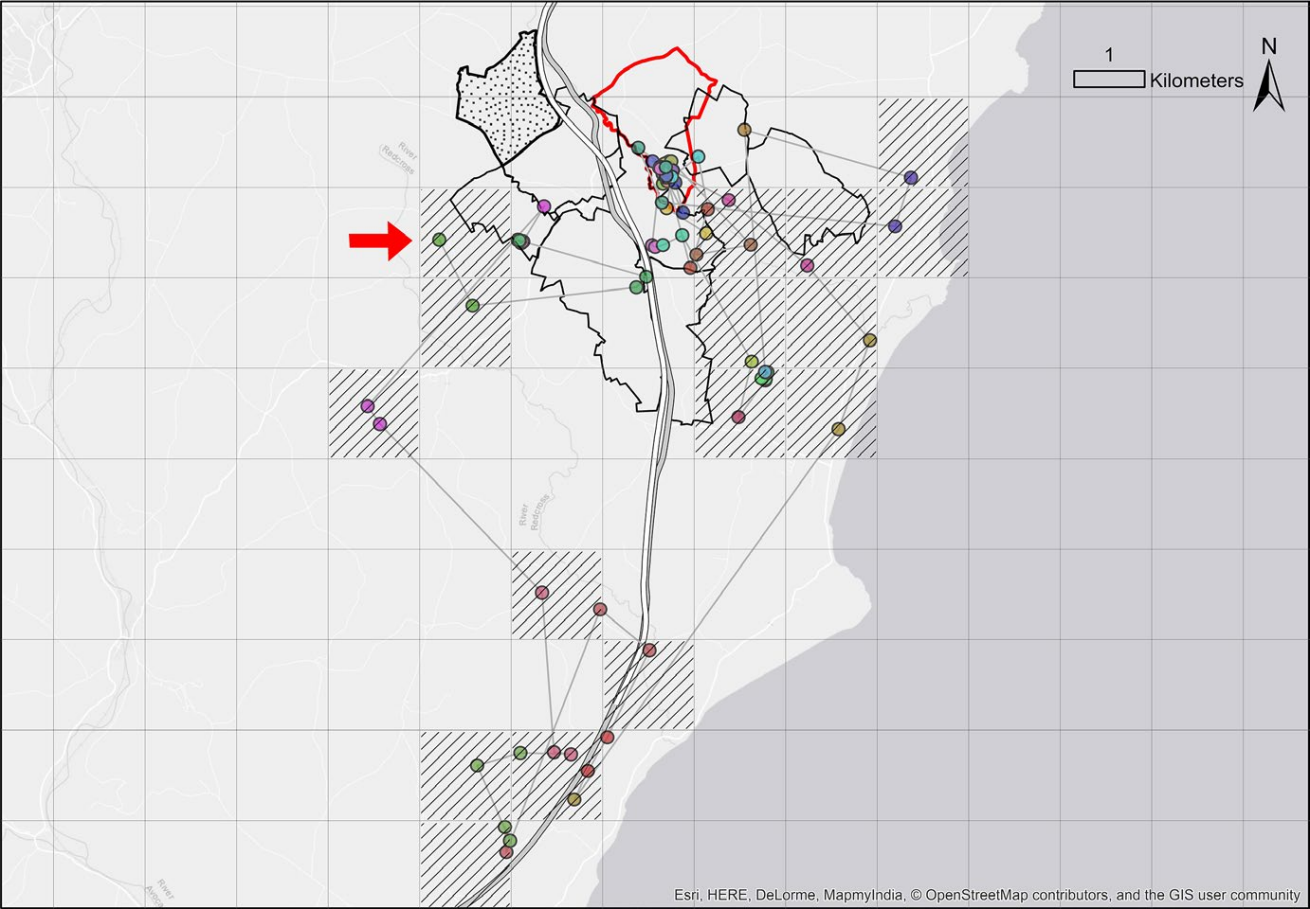
A complex dispersal trajectory. GPS locations for a female, F12, during dispersal in January and February 2016. The trajectory illustrates exploratory forays preceding an eventual move to a nonadjacent social group. ETEs were made over multiple nights where she did not return to the natal social group but spent the day in the territories of other social groups. During this time, she traveled 66 km through an area of 40 km^2^, passing through approximately 22 social groups' territories. However, her GPS collar failed before she completed dispersal, so these figures are underestimates. Filled circles represent GPS locations, with different colors indicating different nights. Thin gray lines join consecutive GPS locations. The thick red line outlines the natal territory boundary, and thin black lines outline other territory boundaries. The stippled area represents the territory she dispersed to. The average distance between main setts in the study area was 1.3 km^2^. A grid of 1.3 km^2^ is used as a proxy for social group territories outside of the study area. The red arrow indicates the last GPS location received before collar failure

In these more complex cases, badgers generally started the dispersal process by making single‐night extraterritorial excursions (ETEs), followed by prolonged ETEs of multiple consecutive nights and gradually shifting the proportion of time spent in each territory (from its natal territory to its new territory), until it finally ceased to return home for example, M08 (Figure S2.11). Many of the dispersal events that resulted in long‐distance movements were characterized by ETEs that spanned two or more nights, for example, F12 (Figure 5), F09, F10, and M02 (Figure S2.3, S2.4, S2.8). For example, one female, F08, covered 17 km in an ETE that spanned two nights (Figure S2.2). The maximum straight‐line dispersal distance was recorded by F10, a female who moved 10.5 km away from her natal group. That straight line crossed the territories of 7 other social groups. However, analysis of her trajectory showed that during dispersal, F10 traveled 147 km through an estimated 19 different social groups. At the most extreme end of the dispersal, spectrum was F11, which moved to a territory only 1.5 km away from her natal territory (Figure S2.5). However, before moving into her new territory she ranged widely for 6 months, traveling 307 km through an area of approximately 25 km^2^. During this period, she passed through approximately 22 other social groups' territories. Even badgers that moved to adjacent territories were seen to cover considerable distances and visit several different social groups, for example, M07 (Figure S2.10) and M08 (Figure S2.11).

## 4 DISCUSSION

### 4.1 Dispersal rate and distance

Approximately 18% of the badgers trapped were shown to have dispersed over the 7 years of the study. This is similar to rates published for other populations of both higher and lower densities (Dugdale, Macdonald, Pope, & Burke, 2007; Macdonald et al., 2008; Revilla & Palomares, 2002; Woodroffe et al., 1995). Our calculation is likely to be an underestimate as we could not know which badgers, first trapped as adults, had already dispersed when the study began. In addition, some badgers “disappeared” from the study area, and we could not determine whether we had failed to retrap these individuals, or they had died, or whether they had dispersed beyond the study area undetected.

Population density varies widely across the European badger's range (Gaughran, 2018; Johnson & Jetz, 2002). While dispersal rates appear to be similar across populations, dispersal distance may be inversely related to population density (Frantz et al., 2010). Studies of some high‐density populations have found that the majority of, or in some cases all, dispersers moved to adjacent social groups (Kruuk & Parish, 1987, Roper et al., 2003, Macdonald et al., 2008, but see Woodroffe et al., 1995 and Rogers et al., 1998). In our study, where the population is of intermediate density (1.8 badgers/km^2^), the majority of dispersing badgers (61%) moved to adjacent social groups, but a large proportion (39%) moved to social groups that were further away. One individual (F10) moved 10.5 km away (straight‐line distance) from her natal group. A large‐scale trapping study in Ireland by Byrne et al. (2014) concluded that long‐distance movements of badgers have been greatly underestimated, reporting one badger recaptured 22.1 km from its original capture site. In a low‐density Spanish population, a dispersal distance of 32 km has been reported (Revilla & Palomares, 2002). Our findings support the finding of Frantz et al. (2010) that badgers are likely to disperse further in lower density populations.

### 4.2 Dispersal age and sex

The majority of badgers dispersed as 2‐ or 3‐year‐olds, agreeing with many studies (Cheeseman, Cresswell, et al., 1988; Macdonald et al., 2008; Revilla & Palomares, 2002; Woodroffe et al., 1995). We found that several dispersers (26%) left their natal group as yearlings, similar to Rogers et al. (1998). We did not record any badgers dispersing as cubs (*c.f.* Christian, 1994). We found no evidence for sex‐bias in the tendency of badgers to disperse, which agrees with some studies of high‐density British populations (Macdonald et al., 2008; Frantz et al., 2010, but *c.f.* Christian, 1994: female‐bias). Frantz et al. (2010) suggested that sex‐bias in dispersal tendency varies with population density. They found that dispersal was female‐biased in a Swiss population, where densities were similar to our study population. Although we found no difference in the tendency for the sexes to disperse, we did find a sex‐bias in the distance that badgers dispersed. While males most commonly moved to a neighboring territory, females tended to move further. These findings agree with Christian (1994), Woodroffe et al. (1995, but cf. Macdonald et al., 2008), and Huck et al. (2008) and are similar to patterns seen in another group‐living mammal, the beaver *Castor canadensis* (Sun, Müller‐Schwarze, & Schulte, 2000).

### 4.3 Triggers of dispersal

Little is known about what triggers dispersal in badgers (Roper, 2010). Where we are aware of timing, all badgers dispersed during the first 6 months of the year. The majority of collared badgers (71%) commenced dispersal in January or February, similar to a Spanish population (Revilla & Palomares, 2002). When badgers were not wearing GPS collars at the time of dispersal (*N* = 12), we cannot exclude the possibility that they commenced dispersal later in the year. Nonetheless, our data suggest that dispersal is triggered by events that occur early in the year. It is possible that the onset of estrus in January and February, including yearlings coming into season for the first time (Corner, Stuart, Kelly, & Marples, 2015; Roper, 2010), causes these animals to be expelled from their natal sett. Breeding suppression by dominant females has been suggested as a dispersal trigger in groups where there is more than one sexually mature female (Woodroffe & Macdonald, 1995). However, Macdonald et al. (2008) found that dispersal did not affect the likelihood of reproduction in females. Indeed, in our study area, in most years (excluding 2011, when no plural breeding was recorded) between 40% and 67% of captured females that were lactating were plural breeders within their social groups (Table B in Appendix S1). This suggests that breeding suppression is not a major feature of this population. Neither would breeding suppression among females explain the timing of dispersal of male badgers. Perhaps the arrival of new cubs triggers older siblings of both sexes to disperse. This behavior has been recorded in some other mammals (Misaki feral horse, *Equus caballus*, Kaseda, Ogawa, & Khalil, 1997; Eurasian lynx, *Lynx lynx*, Zimmermann, Breitenmoser‐Würsten, & Breitenmoser, 2005).

A change in the location of social group boundaries may trigger dispersal. An “altered‐boundaries” scenario is a possible trigger in this study for two badgers from different social groups, F05 (Figure S2.14) and M11 (Figure S2.16). These badgers were wearing collars before and after their dispersal events, but not during dispersal. The location of their social groups' boundaries changed due to fission in the intervening period. It may be that disruption of territory boundaries through fission or fusion (da Silva, Woodroffe, & Macdonald, 1993; Revilla & Palomares, 2002; Van Apeldoorn, Vink, & Matyáštík, 2006) is a seasonally independent trigger of dispersal.

The triggers of dispersal are likely to be many, but it appears that the tendency for badgers to disperse early in the year, evidenced here, may be related to the reproductive cycle of the badger. However, other events may act as catalysts for dispersal at any time of the year. Perhaps the second half of the year, when badgers are gaining weight in preparation for winter lethargy (Fowler & Racey, 1988), is not an ideal time to disperse from an energy conservation perspective. It is clear that further longitudinal studies, across similarly long timeframes, will be required to elucidate the ecological and social drivers triggering dispersal.

### 4.4 Inbreeding avoidance and dispersal

The sex‐bias in dispersal distance suggests an inbreeding avoidance strategy among female badgers. Inbreeding has been found to intensify the correlation between the aging and the progression of TB infection in female, but not male, badgers (Benton et al., 2018). Extragroup mating, particularly between neighboring social groups, results in spatial clustering of relatives within a badger population (Dugdale, Macdonald, & Pope, 2008). In our population, rates of extragroup paternity were high (70%) and the majority of extragroup matings were of resident females with males from neighboring groups (Appendix S1). If a female moves further away from her natal group, she may reduce the probability of breeding with closely related males (Greenwood, 1980).

### 4.5 The process of dispersal

Our data show that dispersal is a complex process and dispersers do not follow straight lines. We found trajectory calculations for both distance traveled and number of social groups encountered were an order of magnitude greater than those of straight‐line estimates. Straight‐line distances traveled by dispersers were, on average, nearly 40 times smaller than trajectory distances. An assumption of straight‐line movement provided estimates of the number of social groups encountered, on average, 12 times lower than trajectory calculations. Similar severe underestimates have been reported for dispersing red foxes (*Vulpes vulpes*) (Walton, Samelius, Odden, & Willebrand, 2018). It is clear that assumptions of straight‐line travel have serious implications for the calculation of direct and indirect interactions within badger and fox populations across rural landscapes.

Badgers of both sexes were recorded sleeping outside their own territories during dispersal. That badgers spent consecutive nights away from their home setts introduces the possibility that they spent the day in setts belonging to other social groups (e.g., Figure S2.4–S2.6, S2.8, S2.11). It is also possible that dispersers slept elsewhere, as badgers have been observed sleeping in daybeds located in brambles, ferns, or rushes, primarily during the warmer months (E. Mullen, M. Good pers. comm.; Roper, 2010).

### 4.6 Implications for disease transmission

Movement between social groups increases the potential for disease transmission (Böhm, Hutchings, & White, 2009; Riordan et al., 2011; Rogers et al., 1998; Silk et al., 2018, Vicente et al. 2007). Our results suggest that there was a lot of interaction, either direct or indirect, between social groups, as evidenced by the movements of dispersers, which regularly crossed several social groups' territories. Although undertaken by a minority of individuals (18%), dispersal is likely to be important for disease transmission. Our results illustrate that a single individual disperser may interact with over 20 social groups, often sleeping in their territory, providing abundant opportunities for disease transmission. Individuals such as this, who have extensive contact with other individuals, will affect the way in which disease is transmitted through a population, and particularly, a population structured into social groups (Böhm, Palphramand, Newton‐Cross, Hutchings, & White, 2008). In addition, dispersers are more likely to catch diseases such as TB due to their increased contact with animals outside their social group, that is, act as “super‐contractors” (Böhm et al., 2009; McDonald, Robertson, & Silk, 2018). As a consequence, they are likely to make a disproportionate contribution to the spread of disease among social groups (Weber et al., 2013). These individuals may be considered “super‐movers” or “super‐susceptibles” (Craft, 2015), an extension of the concept of disease “super‐spreaders” (Böhm et al., 2009; Lloyd‐Smith, Schreiber, Kopp, & Getz, 2005; McDonald et al., 2018).

Behaviors such as sett sharing, allogrooming, and particularly fighting, all present opportunities for direct transmission of TB (Corner et al., 2011). The two most frequent modes of TB transmission among badgers are respiratory transfer and bite wounds contaminated with infectious saliva (Corner et al., 2011). Infected bite wounds are sources of extremely high numbers of *M. bovis* bacilli (Delahay, Cheeseman, & Clifton‐Hadley, 2001), and this route bypasses the body's natural defences against TB (Corner et al., 2011; Gallagher and Clifton‐Hadley, 2000). Furthermore, it is likely that dispersers follow badger paths and use existing latrines, increasing opportunities for indirect TB transmission via feces, urine, and exudates (Hutchings & Harris, 1997; Scantlebury, Hutchings, Allcroft, & Harris, 2004).

Dispersal is costly (Bonte et al., 2012). The costs include energy and time, the risk of mortality from road traffic accidents or wounding by conspecifics, and trade‐offs between movement and immune‐defence and disease resistance (Bonte et al., 2012). Stressors, for example, territorial pressures, may trigger disease progression in badgers (Gallagher and Clifton‐Hadley, 2000), and stressed individuals are more likely to shed *M. bovis* bacilli than unstressed individuals (Macdonald, Riordan, & Mathews, 2006; Riordan et al., 2011). It is reasonable to assume that dispersal is a physiologically stressful process and poses a disease transmission risk. For example, F11, when recaptured during the course of her protracted dispersal, was not in good physical condition and had many scars on her rump and shoulders from bite wounds.

### 4.7 Opportunities for designing disease control strategies

Understanding which individuals may contribute disproportionally to disease spread is helpful when designing interventions to target particular cohorts or seasons (Silk et al., 2017). Although they comprise a small proportion of badger populations, dispersers should be the focus of targeted vaccination against TB, since their behavior makes them high‐risk individuals in terms of disease transmission. If it were possible to focus vaccination efforts on dispersers, it would make TB control strategies much more efficient and effective. Unfortunately, we have discovered no way of identifying these individuals before they commence dispersal. Therefore, our recommendation is to target vaccination toward cubs (badgers in their first year of life), before they have the opportunity to disperse independently. Our data show a large minority of the badgers which dispersed, did so as yearlings, and that all female yearlings dispersed over long distances. Furthermore, most commenced dispersal in January or February. Therefore, vaccinating younger badgers before they begin to attempt dispersal is crucial. The capture of cubs requires the use of cage traps rather than stopped‐restraints, which fail to capture all but the heaviest cubs (Murphy, O'Keeffe, Martin, Gormley, & Corner, 2009). We have found that trapping these young badgers is most successful in autumn.

## 5 CONCLUSION

Our long‐term analysis of GPS data demonstrates that dispersing badgers range over much larger areas than previously appreciated, coming into contact with an unexpectedly large number of social groups. Due to the relatively low number of GPS locations collected in a night, our estimates of distance traveled are likely to be underestimates (also see Byrne et al., 2014). Nonetheless, knowledge of ranging behaviors, including dispersal, is crucial in understanding the dynamics of TB transmission among badgers. Our findings can improve epidemiological modeling by allowing a better calculation of the transmission potential of a disease, and the vaccination coverage necessary to control the disease. It will allow improvement of the design of vaccination programs by clarifying which cohorts to target, when to target them and the scale at which interventions should take place. This longitudinal study has allowed us to describe the process of dispersal in unprecedented detail, giving us insights into one of the least understood aspects of badger movement ecology.

## CONFLICT OF INTEREST

None declared.

## AUTHOR CONTRIBUTIONS

EM, TMW, PM, and MG initially conceived the project; EM, TMW, PM, DK, MG, and NM developed the ideas and designed methodology; EM, TMW, PM, and AG collected the data; AG, DK, and NM analyzed the data; and AG led the writing of the manuscript in association with DK and NM. All authors contributed critically to the drafts and gave final approval for publication.

## Data Availability

Data are available at https://doi.org/10.5061/dryad.jwstqjq4k.

## References

[ece35753-bib-0001] Benton, C. H. , Delahay, R. J. , Smith, F. A. P. , Robertson, A. , McDonald, R. A. , Young, A. J. , … Hodgson, D. (2018). Inbreeding intensifies sex‐and age‐dependent disease in a wild mammal. Journal of Animal Ecology, 87(6), 1500–1511. 10.1111/1365-2656.12878 29938787

[ece35753-bib-0002] Böhm, M. , Hutchings, M. R. , & White, P. C. (2009). Contact networks in a wildlife–livestock host community: Identifying high‐risk individuals in the transmission of bovine TB among badgers and cattle. PLoS ONE, 4(4), e5016 10.1371/journal.pone.0005016 19401755 PMC2660423

[ece35753-bib-0003] Böhm, M. , Palphramand, K. L. , Newton‐Cross, G. , Hutchings, M. R. , & White, P. C. (2008). Dynamic interactions among badgers: Implications for sociality and disease transmission. The Journal of Animal Ecology, 77(4), 735–745. 10.1111/j.1365-2656.2008.01377.x 18355241

[ece35753-bib-0004] Bonte, D. , Van Dyck, H. , Bullock, J. M. , Coulon, A. , Delgado, M. , Gibbs, M. , … Travis, J. M. J. (2012). Costs of dispersal. Biological Reviews, 87(2), 290–312. 10.1111/j.1469-185X.2011.00201.x 21929715

[ece35753-bib-0005] Byrne, A. W. , Davenport, J. , O'Keeffe, J. , & Paddy Sleeman, D. (2012). The ecology of the European badger (*Meles meles*) in Ireland: A review. Biology & Environment: Proceedings of the Royal Irish Academy, 112(1), 105–132. 10.3318/bioe.2012.02

[ece35753-bib-0006] Byrne, A. W. , Quinn, J. L. , O'Keeffe, J. J. , Green, S. , Paddy Sleeman, D. , Wayne Martin, S. , & Davenport, J. (2014). Large‐scale movements in European badgers: Has the tail of the movement kernel been underestimated? The Journal of Animal Ecology, 83(4), 991–1001. 10.1111/1365-2656.12197 24410133

[ece35753-bib-0007] Cheeseman, C. L. , Cresswell, W. J. , Harris, S. , & Mallinson, P. J. (1988). Comparison of dispersal and other movements in two badger (*Meles meles*) populations. Mammal Review, 18(1), 51–59. 10.1111/j.1365-2907.1988.tb00071.x

[ece35753-bib-0008] Cheeseman, C. L. , Wilesmith, J. W. , Stuart, F. A. , & Mallinson, P. J. (1988). Dynamics of tuberculosis in a naturally infected badger population. Mammal Review, 18(1), 61–72. 10.1111/j.1365-2907.1988.tb00073.x

[ece35753-bib-0009] Christian, S. (1994). Dispersal and other inter‐group movements in badgers, *Meles meles* . Zeitschrift Für Säugetierkunde, 59(4), 218–223.

[ece35753-bib-0010] Conner, M. M. , & Miller, M. W. (2004). Movement patterns and spatial epidemiology of a prion disease in mule deer population units. Ecological Applications, 14(6), 1870–1881. 10.1890/03-5309

[ece35753-bib-0011] Core Team, R. (2018). R: A language and environment for statistical computing. Vienna, Austria: R Foundation for Statistical Computing Retrieved from https://www.R-project.org/

[ece35753-bib-0012] Corner, L. A. , Murphy, D. , & Gormley, E. (2011). *Mycobacterium bovis* infection in the Eurasian badger (*Meles meles*): The disease, pathogenesis, epidemiology and control. Journal of Comparative Pathology, 144(1), 1–24. 10.1016/j.jcpa.2010.10.003 21131004

[ece35753-bib-0013] Corner, L. A. , Stuart, L. J. , Kelly, D. J. , & Marples, N. M. (2015). Reproductive biology including evidence for superfetation in the European badger *Meles meles* (Carnivora: Mustelidae). PLoS ONE, 10(10), e0138093 10.1371/journal.pone.0138093 26465324 PMC4605486

[ece35753-bib-0014] Craft, M. E. (2015). Infectious disease transmission and contact networks in wildlife and livestock. Philosophical Transactions of the Royal Society B: Biological Sciences, 370(1669), 20140107 10.1098/rstb.2014.0107 PMC4410373 25870393

[ece35753-bib-0015] da Silva, J. , & Macdonald, D. W. (1989). Limitations to the use of tooth wear as a means of ageing Eurasian badgers, *Meles meles* . Revue D'ecologie (La Terre Et La Vie), 44, 275–278.

[ece35753-bib-0016] da Silva, J. , Woodroffe, R. , & Macdonald, D. W. (1993). Habitat, food availability and group territoriality in the European badger. Meles meles. Oecologia, 95, 558 10.1007/BF00317441 28313297

[ece35753-bib-0017] Davis, S. , Abbasi, B. , Shah, S. , Telfer, S. , & Begon, M. (2015). Spatial analyses of wildlife contact networks. Journal of the Royal Society Interface, 12(102), 20141004 10.1098/rsif.2014.1004 PMC4277090 25411407

[ece35753-bib-0018] Delahay, R. J. , Cheeseman, C. L. , & Clifton‐Hadley, R. S. (2001). Wildlife disease reservoirs: The epidemiology of *Mycobacterium bovis* infection in the European badger (*Meles meles*) and other British mammals. Tuberculosis (Edinb), 81(1–2), 43–49. 10.1054/tube.2000.0266 11463223

[ece35753-bib-0019] Delahay, R. J. , Langton, S. , Smith, G. C. , Clifton‐Hadley, R. S. , & Cheeseman, C. L. (2000). The spatio‐temporal distribution of *Mycobacterium bovis* (bovine tuberculosis) infection in a high‐density badger population. Journal of Animal Ecology, 69(3), 428–441. 10.1046/j.1365-2656.2000.00406.x

[ece35753-bib-0020] Dugdale, H. L. , Macdonald, D. W. , Pope, L. C. , et al. (2008). Reproductive skew and relatedness in social groups of European badgers, *Meles meles* . Molecular Ecology, 17, 1815–1827. 10.1111/j.1365-294X.2008.03708.x 18371017

[ece35753-bib-0021] Dugdale, H. L. , Macdonald, D. W. , Pope, L. C. , & Burke, T. (2007). Polygynandry, extra‐group paternity and multiple‐paternity litters in European badger (*Meles meles*) social groups. Molecular Ecology, 16, 5294–5306. 10.1111/j.1365-294X.2007.03571.x 17971085

[ece35753-bib-0022] Eves, J. (1999). Impact of badger removal on bovine tuberculosis in east County Offaly. Irish Veterinary Journal, 52(4), 199–203.

[ece35753-bib-0023] Fowler, P. A. , & Racey, P. A. (1988). Overwintering strategies of the badger, *Meles meles*, at 57 N. Journal of Zoology, 214(4), 635–651. 10.1111/j.1469-7998.1988.tb03763.x

[ece35753-bib-0024] Frantz, A. C. , Do Linh San, E. , Pope, L. C. , & Burke, T. (2010). Using genetic methods to investigate dispersal in two badger (*Meles meles*) populations with different ecological characteristics. Heredity, 104(5), 493–501. 10.1038/hdy.2009.136 19812619

[ece35753-bib-0025] Gallagher, J. , & Clifton-Hadley, R. S. (2000). Tuberculosis in badgers; a review of the disease and its significance for other animals. Research in Veterinary Science, 69, 203–217. 10.1053/rvsc.2000.0422 11124091

[ece35753-bib-0026] Gaughran, A. (2018). The impact of roadworks on the ranging behaviour of European badgers (Meles meles), PhD Thesis. Dublin, Ireland: Trinity College Dublin.

[ece35753-bib-0027] Gaughran, A. , Kelly, D. J. , MacWhite, T. , Mullen, E. , Maher, P. , Good, M. , & Marples, N. M. (2018). Super‐ranging. A new ranging strategy in European badgers. PLoS ONE, 13(2), e0191818. 29444100 10.1371/journal.pone.0191818 PMC5812585

[ece35753-bib-0028] Godfray, H. C. , Donnelly, C. A. , Kao, R. R. , Macdonald, D. W. , McDonald, R. A. , Petrokofsky, G. , … McLean, A. R. (2013). A restatement of the natural science evidence base relevant to the control of bovine tuberculosis in Great Britain. Proceedings of the Royal Society B: Biological Sciences, 280(1768), 20131634 10.1098/rspb.2013.1634 PMC3757986 23926157

[ece35753-bib-0029] Gormley, E. , & Costello, E. (2003). Tuberculosis and badgers: New approaches to diagnosis and control. Journal of Applied Microbiology, 94(1s), 80S–86S. 10.1046/j.1365-2672.94.s1.9.x 12675939

[ece35753-bib-0030] Greenwood, P. J. (1980). Mating systems, philopatry and dispersal in birds and mammals. Animal Behaviour, 28(4), 1140–1162. 10.1016/S0003-3472(80)80103-5

[ece35753-bib-0031] Griffin, J. M. , Williams, D. H. , Kelly, G. E. , Clegg, T. A. , O'Boyle, I. , Collins, J. D. , & More, S. J. (2005). The impact of badger removal on the control of tuberculosis in cattle herds in Ireland. Preventive Veterinary Medicine, 67(4), 237–266. 10.1016/j.prevetmed.2004.10.009 15748755

[ece35753-bib-0032] Hancox, M. (1988). Field age determination in the European badger. Revue D'ecologie (La Terre et La Vie), 43, 399–404.

[ece35753-bib-0033] Huck, M. , Frantz, A. C. , Dawson, D. A. , Burke, T. , & Roper, T. J. (2008). Low genetic variability, female‐biased dispersal and high movement rates in an urban population of Eurasian badgers *Meles meles* . Journal of Animal Ecology, 77(5), 905–915. 18924251 10.1111/j.1365-2656.2008.01415.x

[ece35753-bib-0034] Hutchings, M. R. , & Harris, S. (1997). Effects of farm management practices on cattle grazing behaviour and the potential for transmission of bovine tuberculosis from badgers to cattle. The Veterinary Journal, 153(2), 149–162. 10.1016/S1090-0233(97)80035-4 12463400

[ece35753-bib-0035] Johnson, D. D. P. , Jetz, W. , & Macdonald, D. W. (2002). Environmental correlates of badger social spacing across Europe. Journal of Biogeography, 29(3), 411–425. 10.1046/j.1365-2699.2002.00680.x

[ece35753-bib-0036] Kaseda, Y. , Ogawa, H. , & Khalil, A. M. (1997). Causes of natal dispersal and emigration and their effects on harem formation in Misaki feral horses. Equine Veterinary Journal, 29(4), 262–266. 10.1111/j.2042-3306.1997.tb03121.x 15338905

[ece35753-bib-0037] Kruuk, H. , & Parish, T. (1987). Changes in the size of groups and ranges of the European badger (*Meles meles*) in an area in Scotland. The Journal of Animal Ecology, 56(1), 351–364.

[ece35753-bib-0038] Lloyd‐Smith, J. O. , Schreiber, S. J. , Kopp, P. E. , & Getz, W. M. (2005). Superspreading and the effect of individual variation on disease emergence. Nature, 438(7066), 355 10.1038/nature04153 16292310 PMC7094981

[ece35753-bib-0039] Macdonald, D. W. , Newman, C. , Buesching, C. D. , & Johnson, P. J. (2008). Male‐biased movement in a high‐density population of the Eurasian badger (*Meles meles*). Journal of Mammalogy, 89(5), 1077–1086. 10.1644/07-MAMM-A-185.1

[ece35753-bib-0040] Macdonald, D. W. , Riordan, P. , & Mathews, F. (2006). Biological hurdles to the control of TB in cattle: A test of two hypotheses concerning wildlife to explain the failure of control. Biological Conservation, 131(2), 268–286. 10.1016/j.biocon.2006.05.006

[ece35753-bib-0041] MacWhite, T. , Maher, P. , Mullen, E. , Marples, N. , & Good, M. (2013). Satellite tracking study of badgers (*Meles meles*) to establish normal ranging behaviour prior to a road realignment. The Irish Naturalists Journal, 32(2), 99–105.

[ece35753-bib-0042] Martin, S. W. , Eves, J. A. , Dolan, L. A. , Hammond, R. F. , Griffin, J. M. , Collins, J. D. , & Shoukri, M. M. (1997). The association between the bovine tuberculosis status of herds in the East Offaly Project Area, and the distance to badger setts, 1988–1993. Preventive Veterinary Medicine, 31(1–2), 113–125. 10.1016/S0167-5877(96)01111-7 9234430

[ece35753-bib-0043] McDonald, J. L. , Robertson, A. , & Silk, M. J. (2018). Wildlife disease ecology from the individual to the population: Insights from a long‐term study of a naturally infected European badger population. Journal of Animal Ecology, 87(1), 101–112. 10.1111/1365-2656.12743 28815647

[ece35753-bib-0044] Mullen, E. M. , MacWhite, T. , Maher, P. K. , Kelly, D. J. , Marples, N. M. , & Good, M. (2015). The avoidance of farmyards by European badgers *Meles meles* in a medium density population. Applied Animal Behaviour Science, 171, 170–176. 10.1016/j.applanim.2015.08.021

[ece35753-bib-0045] Murphy, D. , Gormley, E. , Costello, E. , O'Meara, D. , & Corner, L. A. L. (2010). The prevalence and distribution of *Mycobacterium bovis* infection in European badgers (*Meles meles*) as determined by enhanced post mortem examination and bacteriological culture. Research in Veterinary Science, 88(1), 1–5. 10.1016/j.rvsc.2009.05.020 19545882

[ece35753-bib-0046] Murphy, D. , O'Keeffe, J. J. , Martin, S. W. , Gormley, E. , & Corner, L. A. (2009). An assessment of injury to European badgers (*Meles meles*) due to capture in stopped restraints. Journal of Wildlife Diseases, 45(2), 481–490. 10.7589/0090-3558-45.2.481 19395757

[ece35753-bib-0047] O'Mahony, D. T. (2015). Badger (*Meles meles*) contact metrics in a medium‐density population. Mammalian Biology‐Zeitschrift Für Säugetierkunde, 80(6), 484–490. 10.1016/j.mambio.2015.07.002

[ece35753-bib-0048] Pope, L. C. , Domingo‐Roura, X. , Erven, K. , & Burke, T. (2006). Isolation by distance and gene flow in the Eurasian badger (*Meles meles*) at both a local and broad scale. Molecular Ecology, 15(2), 371–386. 10.1111/j.1365-294X.2005.02815.x 16448407

[ece35753-bib-0049] Revilla, E. , & Palomares, F. (2002). Spatial organization, group living and ecological correlates in low‐density populations of Eurasian badgers, *Meles meles* . Journal of Animal Ecology, 71, 497–512. 10.1046/j.1365-2656.2002.00617.x

[ece35753-bib-0050] Riordan, P. , Delahay, R. J. , Cheeseman, C. , Johnson, P. J. , & Macdonald, D. W. (2011). Culling‐induced changes in badger (*Meles meles*) behaviour, social organisation and the epidemiology of bovine tuberculosis. PLoS ONE, 6(12), e28904 10.1371/journal.pone.0028904 22194946 PMC3237560

[ece35753-bib-0051] Rogers, L. M. , Delahay, R. , Cheeseman, C. L. , Langton, S. , Smith, G. C. , & Clifton‐Hadley, R. S. (1998). Movement of badgers (*Meles meles*) in a high‐density population: Individual, population and disease effects. Proceedings of the Royal Society B: Biological Sciences, 265(1403), 1269–1276. 10.1098/rspb.1998.0429 PMC1689199 9718736

[ece35753-bib-0052] Roper, T. J. (2010). Badger. London, UK: Collins.

[ece35753-bib-0053] Roper, T. J. , Ostler, J. R. , & Conradt, L. (2003). The process of dispersal in badgers *Meles meles* . Mammal Review, 33(3‐4), 314–318. 10.1046/j.1365-2907.2003.00031.x

[ece35753-bib-0054] Rozins, C. , Silk, M. J. , Croft, D. P. , Delahay, R. J. , Hodgson, D. J. , McDonald, R. A. , … Boots, M. (2018). Social structure contains epidemics and regulates individual roles in disease transmission in a group‐living mammal. Ecology and Evolution, 8(23), 12044–12055. 10.1002/ece3.4664 30598798 PMC6303749

[ece35753-bib-0055] Scantlebury, M. , Hutchings, M. R. , Allcroft, D. J. , & Harris, S. (2004). Risk of disease from wildlife reservoirs: Badgers, cattle, and bovine tuberculosis. Journal of Dairy Science, 87(2), 330–339. 10.3168/jds.S0022-0302(04)73172-0 14762076

[ece35753-bib-0056] Silk, M. J. , Croft, D. P. , Delahay, R. J. , Hodgson, D. J. , Boots, M. , Weber, N. , & McDonald, R. A. (2017). Using social network measures in wildlife disease ecology, epidemiology, and management. BioScience, 67(3), 245–257. 10.1093/biosci/biw175 28596616 PMC5384163

[ece35753-bib-0057] Silk, M. J. , Weber, N. L. , Steward, L. C. , Hodgson, D. J. , Boots, M. , Croft, D. P. , … McDonald, R. A. (2018). Contact networks structured by sex underpin sex‐specific epidemiology of infection. Ecology Letters, 21(2), 309–318. 10.1111/ele.12898 29266710 PMC6849844

[ece35753-bib-0058] Sun, L. , Müller‐Schwarze, D. , & Schulte, B. A. (2000). Dispersal pattern and effective population size in the beaver. Canadian Journal of Zoology, 78(3), 393–398. 10.1139/z99-226

[ece35753-bib-0059] Van Apeldoorn, R. , Vink, J. , & Matyáštík, T. (2006). Dynamics of a local badger (*Meles meles*) population in the Netherlands over the years 1983–2001. Mammalian Biology‐Zeitschrift Für Säugetierkunde, 71(1), 25–38. 10.1016/j.mambio.2005.08.005

[ece35753-bib-0060] Vicente, J. , Delahay, R. J. , Walker, N. J. , & Cheeseman, C. L. (2007). Social organization and movement influence the incidence of bovine tuberculosis in an undisturbed high‐density badger *Meles meles* population. The Journal of Animal Ecology, 76(2), 348–360. 10.1111/j.1365-2656.2006.01199.x 17302842

[ece35753-bib-0061] Walton, Z. , Samelius, G. , Odden, M. , & Willebrand, T. (2018). Long‐distance dispersal in red foxes *Vulpes Vulpes* revealed by GPS tracking. European Journal of Wildlife Research, 64, 64 10.1007/s10344-018-1223-9

[ece35753-bib-0062] Weber, N. , Carter, S. P. , Dall, S. R. , Delahay, R. J. , McDonald, J. L. , Bearhop, S. , & McDonald, R. A. (2013). Badger social networks correlate with tuberculosis infection. Current Biology, 23(20), R915–R916. 10.1016/j.cub.2013.09.011 24156807

[ece35753-bib-0063] Woodroffe, R. , Donnelly, C. A. , Cox, D. R. , Gilks, P. , Jenkins, H. E. , Johnston, W. T. , … Watkins, G. H. (2009). Bovine tuberculosis in cattle and badgers in localized culling areas. Journal of Wildlife Diseases, 45(1), 128–143. 10.7589/0090-3558-45.1.128 19204342

[ece35753-bib-0064] Woodroffe, R. , Donnelly, C. A. , Ham, C. , Jackson, S. Y. , Moyes, K. , Chapman, K. , … Cartwright, S. J. (2016). Ranging behaviour of badgers *Meles meles* vaccinated with Bacillus Calmette Guerin. Journal of Applied Ecology, 54(3), 718–725. 10.1111/1365-2664.12837

[ece35753-bib-0065] Woodroffe, R. , & Macdonald, D. W. (1995). Female/female competition in European badgers *Meles meles*: Effects on breeding success. Journal of Animal Ecology, 64(1), 12–20. 10.2307/5823

[ece35753-bib-0066] Woodroffe, R. , Macdonald, D. W. , & da Silva, J. (1995). Dispersal and philopatry in the European Badger, *Meles meles* . Journal of Zoology, 237(2), 227–239. 10.1111/j.1469-7998.1995.tb02760.x

[ece35753-bib-0067] Zimmermann, F. , Breitenmoser‐Würsten, C. , & Breitenmoser, U. (2005). Natal dispersal of Eurasian lynx (*Lynx lynx*) in Switzerland. Journal of Zoology, 267(4), 381–395. 10.1017/S0952836905007545

