# Supplementary material for: Dispersal patterns in a medium‐density Irish badger population: Implications for understanding the dynamics of tuberculosis transmission
Source: Ecol Evol. 2019 Nov 13;9(23):13142–52. doi: 10.1002/ece3.5753 (PMC6912907; doi:10.1002/ece3.5753)
Supplement: Supplementary file 1 [file ECE3-9-13142-s001.pdf]

SI Figure 1. Dispersal Maps. Figures 1.1 to 1.16 illustrate dispersal GPS locations for all badgers that were wearing a collar during dispersal or when they attempted dispersal. Where the event itself was missed, GPS locations are illustrated for before and after dispersal.

M01's Dispersal: Adjacent Move

Dotted circle indicates natal group location, blue polygon indicates new group location

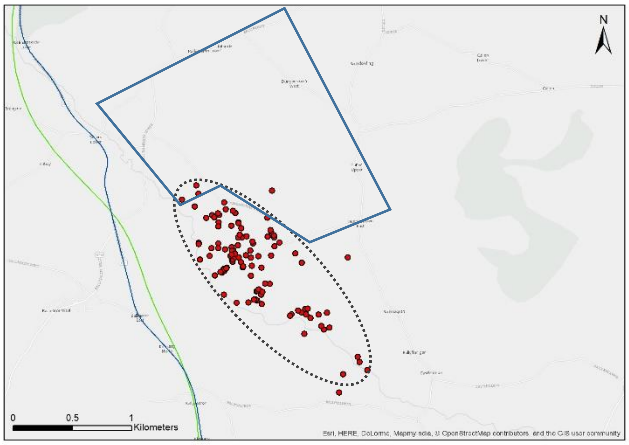

Figure 1.1a Pre-Dispersal 2010

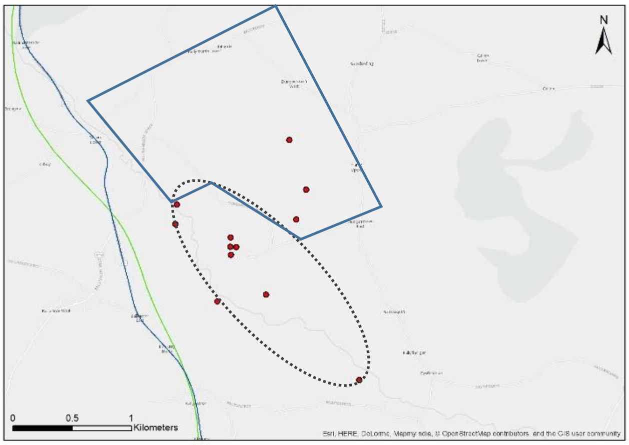

Figure 1.1b Dispersing January 2011

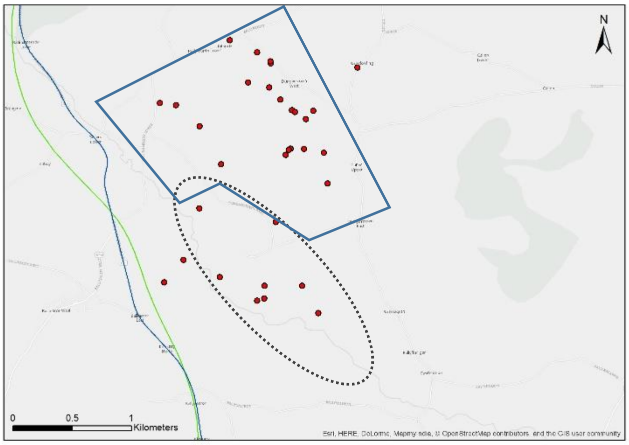

Figure 1.1c Dispersing February 2011, collar fails

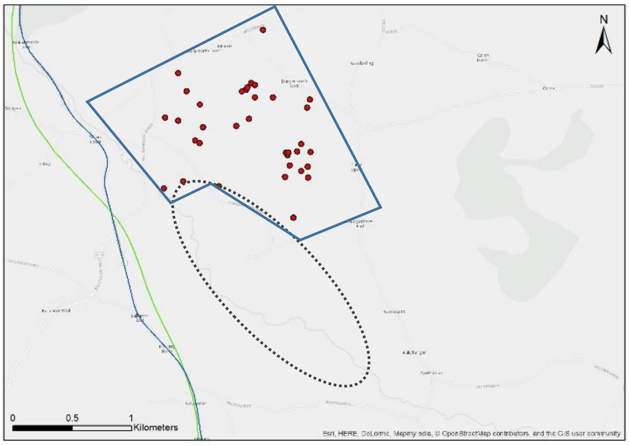

Figure 1.1d Dispersed Oct 2011, new collar.

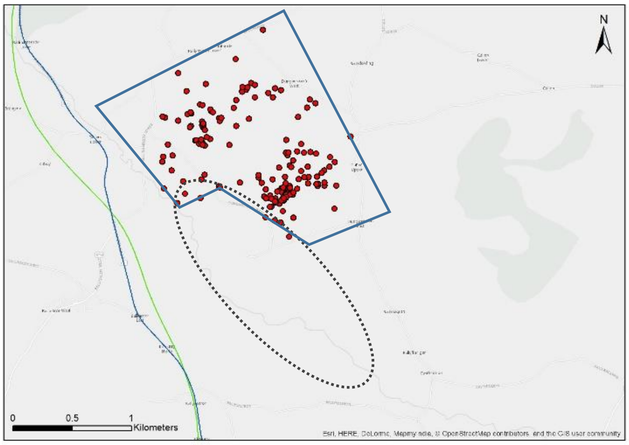

Figure 1.1e Dispersed Oct – Dec 2011

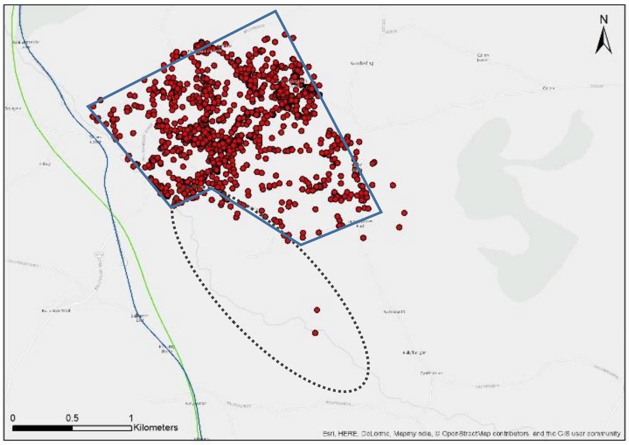

Figure 1.1f Post-Dispersal 2012

F03's Dispersal: Adjacent Move

Dotted circle indicates natal group location, blue polygon indicates new group location

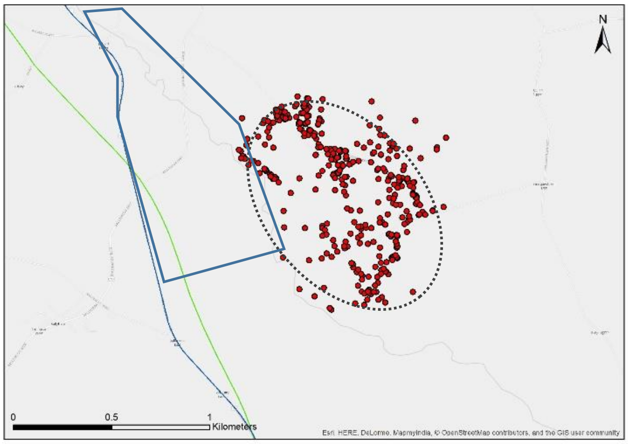

Figure 1.2a Pre-Dispersal Aug - Dec 2011

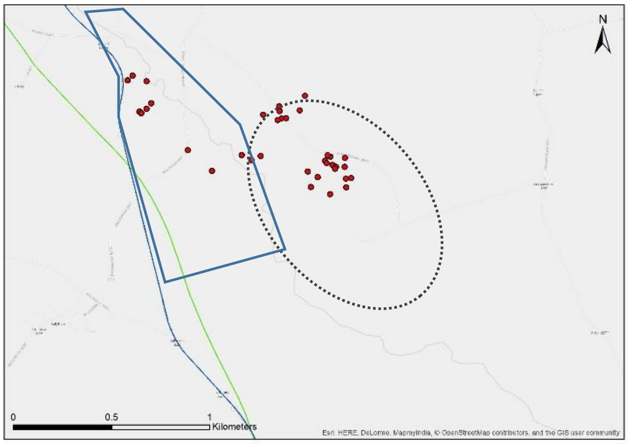

Figure 1.2b Dispersing January 2012, collar fails

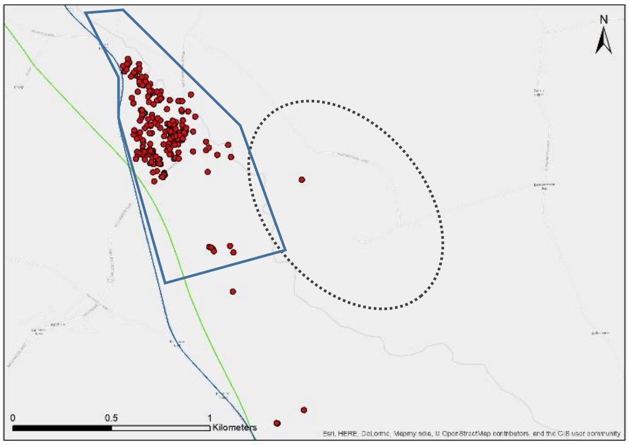

Figure 1.2c Dispersed, Oct – Dec 2012, new collar

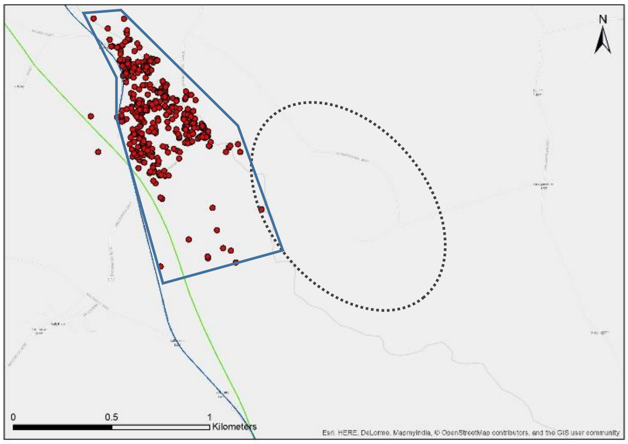

Figure 1.2d Post-Dispersal 2013.

M02's Exploratory Forays: Non-Adjacent Move. Event Missed

Dotted circle indicates natal group location, blue polygon indicates new group location.

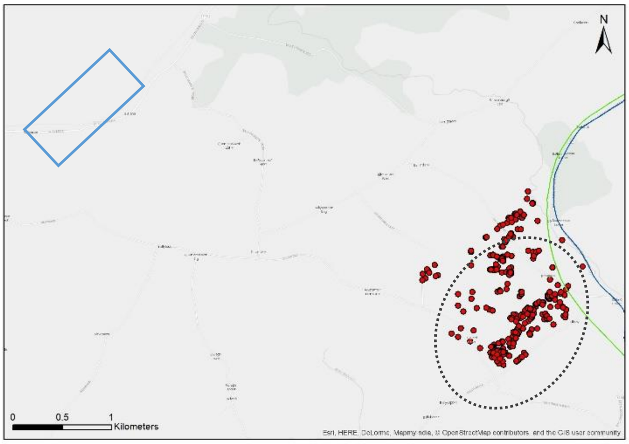

Figure 1.3a Normal Range 2013

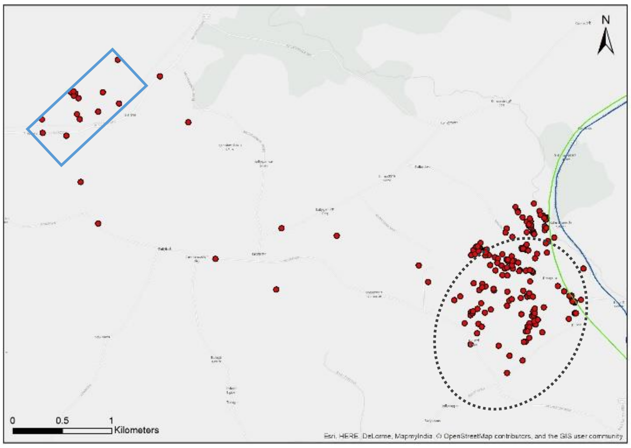

Figure 1.2b Exploring June & July 2013, collar fails
Shot in DAFM restraint 14/11/14 inside polygon.

F05's Dispersal: Adjacent Move, Event Missed

Dotted circle indicates natal group location, blue polygon indicates new group location.
Social group boundaries altered.

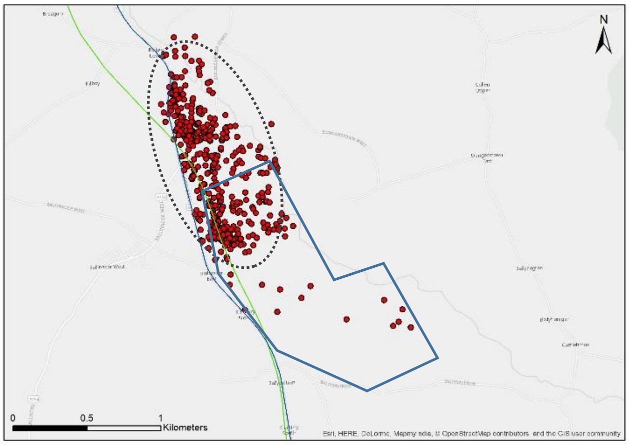

Figure 1.4a Pre-Dispersal 2010

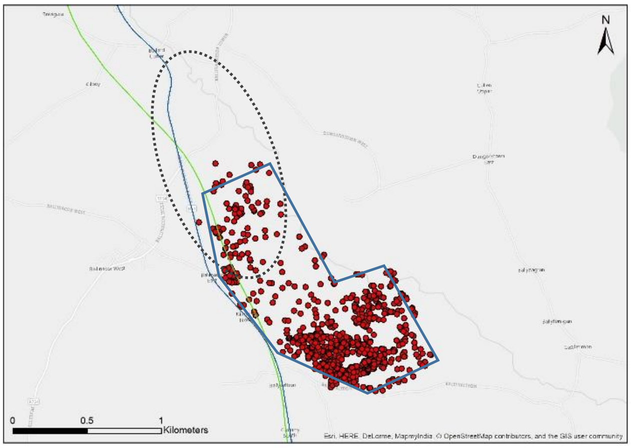

Figure 1.4b Post-Dispersal 2011. Retains a portion of natal social group's range

F06's Dispersal: Non-Adjacent Move, Event Missed

Dotted circle indicates natal group location, blue polygon indicates new group location.

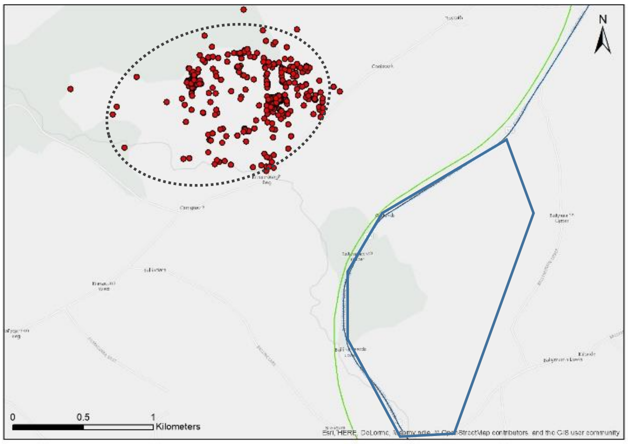

Figure 1.5a Pre-Dispersal 2012

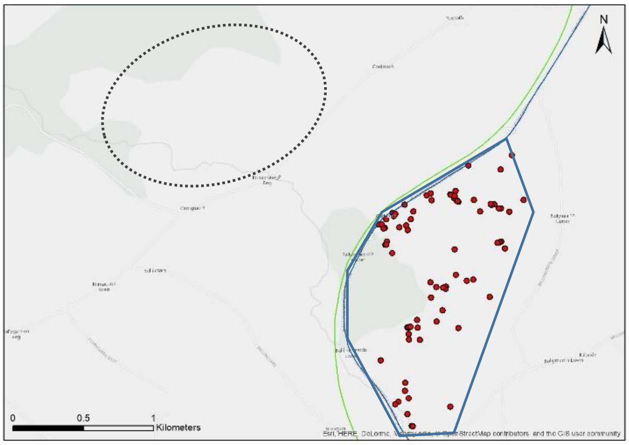

Figure 1.5b Post-Dispersal 2013.

M04’s Dispersal: Adjacent Move

Dotted circle indicates natal group location, blue polygon indicates new group location
Orange polygon indicates extent of super-range

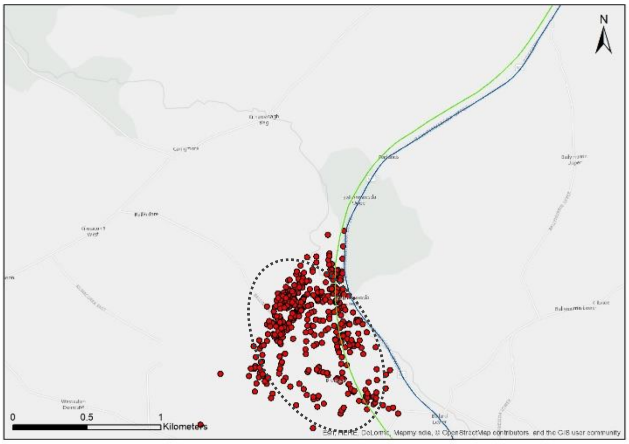

Figure 1.6a Pre-Dispersal 2012

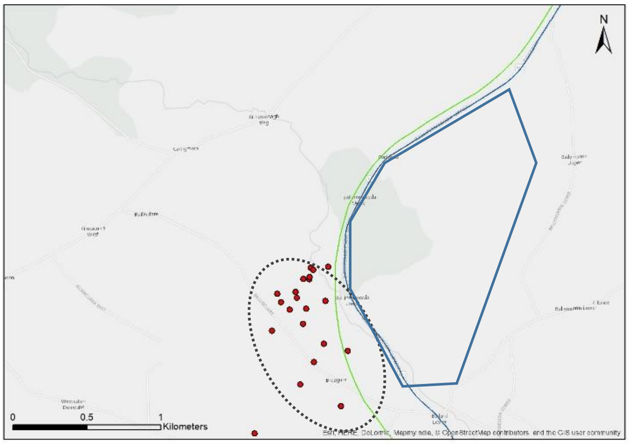

Figure 1.6b Pre-Dispersal January 2013

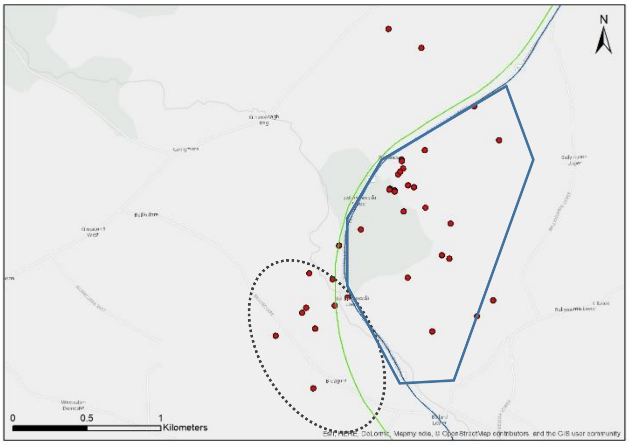

Figure 1.6c Dispersing February 2013

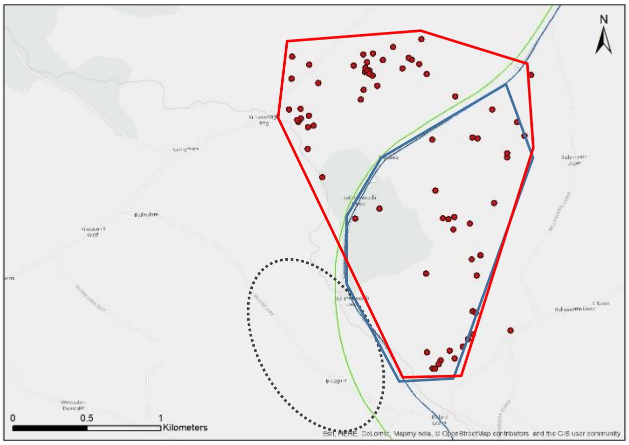

Figure 1.6d Dispersed March 2013. Becomes a super-ranger (orange polygon).

M07’s Dispersal: Adjacent Move

Dotted circle indicates natal group location, blue polygon indicates new group location

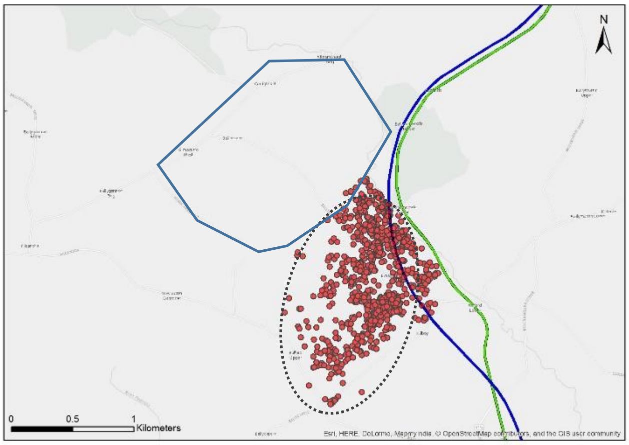

Figure 1.7a Pre-Dispersal 2014

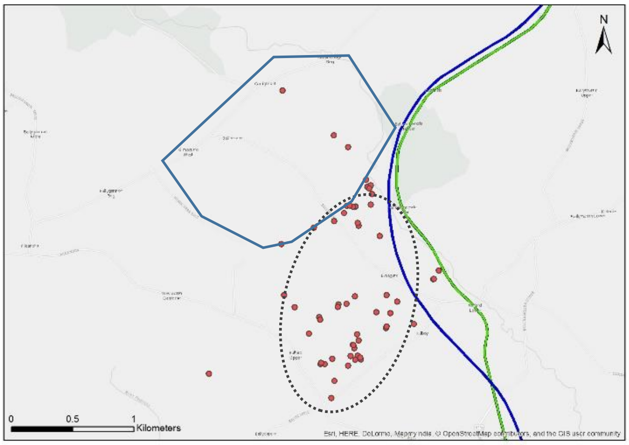

Figure 1.7b Dispersing January 2015

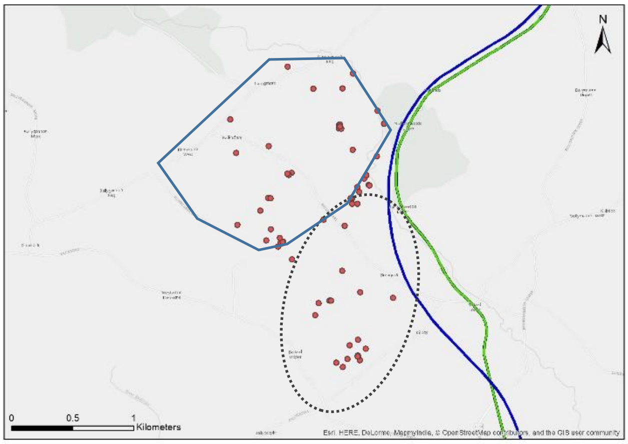

Figure 1.7c Dispersing February 2015

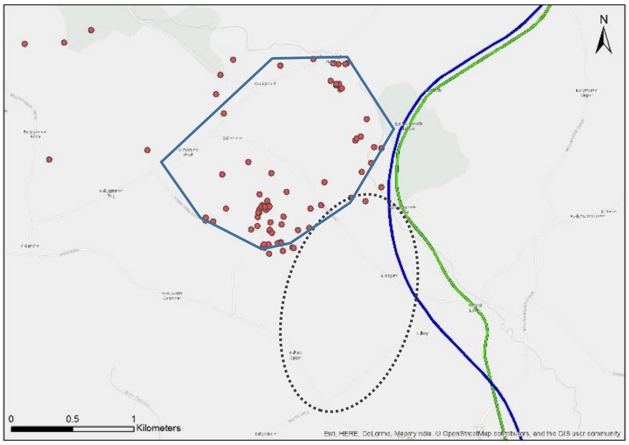

Figure 1.7d Dispersing March 2015

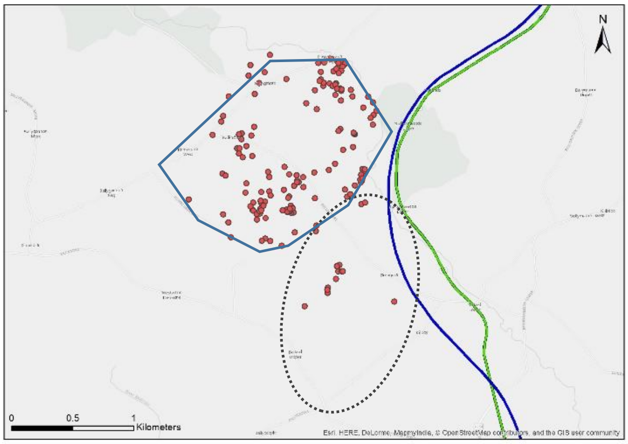

Figure 1.7e Dispersing April 2015

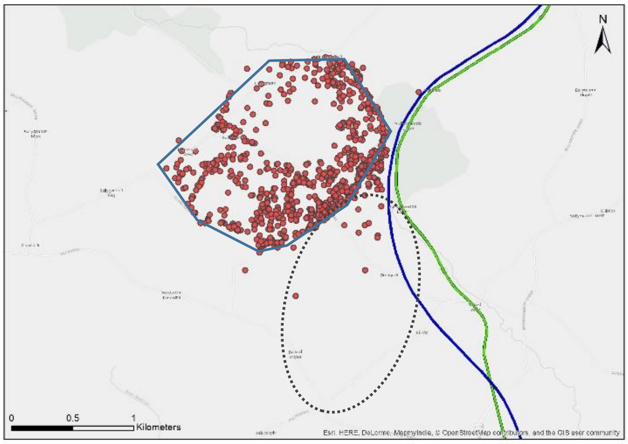

Figure 1.7f Post-Dispersal 2016.

M08's Dispersal: Adjacent Move

Dotted circle indicates natal group location, blue polygon indicates new group location

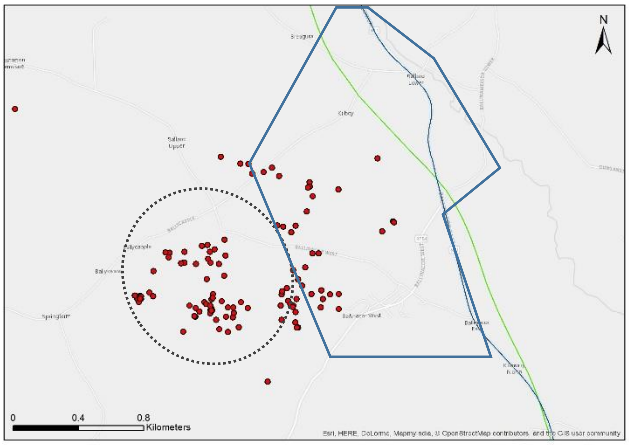

Figure 1.8a Dispersing April 2015, originally (2014) only trapped within natal social group

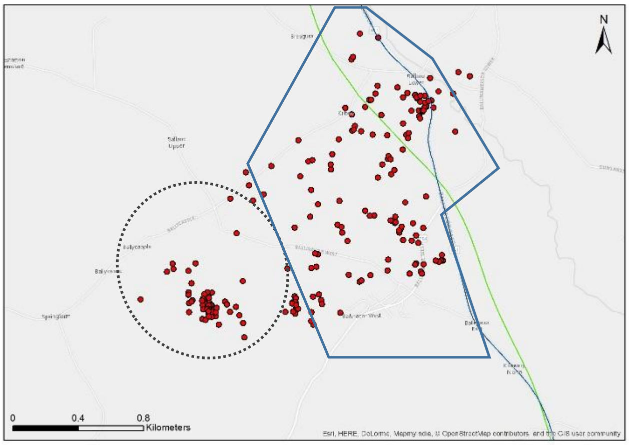

Figure 1.8b Dispersing May 2015

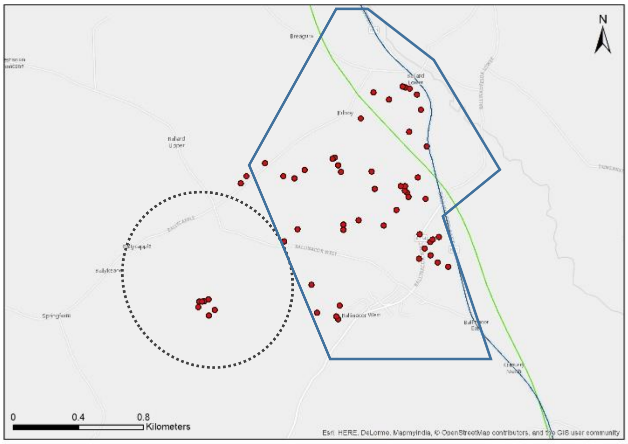

Figure 1.8c Dispersing June 2015, collar fails

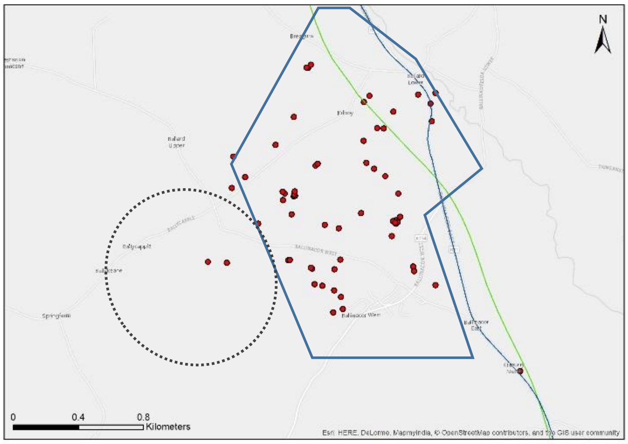

Figure 1.8d Dispersed October 2015

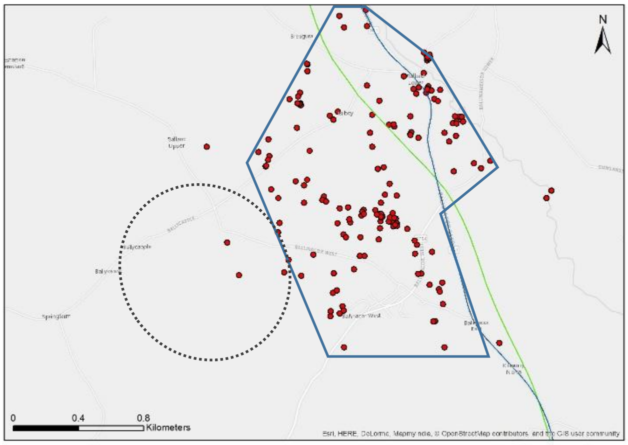

Figure 1.8e Dispersed 2016.

F09's Dispersal: Non-Adjacent Move

Dotted circle indicates natal group location, blue polygon indicates new group location

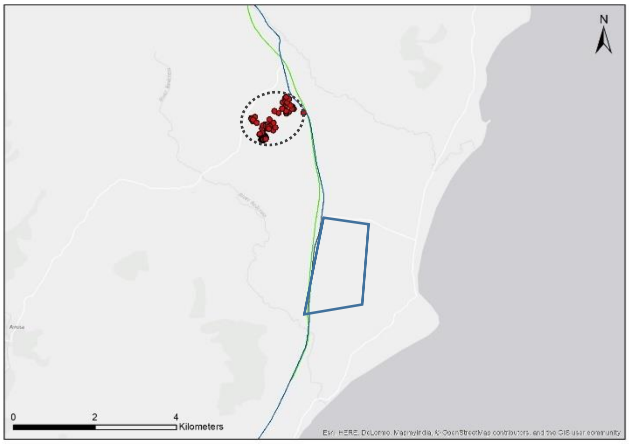

Figure 1.9a Pre-Dispersal Oct-Dec 2012

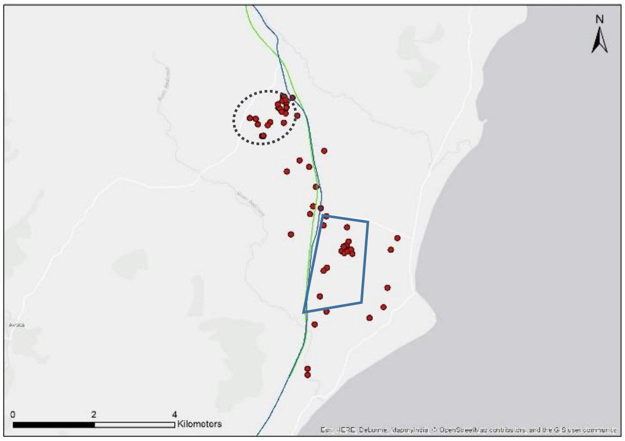

Figure 1.9b Dispersing January 2013

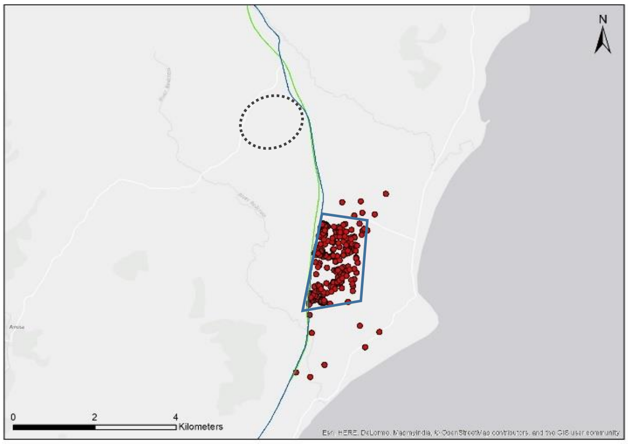

Figure 1.9c Post-Dispersal Feb – June 2013.

F08's Exploratory Forays

Natal social group indicated by dotted circle

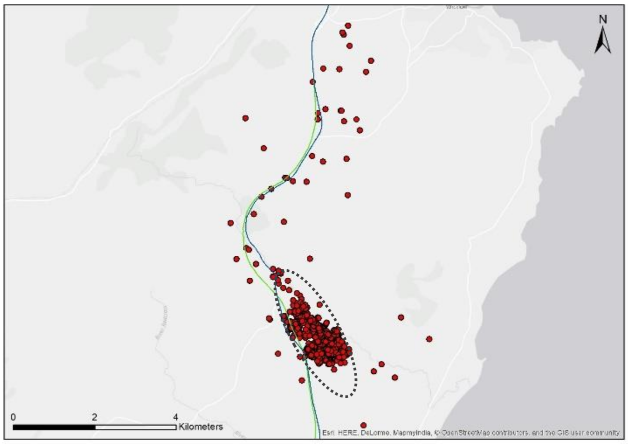

Figure 1.10. Exploring Feb –May 2016 but did not disperse. Contact lost January 2017.

F10’s Dispersal: Non-Adjacent Move

Dotted circle indicates natal group location, blue polygon indicates new group location

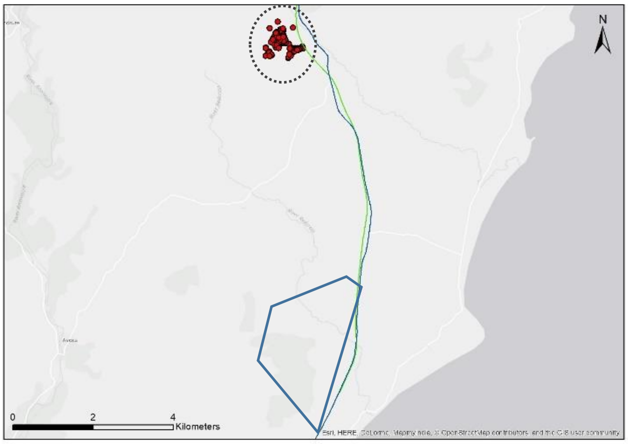

Figure 1.11a Pre-Dispersal 2014

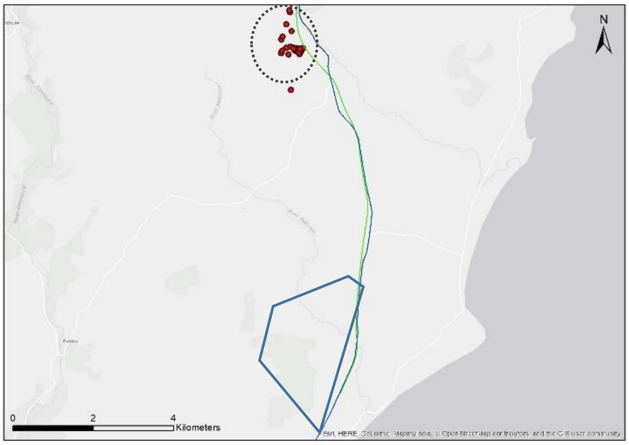

Figure 1.11b Dispersing January 2015

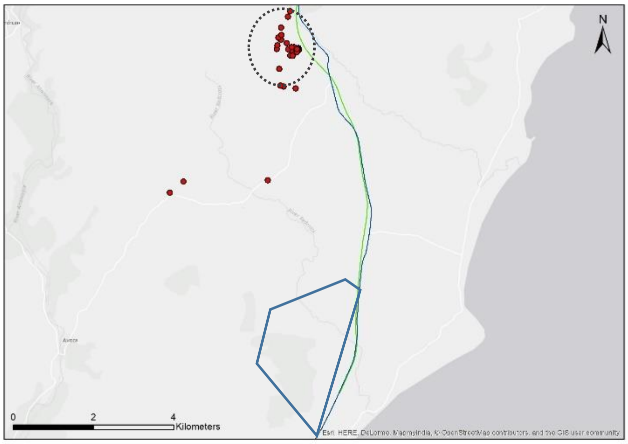

Figure 1.11c Dispersing February 2015

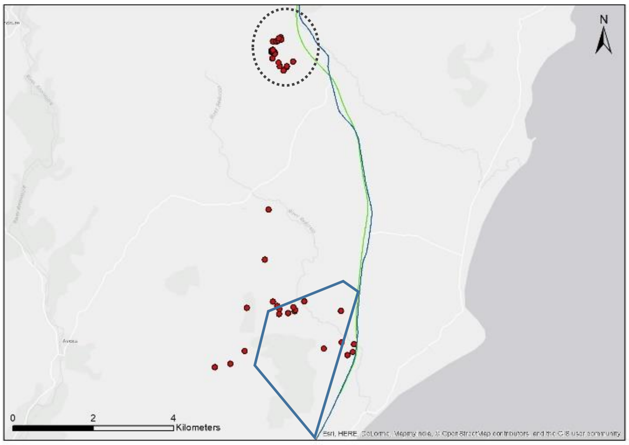

Figure 1.11d Dispersing March 2015

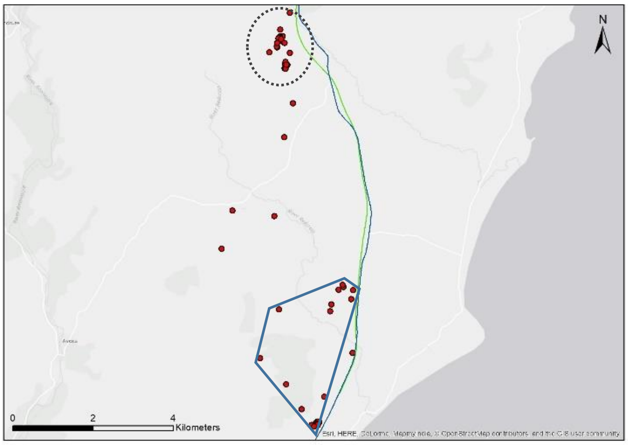

Figure 1.11e Dispersing April 2015, dies in fox snare at southern-most points.

F11's Dispersal: Non-Adjacent Move

Dotted circle indicates natal group location, blue polygon indicates new group location
Wanders over vast area before settling

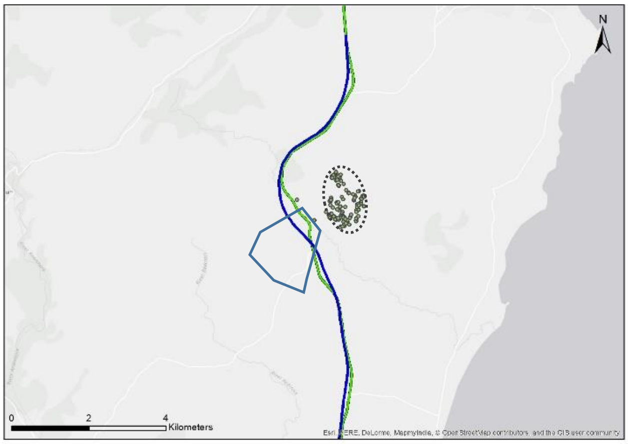

Figure 1.12a Pre-Dispersal 2014

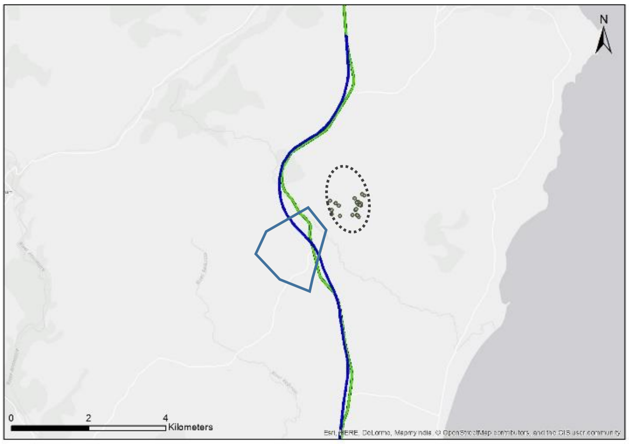

Figure 1.12b Pre-Dispersal January 2015

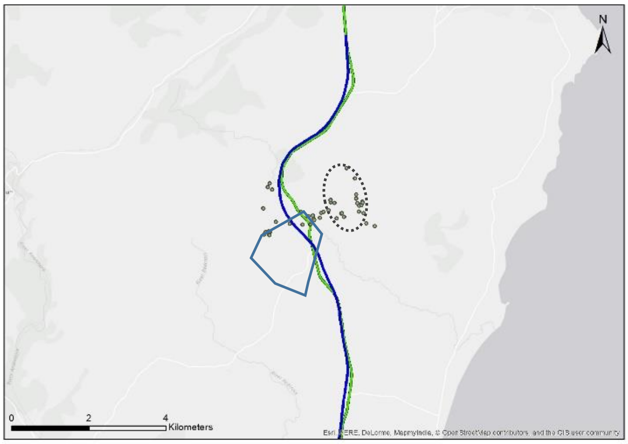

Figure 1.12c Dispersing February 2015

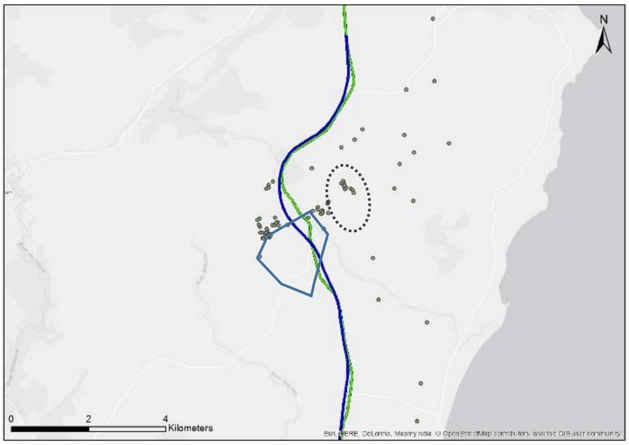

Figure 1.12d Dispersing March 2015

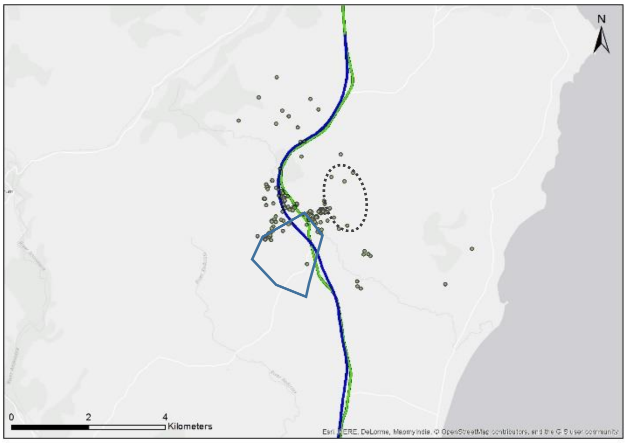

Figure 1.12e Dispersing April 2015

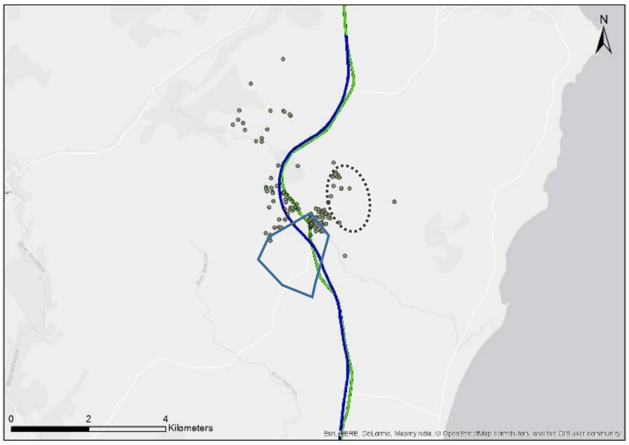

Figure 1.12f Dispersing May 2015

F11’s Dispersal: Non-Adjacent Move Continued.

Dotted circle indicates natal group location, blue polygon indicates new group location

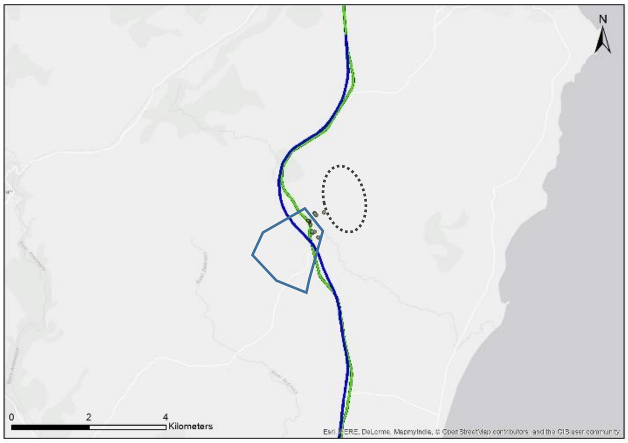

Figure 1.12g Settles temporarily June 2015 in edge of new social group.

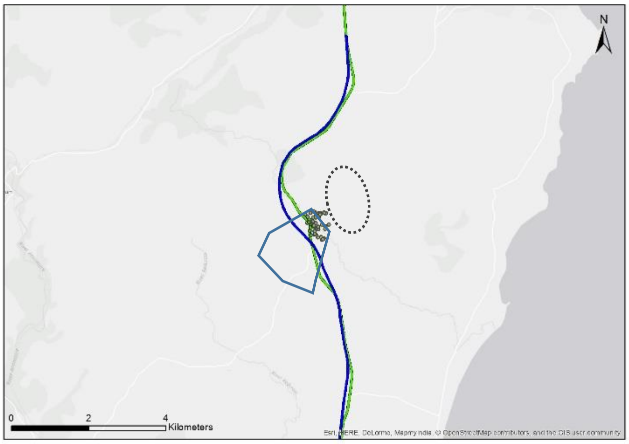

Figure 1.12h Settles temporarily July 2015 in edge of new social group.

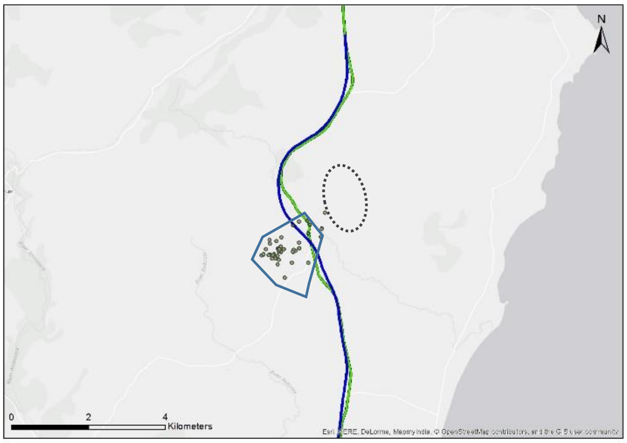

Figure 1.12i Disperses fully into new social group August 2015, upon death of resident dominant female

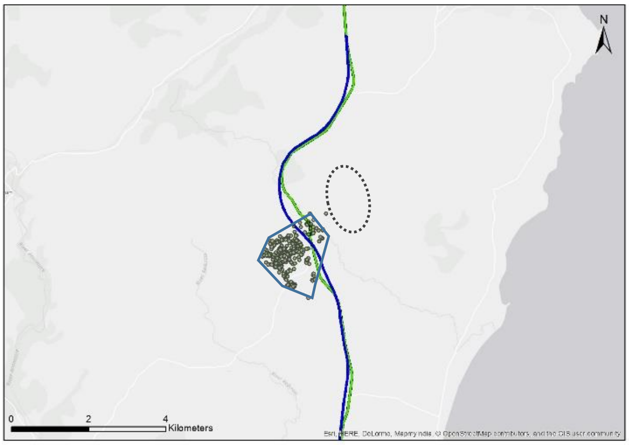

Figure 1.12j Dispersed Sep - Dec 2015.

M09: Immigrant

Location of The Bracken social group in blue polygon

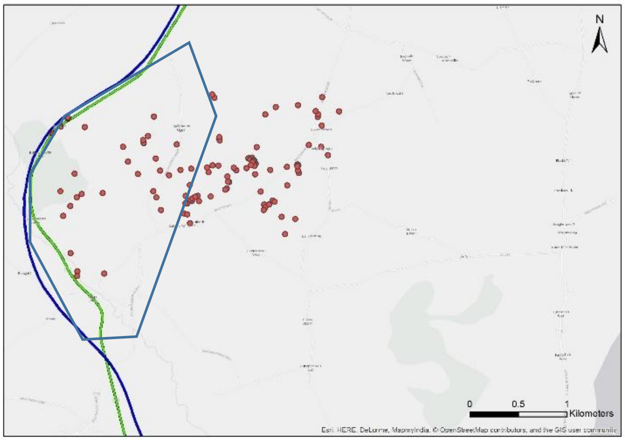

Figure 1.13a June 2016

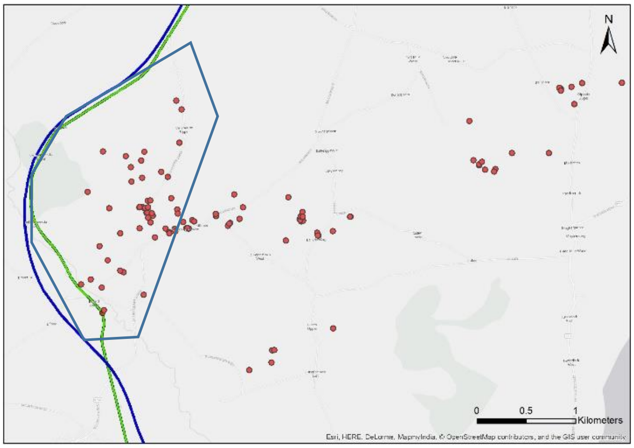

Figure 1.13b July 2016

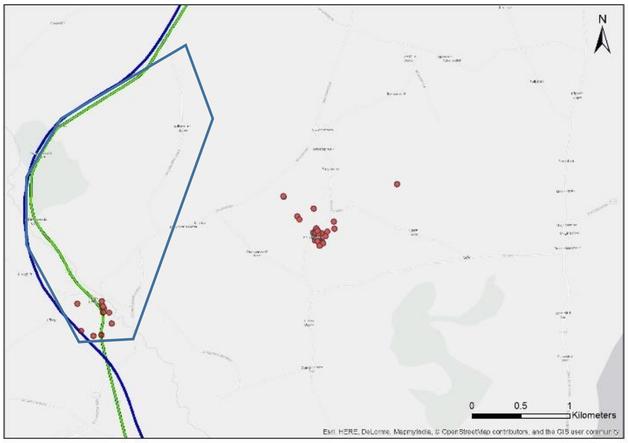

Figure 1.13c Aug 2016.

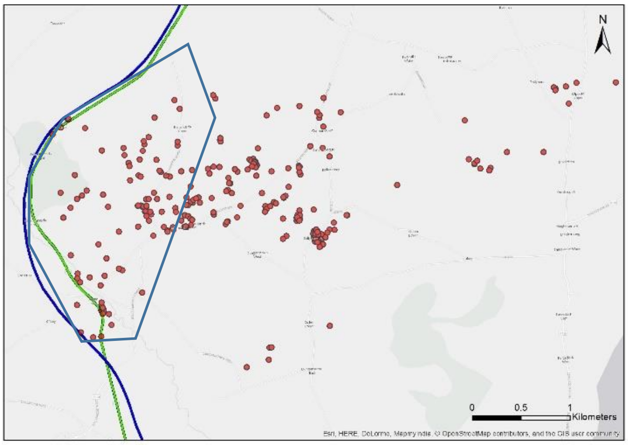

Figure 1.13d All GPS locations.

F10's Dispersal: Adjacent Move. Event Missed.

Social group boundaries altered.

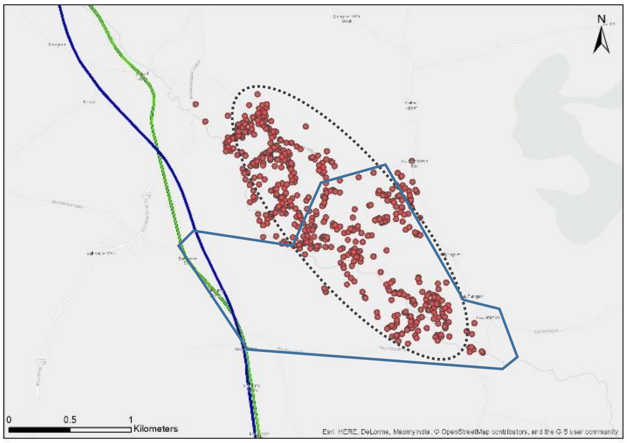

Figure 1.14a Pre-dispersal 2010

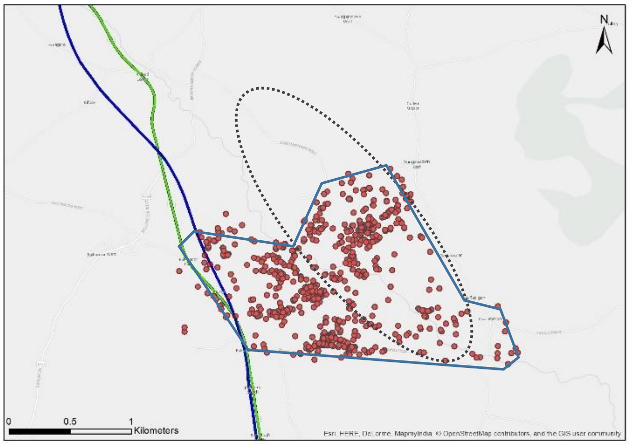

Figure 1.14b Post-Dispersal 2013. Fission of natal social group (dotted circle). Super-Ranging

F12’s Dispersal: Non-Adjacent Move

Dotted circle indicates natal group location, blue polygon indicates new group location

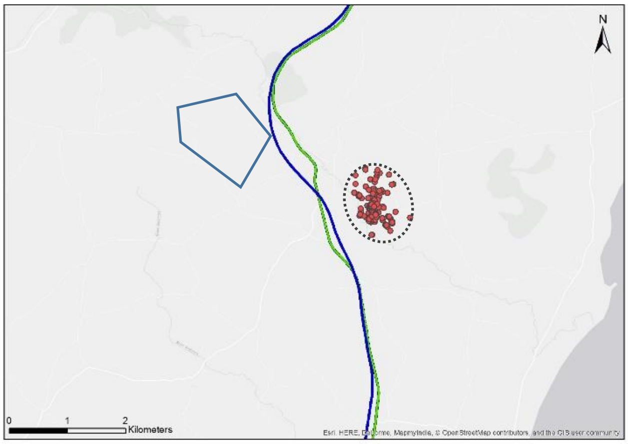

Figure 1.15a Pre-Dispersal Oct-Dec 2015

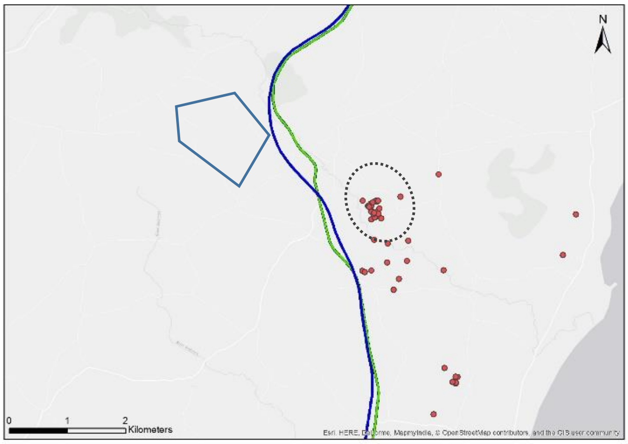

Figure 1.15b Dispersing January 2016

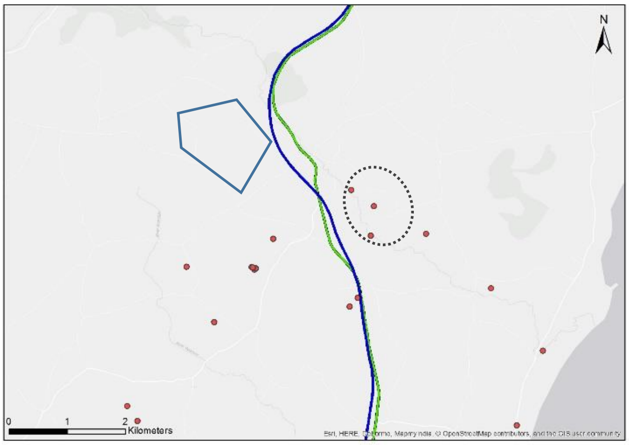

Figure 1.15c Dispersing February 2016

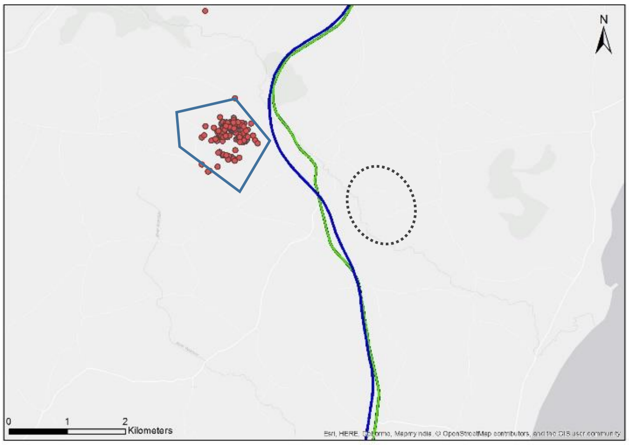

Figure 1.15d Dispersed May 2016

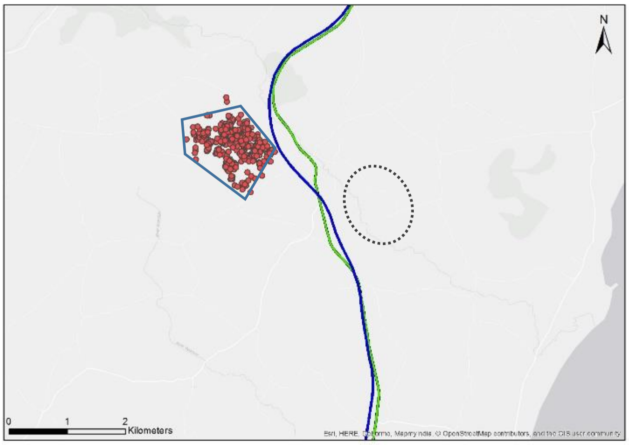

Figure 1.15e Dispersed June – Aug 2016.
Natal social group indicated by dotted circle

M13’s Dispersal: Adjacent Move

Dotted circle indicates natal group location, blue polygon indicates new group location

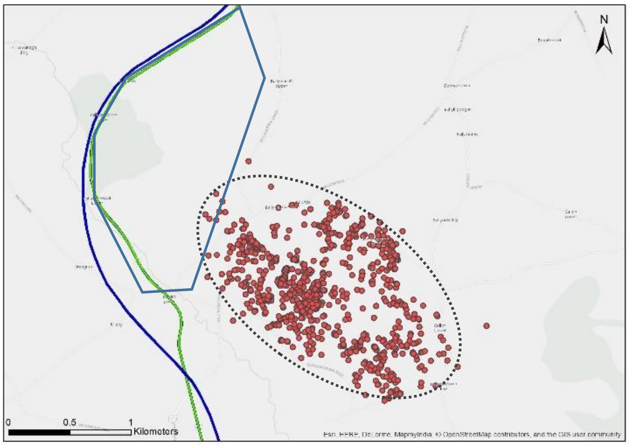

Figure 1.16a Pre-Dispersal 2010

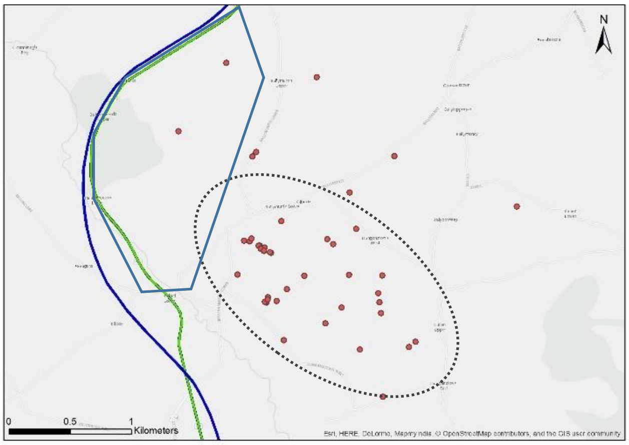

Figure 1.16b Dispersing January 2011

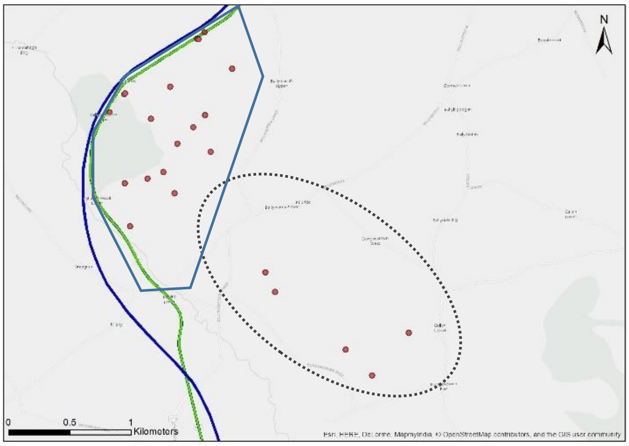

Figure 1.16c Dispersing February 2011, dies in RTA. Natal social group indicated by dotted circle
