# Supplementary material for: Dispersal patterns in a medium‐density Irish badger population: Implications for understanding the dynamics of tuberculosis transmission
Source: Ecol Evol. 2019 Nov 13;9(23):13142–52. doi: 10.1002/ece3.5753 (PMC6912907; doi:10.1002/ece3.5753)
Supplement: Supplementary file 2 [file ECE3-9-13142-s002.pdf]

SI Figure 2. Dispersal trajectories. Figures 2.1 to 2.13 illustrate dispersal trajectories. Figures 2.14 to 2.20 illustrate pre- and post-dispersal locations for badgers not wearing collars during dispersal.

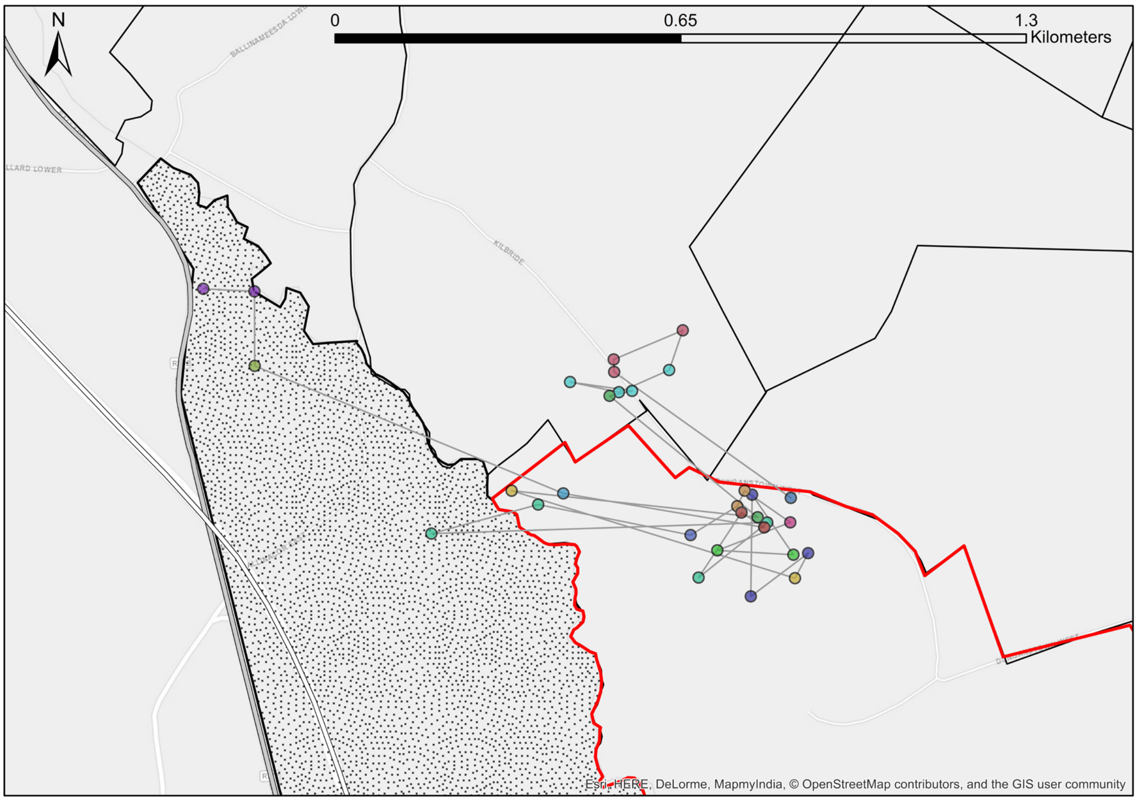

Figure 2.1 F03's dispersal trajectory (incomplete). January 2012. Extra-territorial excursions were made on in January. However, GPS data is patchy either as a result of reduced activity due to winter lethargy or a faulty collar so it is impossible to say if these were single or multiple-night excursions. The collar failed to send any further locations after 30th January. Initially visits were to a different neighbouring social group, but then she began to visit the new social group, where she was re-trapped and collared the following autumn, confirming dispersal. In January, she travelled 6 km through an area of approximately 0.4 km^2, visiting approximately additional 1 social group.

Filled circles represent GPS locations, with different colours indicating different nights. Thin grey lines join consecutive GPS locations. The thick red line outlines the natal territory boundary, the stippled area represents the new territory, and thin black lines outline other territory boundaries. The average distance between main setts in the study area was 1.3 km^2.

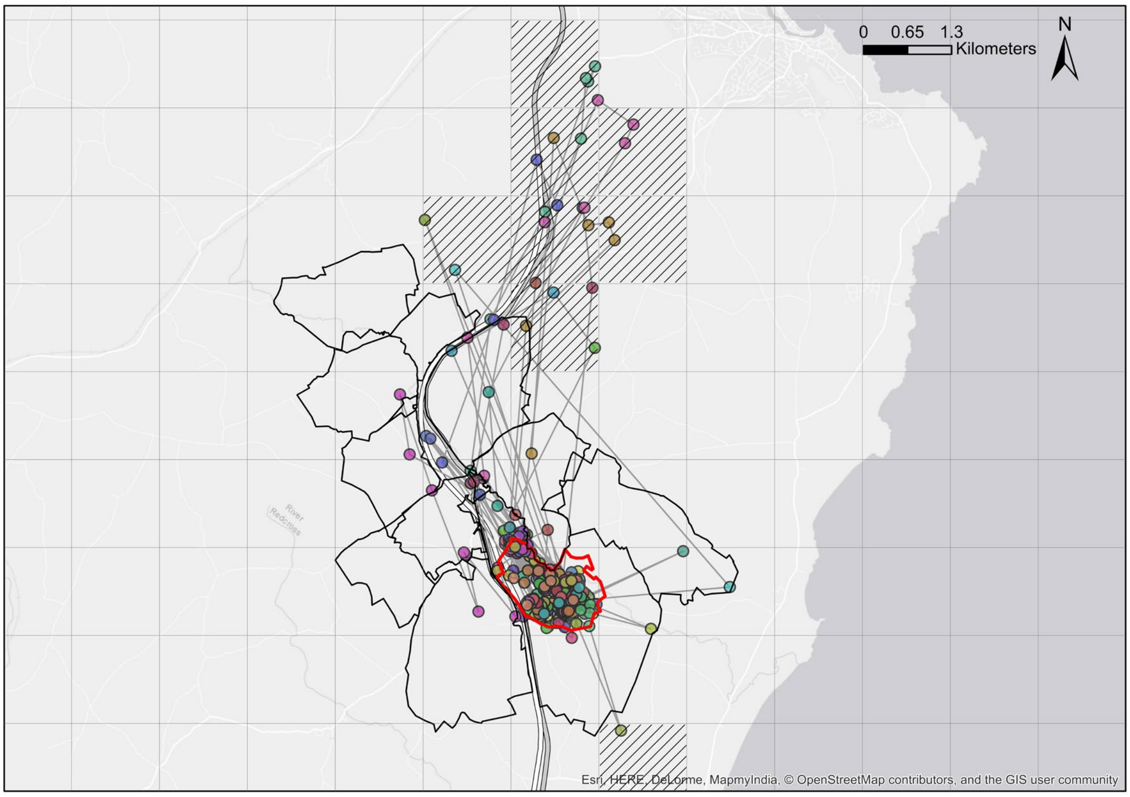

Figure 2.2 F08's exploratory trajectory. F08 made several exploratory forays between February and May 2016. She did not disperse at this time. However, contact was lost with F08 in January 2017, as a result of either death or successful dispersal. During this time, she travelled 272 km through an area of approximately 18 km^2, passing through approximately 16 different social groups.

Filled circles represent GPS locations, with different colours indicating different nights. Thin grey lines join consecutive GPS locations. The thick red line outlines the natal territory boundary, and thin black lines outline other territory boundaries. The average distance between main setts in the study area was 1.3 km$_2$. A grid of 1.3 km$_2$ is used as a proxy for social group territories outside of the study area.

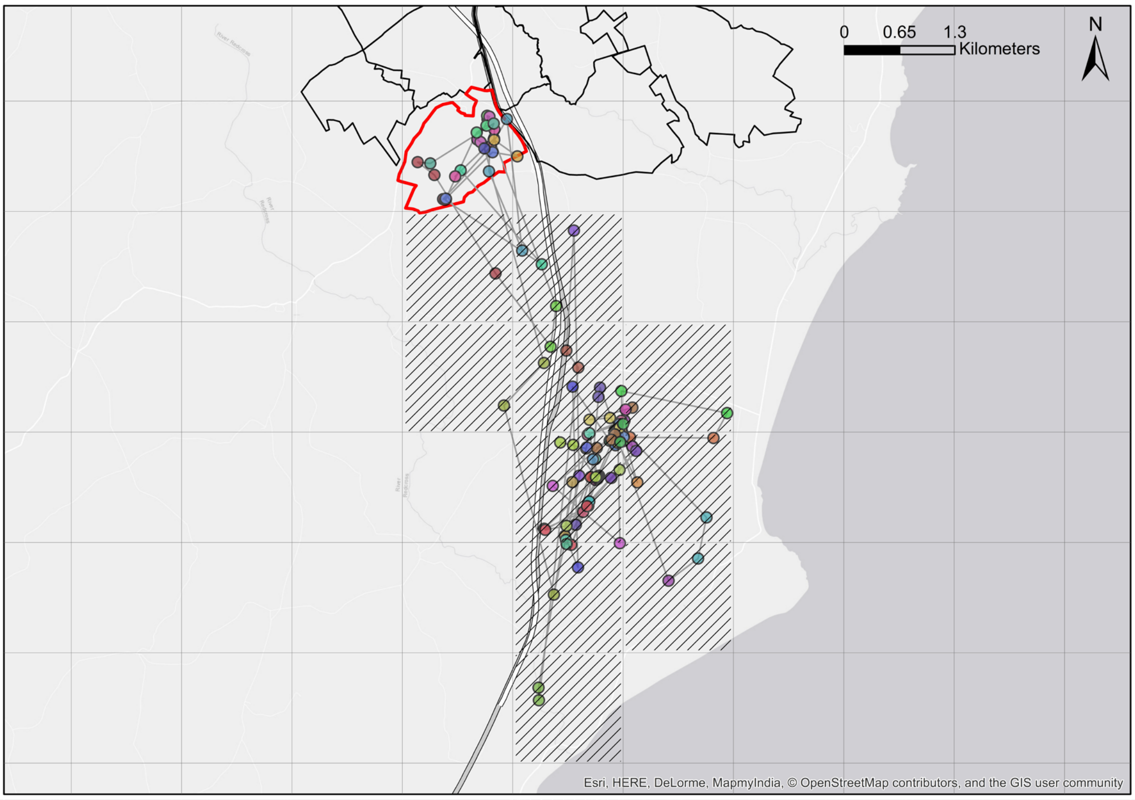

Figure 2.3 F09's dispersal trajectory. January and February 2013. F09 made three extra-territorial excursions before leaving her natal territory permanently on 16th of January. She appeared to explore before settling down in February. In this time, she travelled 75 km through an area of approximately 6 km2, passing through approximately 9 social groups' territories before settling down.

Filled circles represent GPS locations, with different colours indicating different nights. Thin grey lines join consecutive GPS locations. The thick red line outlines the natal territory boundary and thin black lines outline other territory boundaries. The average distance between main setts in the study area was 1.3 km2. A grid of 1.3 km2 is used as a proxy for social group territories outside of the study area. Quadrants with GPS locations are highlighted using a hatched pattern.

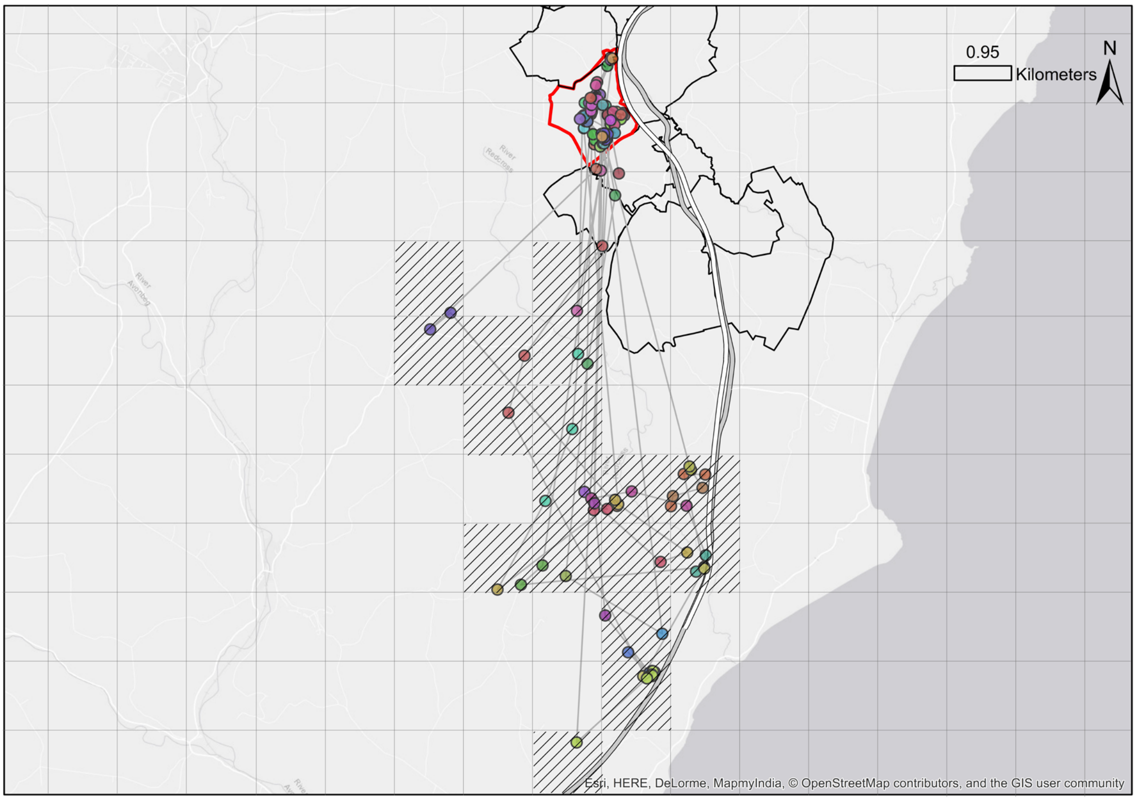

Figure 2.4 F10's dispersal trajectory. February - May 2015. Gradual move to a non-adjacent social group to the south, outside of the study area. Exploratory forays were made over multiple nights where she did not return to the natal social group but spent the day in the setts of other social groups. During this period, she travelled at least 147 km through an area of approximately 26 km^2, passing through approximately 19 social groups' territories. However, her collar was only working intermittently, so this is an underestimate. She recorded one round-trip of 21 km, where she spent at least two full nights away from her natal group. She was found dead before completing dispersal.

Filled circles represent GPS locations, with different colours indicating different nights. Thin grey lines join consecutive GPS locations. The thick red line outlines the natal territory boundary and thin black lines outline other territory boundaries. The average distance between main setts in the study area was 1.3km^2. A grid of 1.3km^2 is used as a proxy for social group territories outside of the study area. The red arrow indicates where she was found dead.

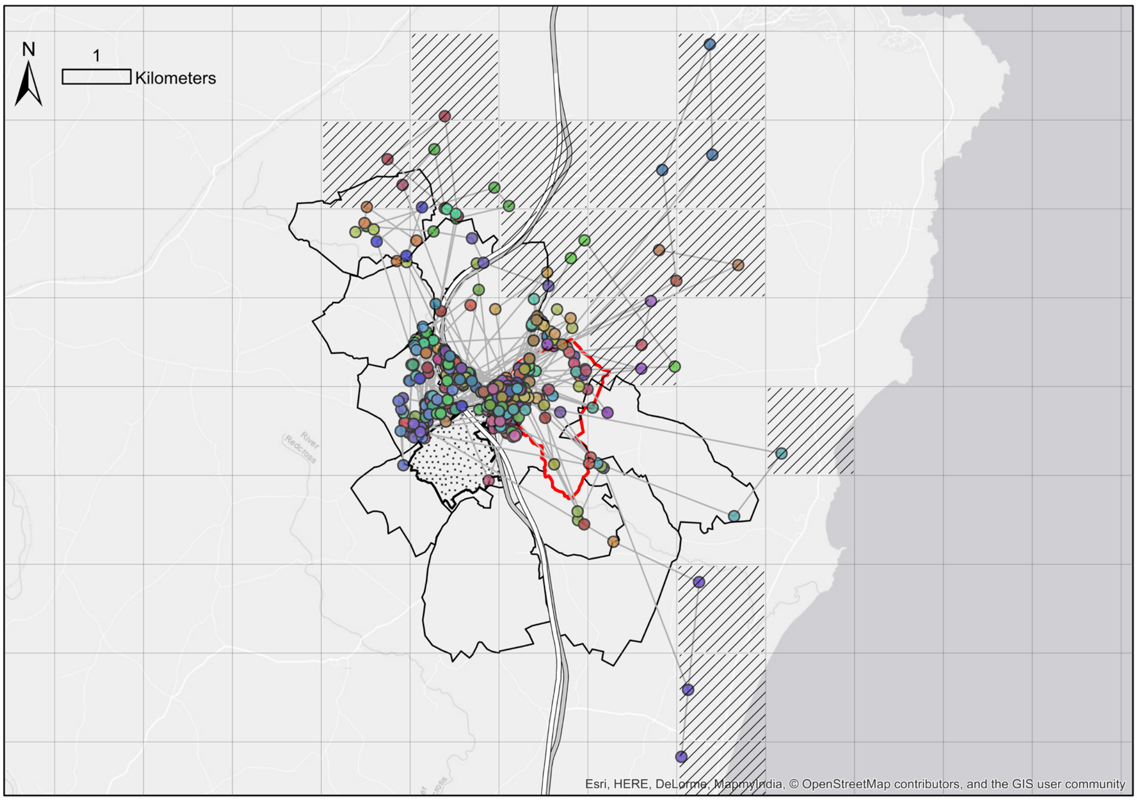

Figure 2.5 Trajectory of F11's exploratory ETEs prior to dispersal. Exploratory forays spanning February to July 2015, preceding a move to a non-adjacent social group 1.5 km away from her natal territory. During this time, F11 wandered widely, and travelled 307 km through an area of approximately 25 km^2, passing through approximately 22 social groups' territories, before she moved into her new territory following the death of the resident female.

Filled circles represent GPS locations, with different colours indicating different nights. Thin grey lines join consecutive GPS locations. The thick red line outlines the natal territory boundary and thin black lines outline other territory boundaries. The stippled area represents the territory she dispersed to. The average distance between main setts in the study area was 1.3km^2. A grid of 1.3km^2 is used as a proxy for social group territories outside of the study area.

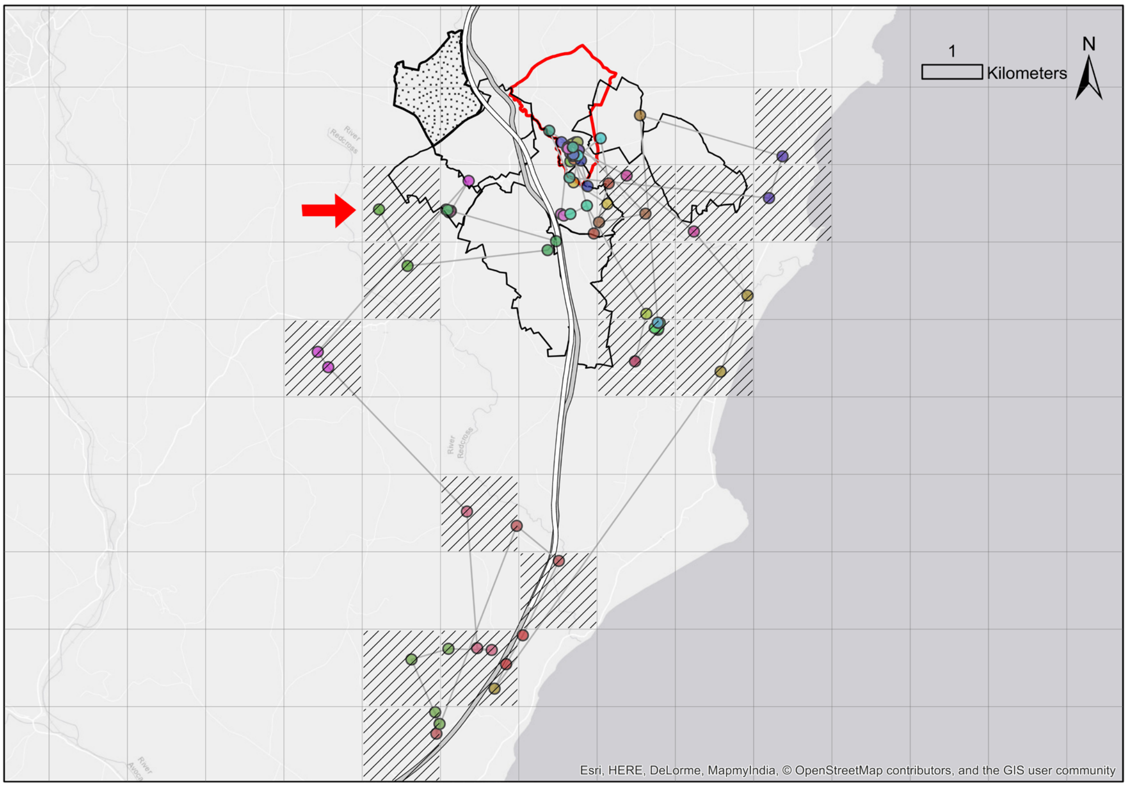

Figure 2.6 F12's dispersal trajectory. January and February 2016. Exploratory forays preceding an eventual move to a non-adjacent social group to the west (black dotted polygon, collar died before she settled). ETEs were made over multiple nights where F12 did not return to the natal social group but spent the day in the setts of other social groups. During this time, F12 travelled 66 km through an area of 40 km^2, passing through approximately 20 social groups' territories. However, her GPS collar failed before she completed dispersal, so this is an underestimate.

Filled circles represent GPS locations, with different colours indicating different nights. Thin grey lines join consecutive GPS locations. The thick red line outlines the natal territory boundary and thin black lines outline other territory boundaries. The stippled area represents the territory she dispersed to. The average distance between main setts in the study area was 1.3 km^2. A grid of 1.3 km^2 is used as a proxy for social group territories outside of the study area. The red arrow indicates the last GPS location received before collar failure.

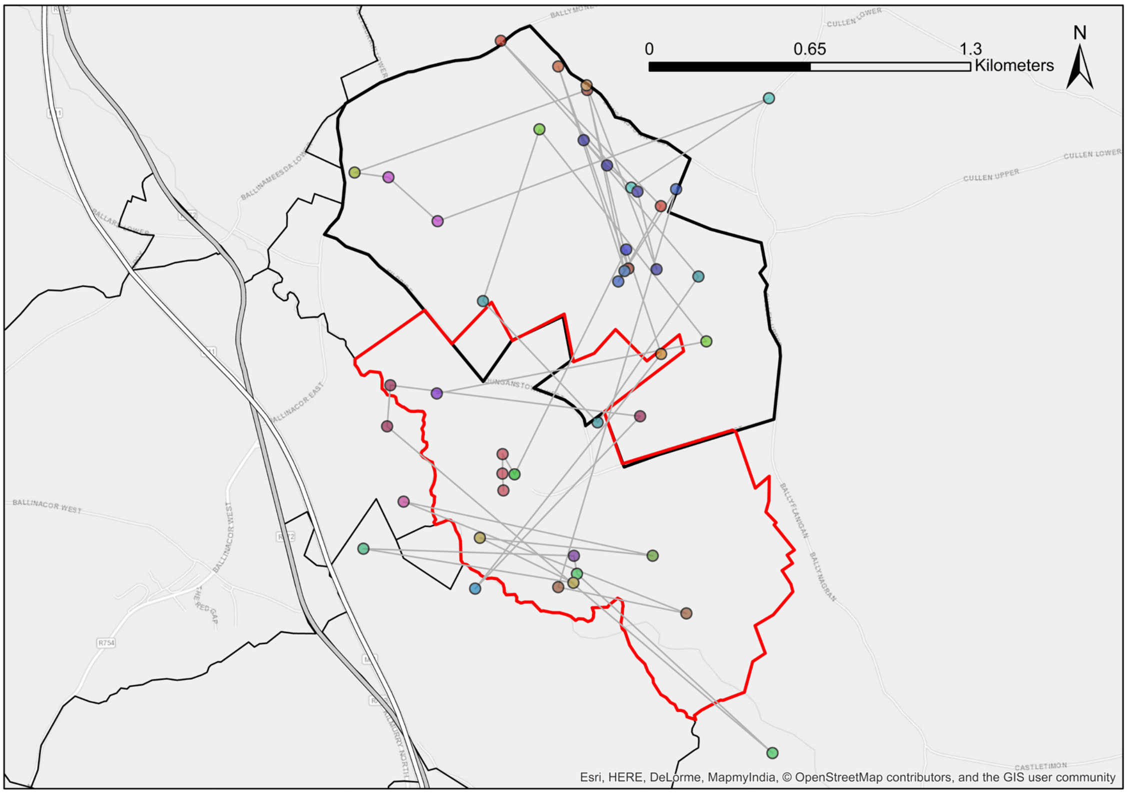

Figure 2.7 M01's dispersal trajectory. January and February 2011. M01 moved overnight (13/02/11) to the adjacent social group to the north. This move was preceded by two extra-territorial excursions to the new social group, and he also returned to his natal group twice in February. In these two months he travelled 31 km over an area of 2.4 km^2. During this time period he made extra-territorial excursions to approximately four other social groups.

Filled circles represent GPS locations, with different colours indicating different nights. Thin grey lines join consecutive GPS locations. The thick red line outlines the natal territory boundary, the thick black line outlines new territory boundary, and thin black lines outline other territory boundaries. The average distance between main setts in the study area was 1.3 km^2.

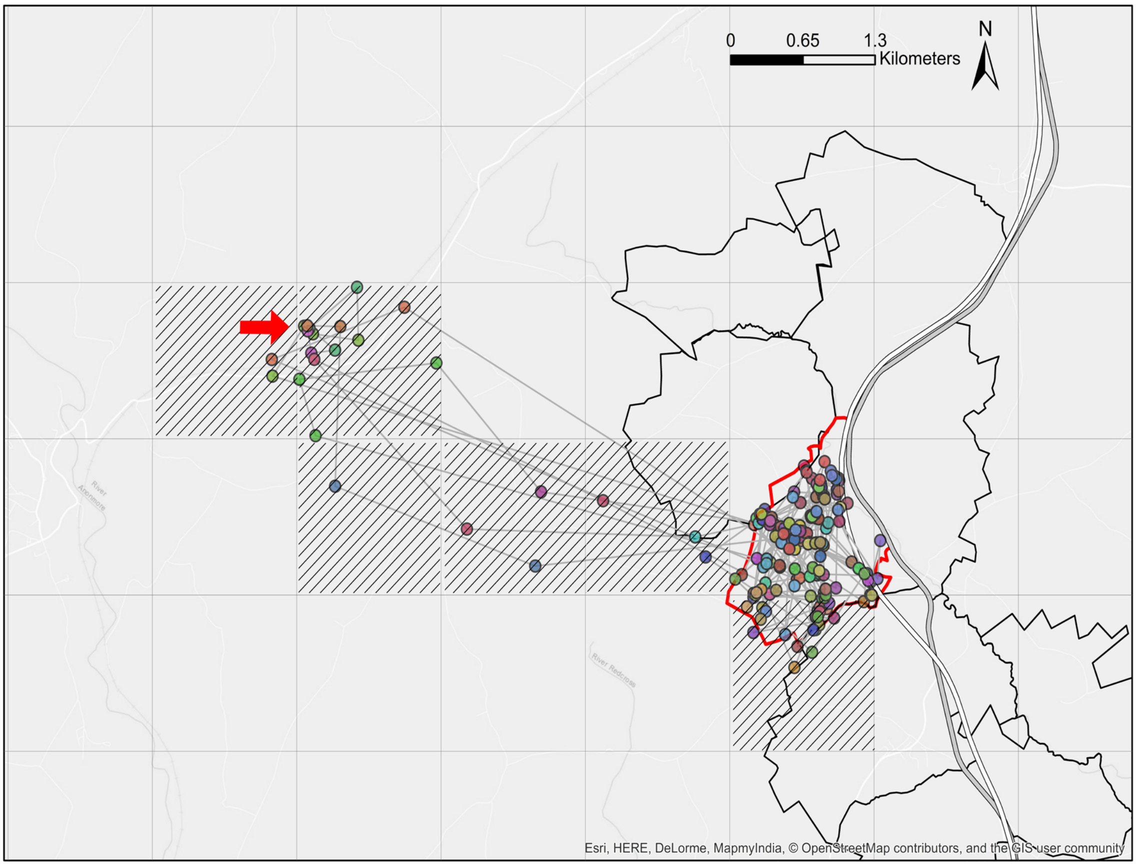

Figure 2.8 Trajectory of M02's exploratory journeys. June and July 2013. Exploratory forays preceding dispersal (event missed) to a non-adjacent social group to the north-west. Extra-territorial excursions were made over multiple nights where he did not return to the natal social group but spent the day in the sett(s) of other social groups. During this time, M02 travelled 111 km through an area of approximately 5 km^2, passing through approximately 6 social groups. These forays did not result in dispersal at the time. However, M02 was not trapped in his natal group at the following trapping session, and was found dead in the area he had visited in 2014.

Filled circles represent GPS locations, with different colours indicating different nights. Thin grey lines join consecutive GPS locations. The thick red line outlines the natal territory boundary and thin black lines outline other territory boundaries. 1.3 km is the average distance between main setts in the study area. A grid of 1.3 km^2 is used as a proxy for social group territories outside of the study area. The red arrow indicates where he was found dead.

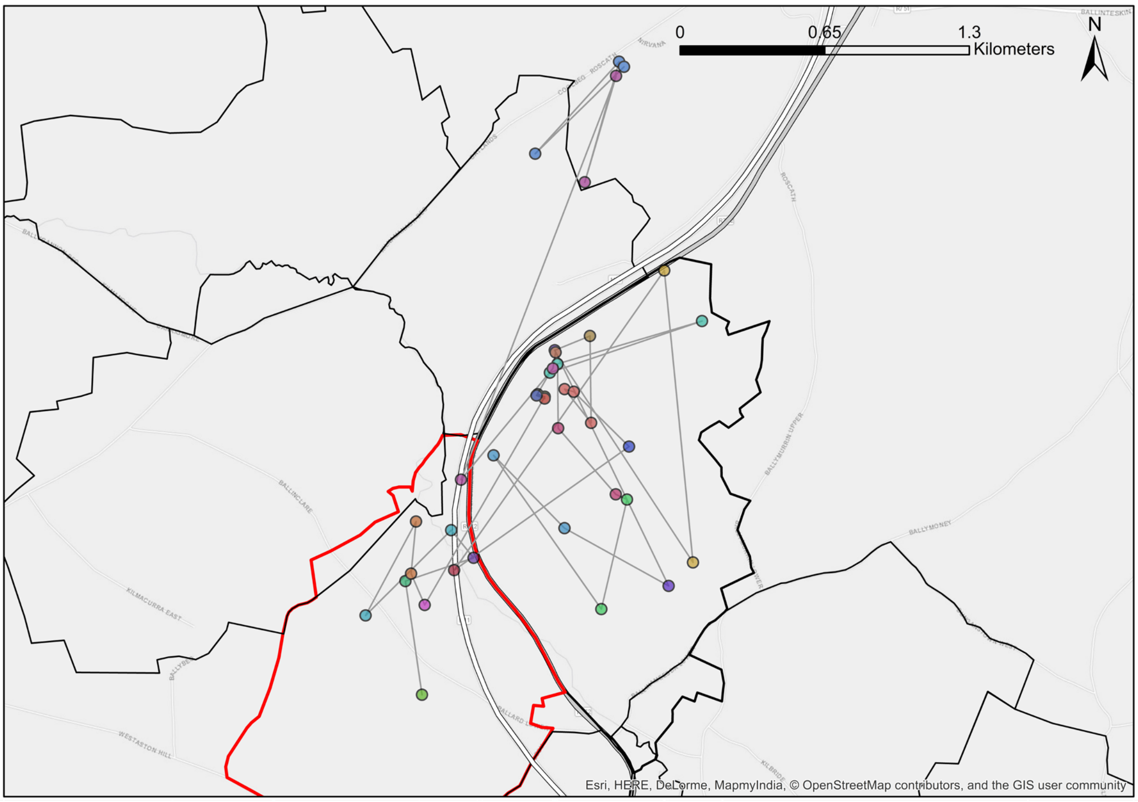

Figure 2.9 M04's dispersal trajectory. February 2013. M04 dispersed overnight on the 10th of February, making two excursions back to his natal group. Extra-territorial excursions were also made to the north of his new group. During this time, he travelled 20.5 km through an area of approximately 2 km^2, visiting approximately additional 2 social groups.

Filled circles represent GPS locations, with different colours indicating different nights. Thin grey lines join consecutive GPS locations. The thick red line outlines the natal territory boundary, the thick black line represents the new territory boundary, and thin black lines outline other territory boundaries. The average distance between main setts in the study area was 1.3 km^2.

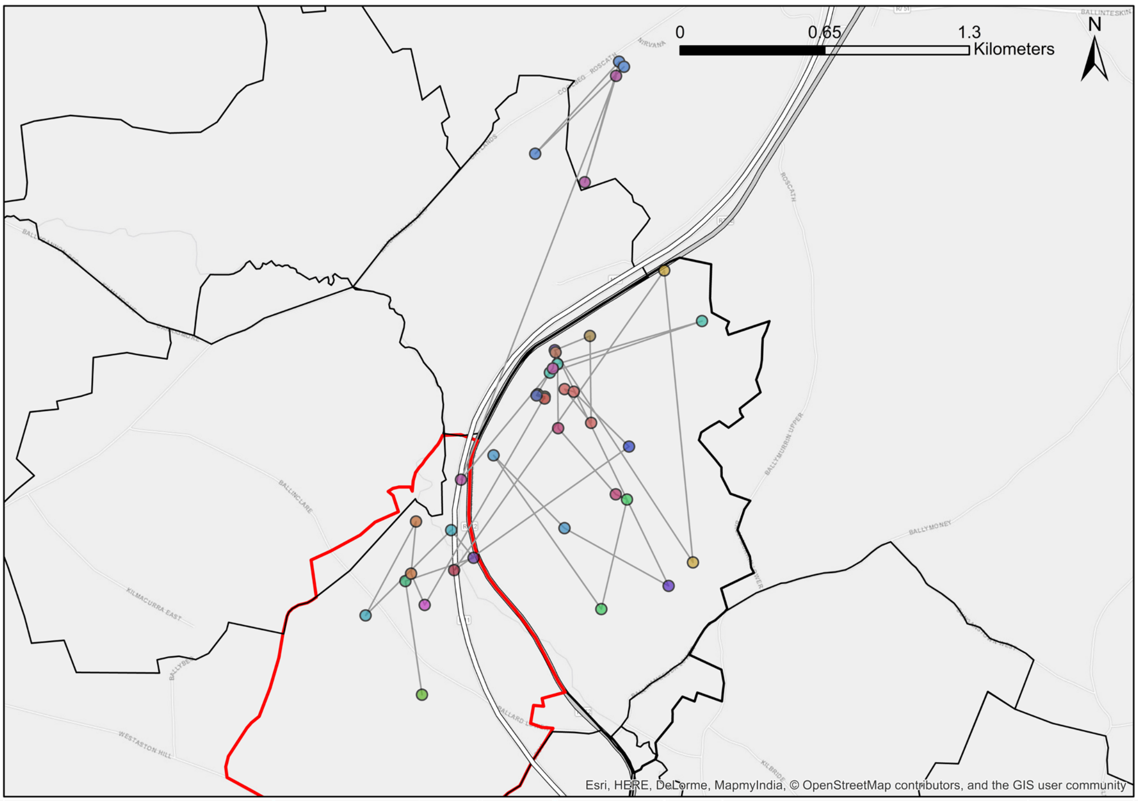

Figure 2.10 M07's dispersal trajectory. January to April 2015. Extra-territorial excursions were made on single nights in January and February. In February M07 spent five days in the new social group before returning to his natal group. He moved to the new social group on the 21st of February, making eight visits back to his natal territory during next three months, including one overnight stay. During this time, M07 travelled 210 km through an area of approximately 4 km^2, visiting approximately additional 6 social groups.

Filled circles represent GPS locations, with different colours indicating different nights. Thin grey lines join consecutive GPS locations. The thick red line outlines the natal territory boundary, the thick black line represents the new territory boundary, and thin black lines outline other territory boundaries. The average distance between main setts in the study area was 1.3 km^2. A grid of 1.3 km^2 is used as a proxy for social group territories outside of the study area.

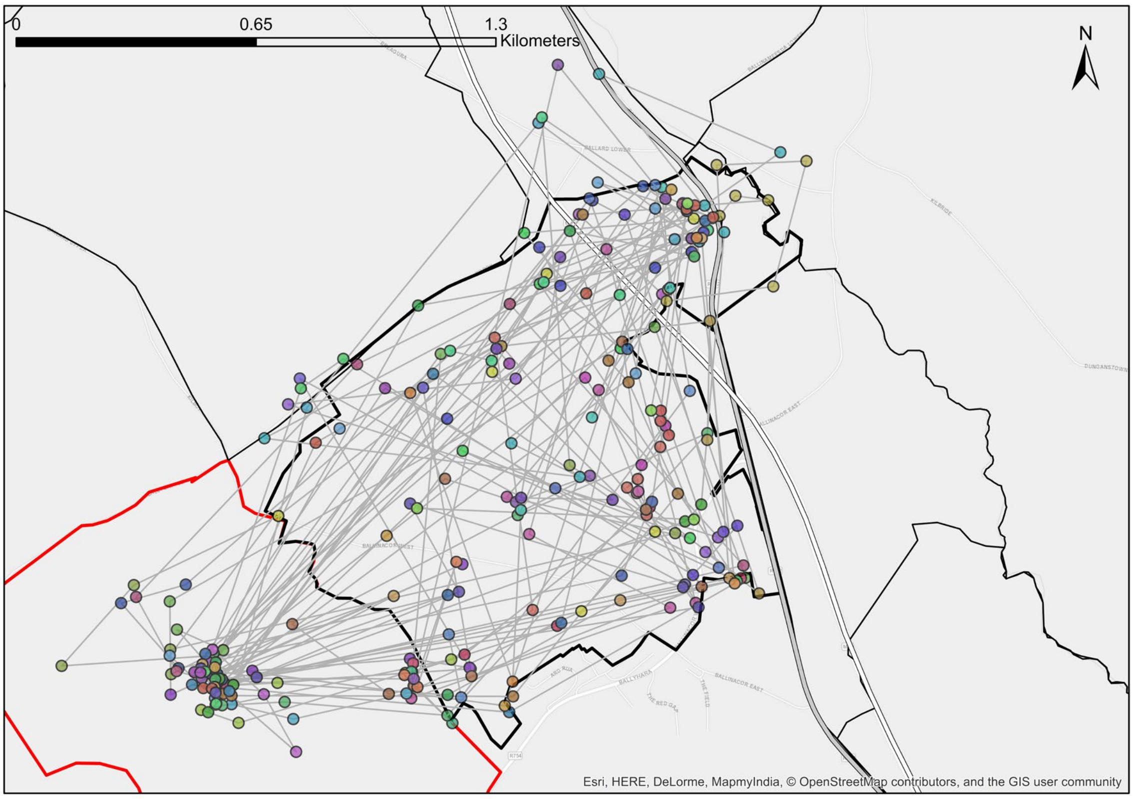

Figure 2.11 M08's dispersal trajectory. GPS fixes April to June 2015. Gradual move to the adjacent social group to the north-east. Initially, frequent extra-territorial excursions were made over single nights and M08 returned to the natal social group. He progressed to spending the day in the neighbouring sett, and gradually spent more time in the new social group and less in his natal group, before finally ceasing to return to the natal social group altogether. In these two months M08 travelled 138 km through an area of 1.8 km^2.

Filled circles represent GPS locations, with different colours indicating different nights. Thin grey lines join consecutive GPS locations. The thick red line outlines the natal territory boundary, the thick black line outlines new territory boundary, and thin black lines outline other territory boundaries. The average distance between main setts in the study area was 1.3 km^2.

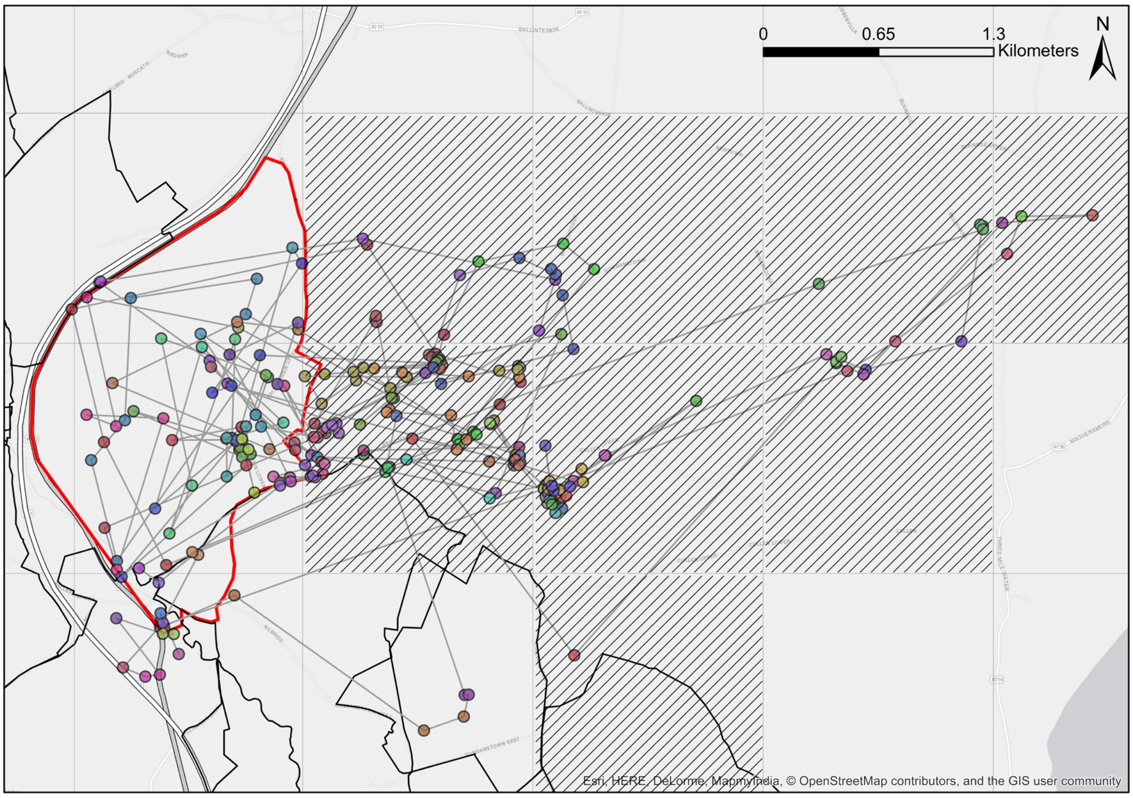

Figure 2.12 M09's trajectory. June to August 2016. M09 was an immigrant into the study area. He was collared in The Bracken group's territory, but it quickly became clear that he was not a resident there. In this period, he travelled 110 km through an area of approximately 8.5 km^2, passing through approximately 11 social groups' territories before we lost contact. This is an underestimate as his collar was faulty and functioned intermittently, before failing.

Filled circles represent GPS locations, with different colours indicating different nights. Thin grey lines join consecutive GPS locations. The thick red line outlines the territory boundary in which he was trapped. Thin black lines outline other territory boundaries. The average distance between main setts in the study area was 1.3 km^2. A grid of 1.3 km^2 is used as a proxy for social group territories outside of the study area. Quadrants with GPS locations are highlighted using a hatched pattern.

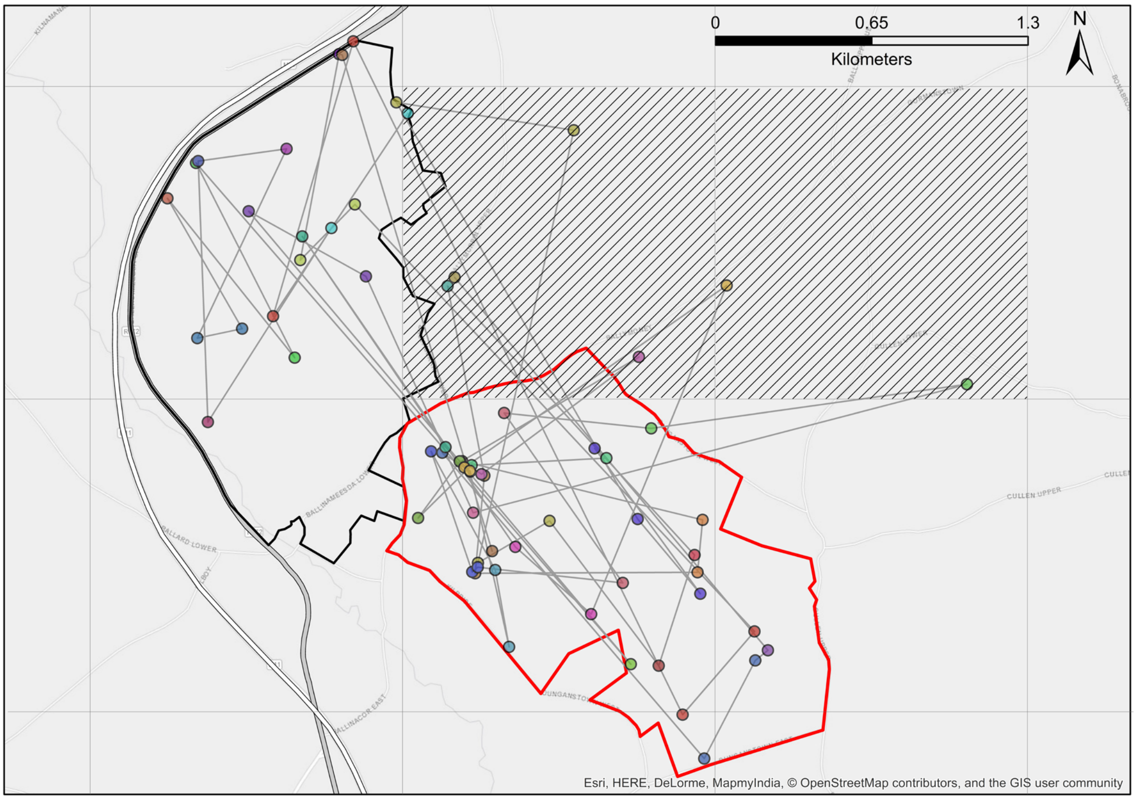

Figure 2.13 M13's dispersal trajectory. January and February 2011. In these two months he travelled 59 km through an area of approximately 4 km^2, and visited approximately two other social groups.

Filled circles represent GPS locations, with different colours indicating different nights. Thin grey lines join consecutive GPS locations. The thick red line outlines the natal territory boundary, the thick black line outlines new territory boundary, and thin black lines outline other territory boundaries. The hatched area represents the part of the natal territory that was lost. The average distance between main setts in the study area was 1.3 km^2.

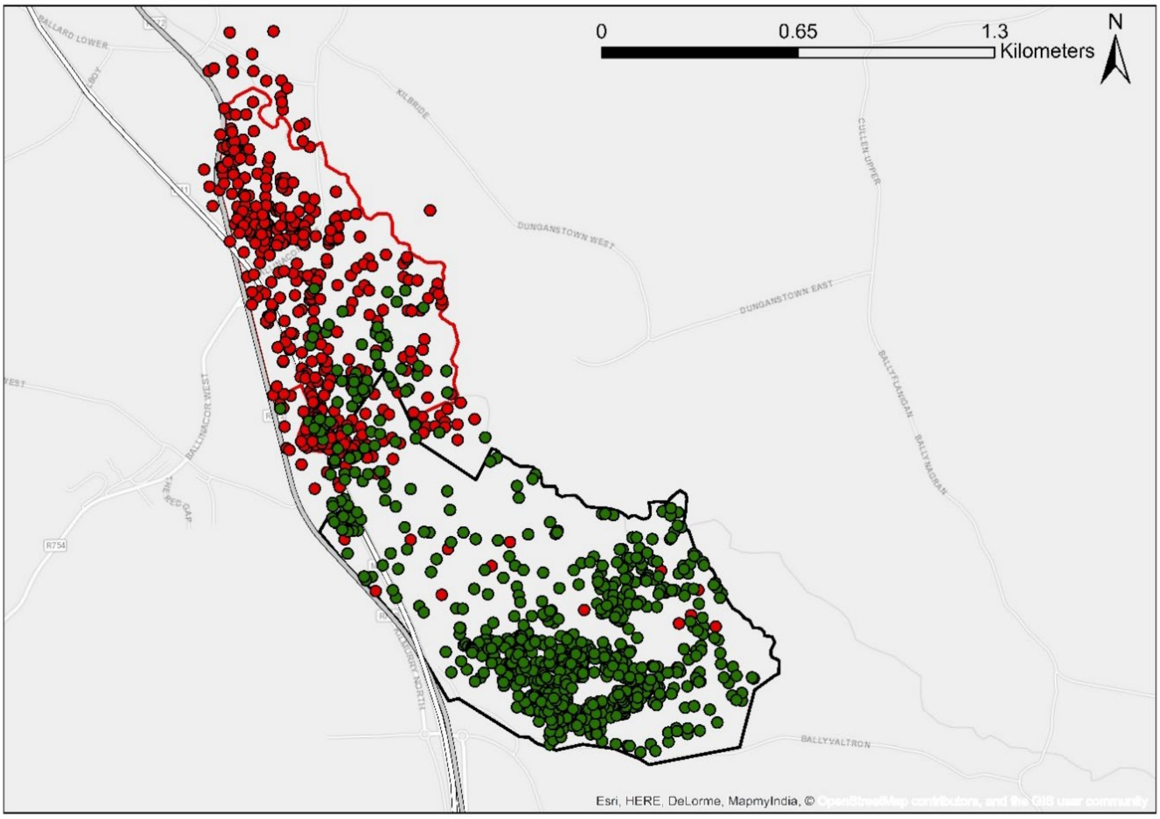

Figure 2.14 F05's pre-and post-dispersal territories. F05 was not wearing a collar when she dispersed but was before and afterwards. The filled red circles represent her GPS locations before dispersal (2010) and the green filled circles represent her GPS locations after dispersal (2011). The average distance between main setts in the study area was 1.3 km^2.

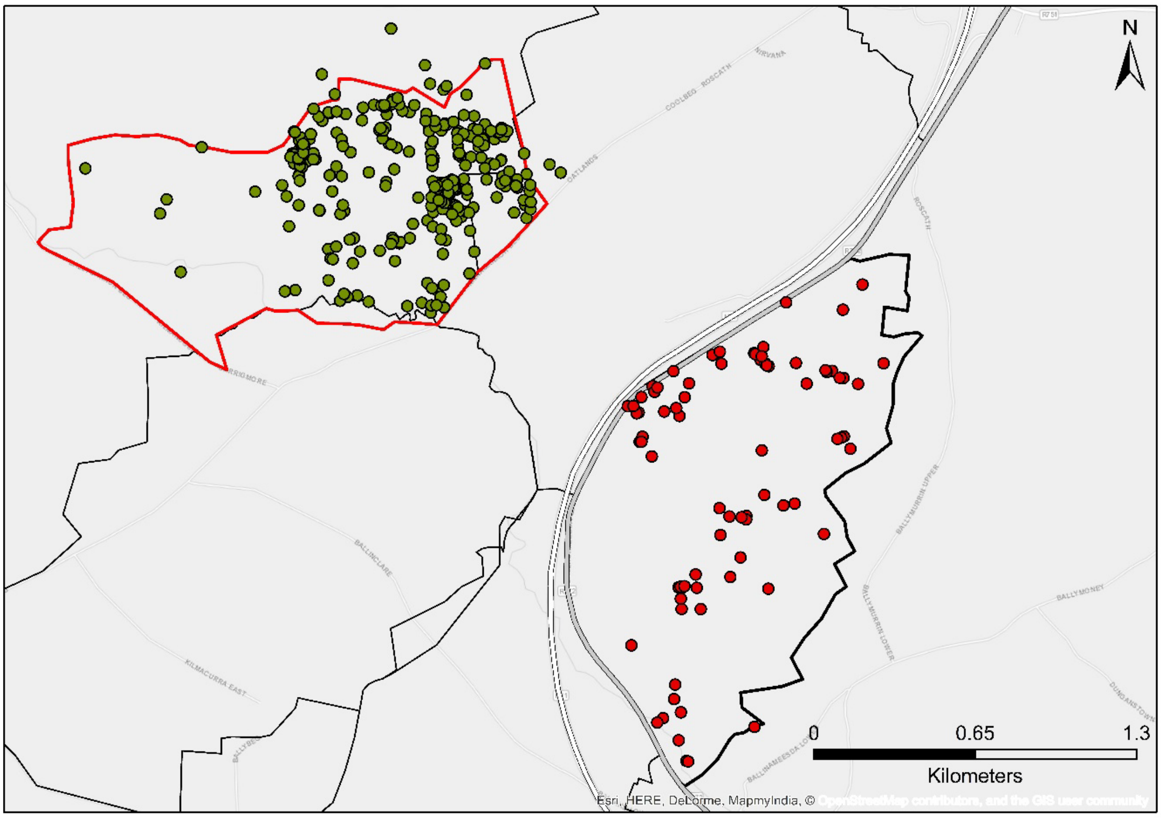

Figure 2.15 F06's pre-and post-dispersal territories. F06 was not wearing a collar when she dispersed but was before and afterwards. The filled green circles represent her GPS locations before dispersal (2010) and the red filled circles represent her GPS locations after dispersal (2011). The average distance between main setts in the study area was 1.3km^2.

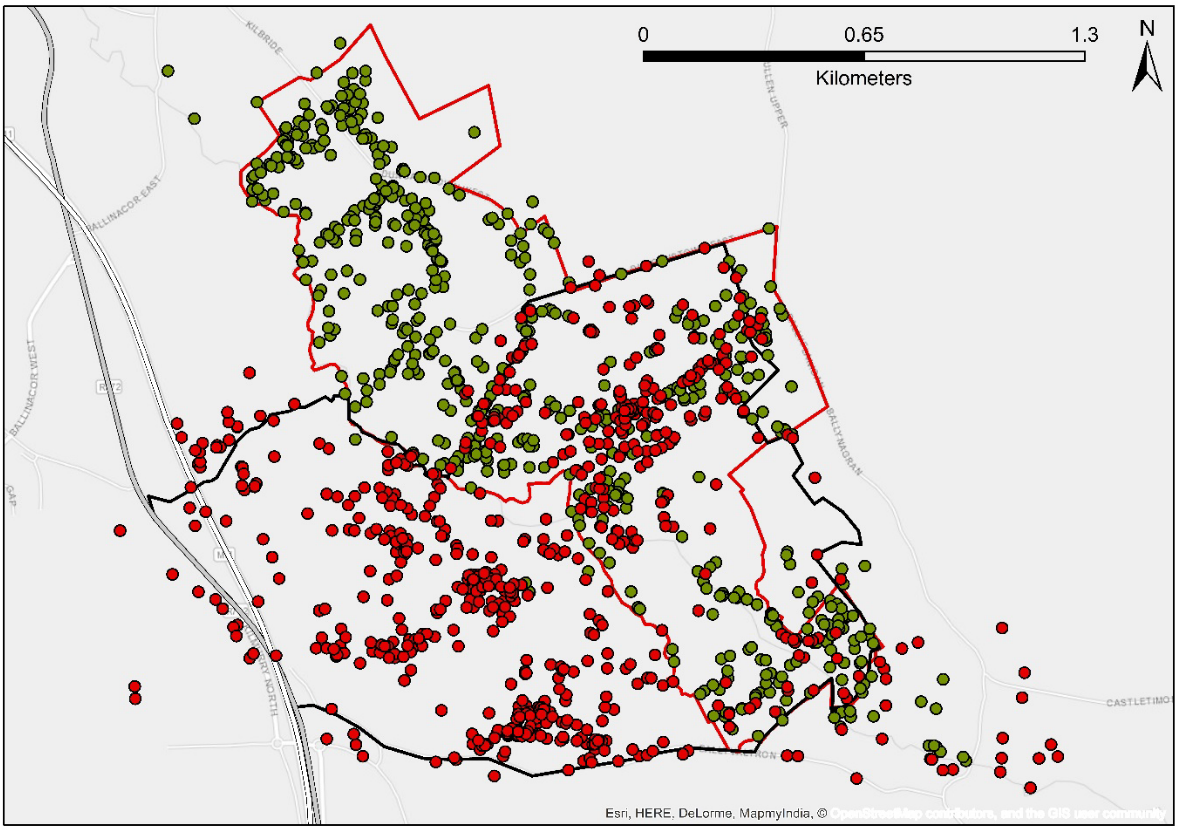

Figure 2.16 M10's pre-and post-dispersal territories. M10 was not wearing a collar when he dispersed but was before and afterwards. The filled green circles represent his GPS locations before dispersal (2010) and the red filled circles represent his GPS locations after dispersal (2014). His natal territory underwent fission in 2011 and he retained the southern portion, while expanding his range into adjacent territories (Gaughran et al. 2018). The average distance between main setts in the study area was 1.3 km^2.

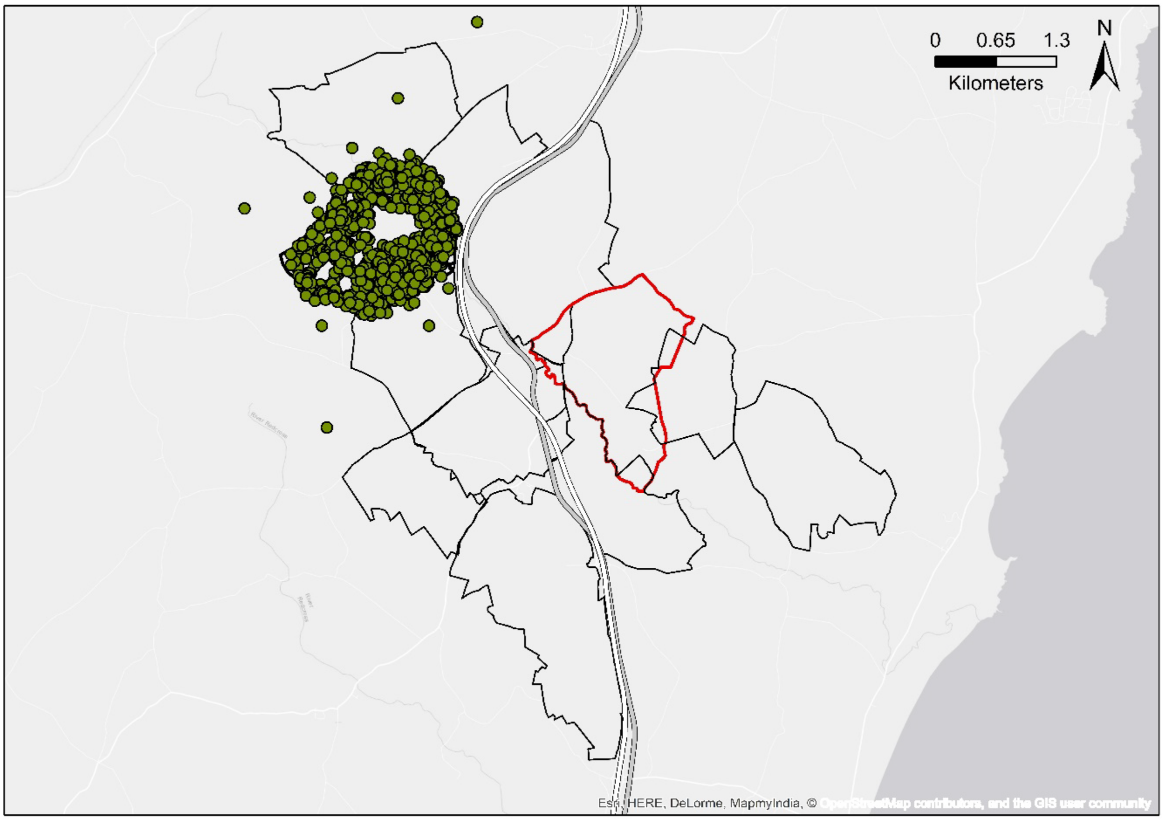

Figure 2.17 F01's pre-and post-dispersal territories. Based on genetic maternal assignment, it was discovered that F01, who was only ever trapped and collared in The Quarry territory, had been born to the resident female in Ballard. She was a young adult when first trapped. Filled green circles represent her GPS locations in The Quarry Territory. The thick red line outlines her natal territory boundary, Ballard. Thin black lines outline other territory boundaries. The average distance between main setts in the study area was 1.3 km^2.

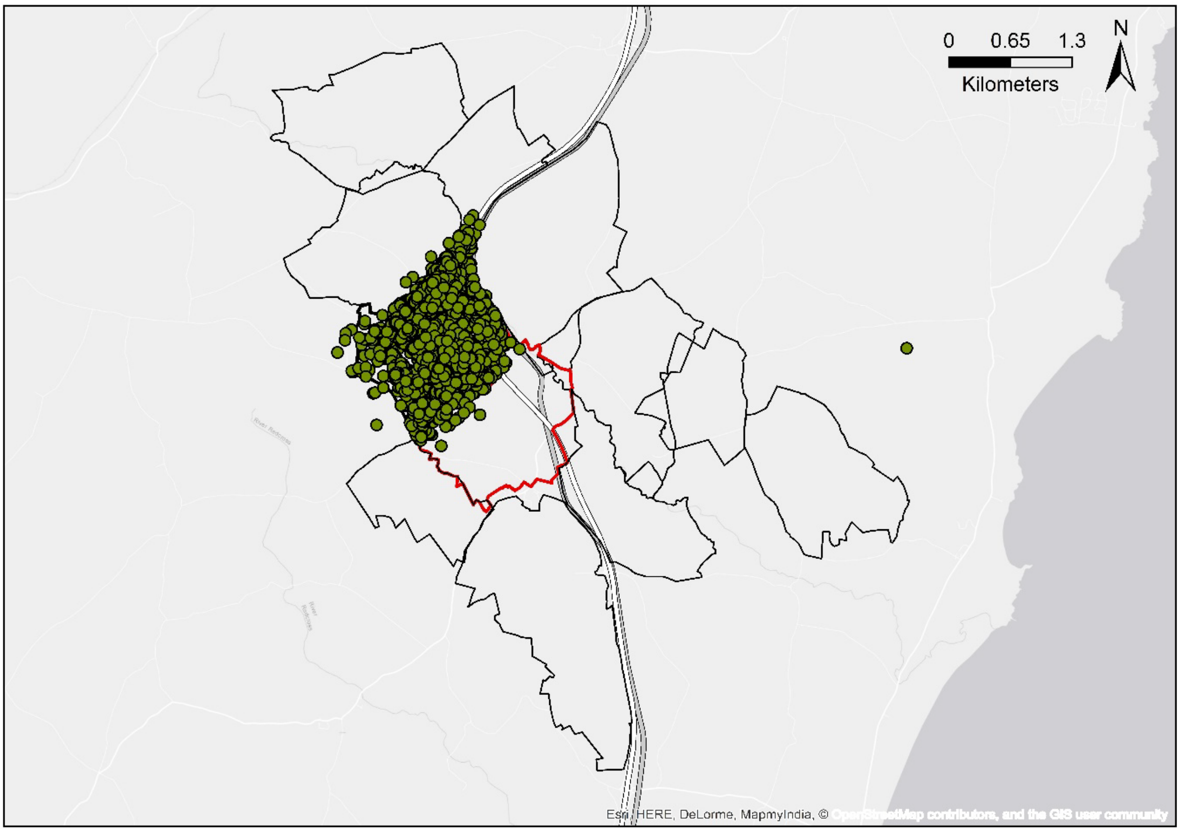

Figure 2.18 F02's pre-and post-dispersal territories. F02 was trapped and collared every year, except for the final year of the study, in The Oak social group's territory. Filled green circles represent her GPS locations. In the final few trapping session (when collars were being removed) we failed to trap F02 in her original group, and only ever trapped her at the main sett of the adjacent social group, The Driving Range (outlined by a thick red line). Thin black lines outline other territory boundaries. The average distance between main setts in the study area was 1.3 km^2.

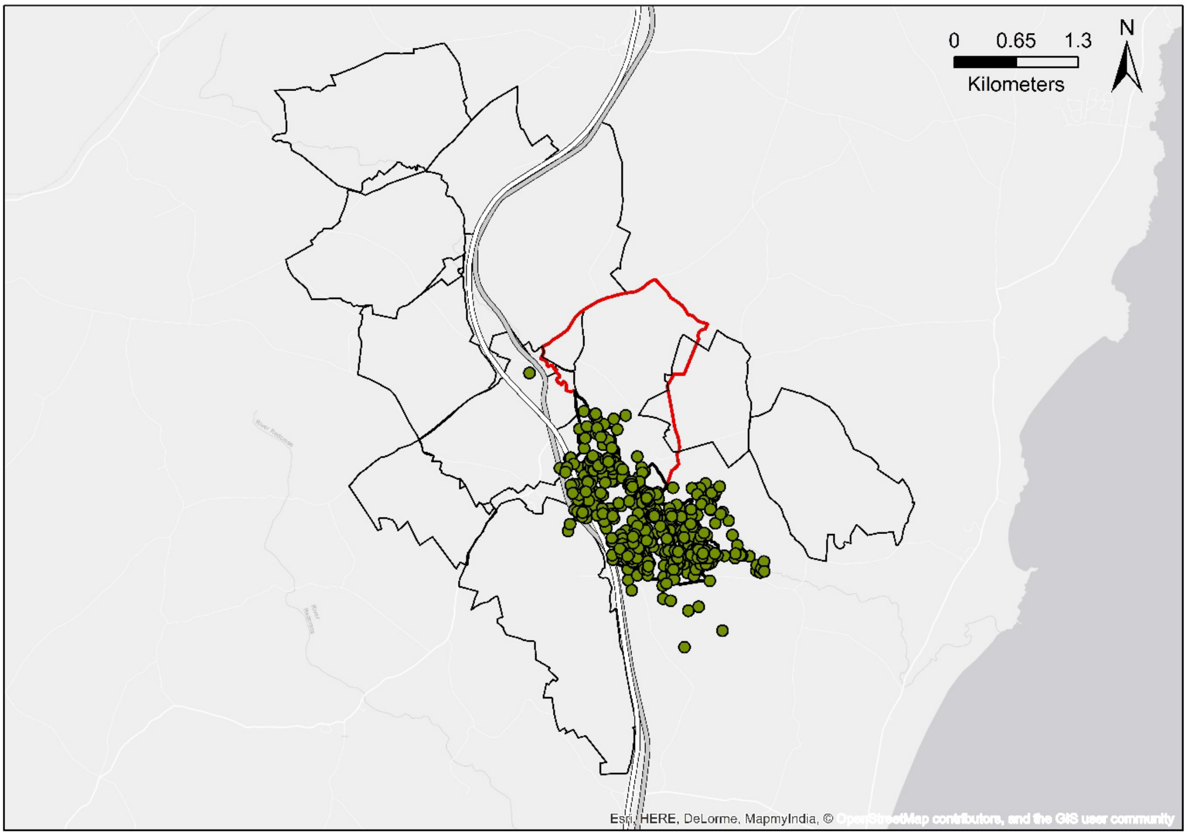

Figure 2.19 M06's pre-and post-dispersal territories. M06 was only trapped and collared consistently in The Briars social group's territory. Filled green circles represent his GPS locations. In the final few trapping session (when collars were being removed) we failed to trap him in his original territory, and only ever trapped him at the main sett of the adjacent social group, Bluebell Woods (outlined by a thick red line). Thin black lines outline other territory boundaries. The average distance between main setts in the study area was 1.3 km^2.

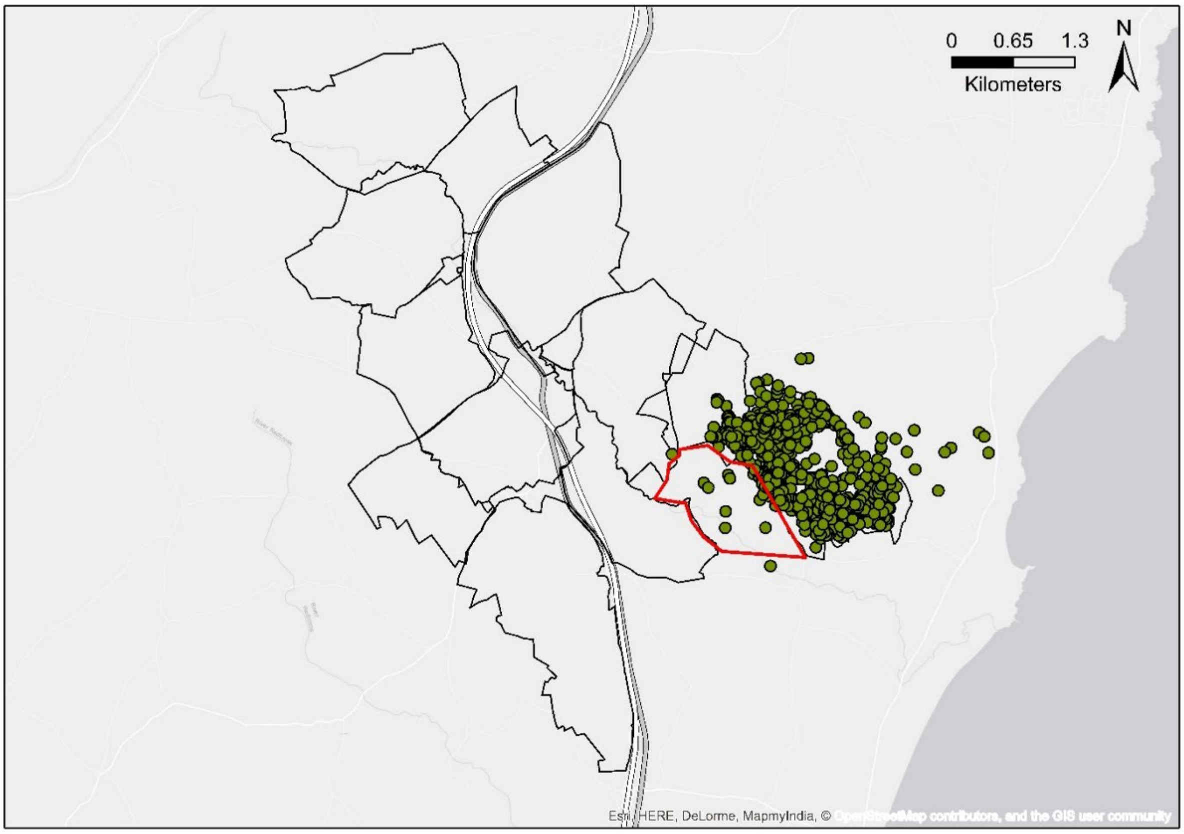

Figure 2.20 M11's pre-and post-dispersal territories. M11 was only trapped and collared in The Briars social group's territory. Filled green circles represent his GPS locations. In the final few trapping session (when collars were being removed) we failed to trap M11 in his original group, and only ever trapped him at the main sett of the adjacent social group, The Lane (outlined by a thick red line). Thin black lines outline other territory boundaries. The average distance between main setts in the study area was 1.3 km^2.
