# Supplementary material for: Dispersal patterns in a medium‐density Irish badger population: Implications for understanding the dynamics of tuberculosis transmission
Source: Ecol Evol. 2019 Nov 13;9(23):13142–52. doi: 10.1002/ece3.5753 (PMC6912907; doi:10.1002/ece3.5753)
Supplement: Supplementary file 3 [file ECE3-9-13142-s003.docx]

**SI Table** **1. Summary statistics for distanced moved (km) by dispersers.** Distance moved, measured as the straight-line distance between centroids of natal and new social groups. Where new social group boundaries were unknown, distance was estimated based on centroid of GPS records of the dispersed badger. Immigrants (N=2) not included as location of natal territory was unknown.

| **Group** | **Mean (km)** | **SD ±** | **Median (km)** | **Max** | **Min** | **No. obvs.** |
| --- | --- | --- | --- | --- | --- | --- |
| All badgers | 2.3 | 2.3 | 1.4 | 10.5 | 0.8 | 25 |
| All yearlings | 4.4 | 3.8 | 3.1 | 10.5 | 1.4 | 6 |
| All younger adults | 1.6 | 1.2 | 1.2 | 4.7 | 0.8 | 15 |
| All older adults | 1.4 | 0.4 | 1.3 | 2.1 | 1.2 | 4 |
| All females (♀) | 2.8 | 2.8 | 1.5 | 10.5 | 0.8 | 14 |
| All ♀ yearlings | 5.9 | 3.9 | 5.4 | 10.5 | 2.2 | 4 |
| All ♀ younger adults | 1.7 | 1.1 | 1.3 | 4.2 | 0.8 | 7 |
| All ♀ older adults | 1.5 | 0.5 | 1.3 | 2.1 | 1.2 | 3 |
| All males (♂) | 1.5 | 1.1 | 1.2 | 4.7 | 0.8 | 11 |
| All ♂ yearlings | 1.4 | 0.0 | 1.4 | 1.4 | 1.4 | 2 |
| All ♂ younger adults | 1.6 | 1.3 | 1.1 | 4.7 | 0.8 | 8 |
| All ♂ older adults | 1.2 | NA | 1.2 | 1.2 | 1.2 | 1 |
