# Supplementary material for: Dispersal patterns in a medium‐density Irish badger population: Implications for understanding the dynamics of tuberculosis transmission
Source: Ecol Evol. 2019 Nov 13;9(23):13142–52. doi: 10.1002/ece3.5753 (PMC6912907; doi:10.1002/ece3.5753)
Supplement: Supplementary file 4 [file ECE3-9-13142-s004.docx]

**SI Table 2.** **Summary statistics for the number of social groups crossed by dispersers.** Zero indicates move to adjacent social group. Where new group boundaries were unknown, the number was extrapolated based on mean distance between main setts within study area (1313m).

| **Group** | **Mean** | **SD ±** | **Median** | **Max** | **Min** | **No. obvs** |
| --- | --- | --- | --- | --- | --- | --- |
| all badgers | 1 | 1.8 | 0 | 7 | 0 | 25 |
| all yearlings | 2.7 | 2.9 | 3 | 7 | 0 | 6 |
| all younger adults | 0.5 | 0.9 | 0 | 3 | 0 | 15 |
| all older adults | 0.8 | 1.5 | 0 | 3 | 0 | 4 |
| all females (♀) | 1.6 | 2.2 | 1 | 7 | 0 | 14 |
| all ♀ yearlings | 4 | 2.6 | 4 | 7 | 1 | 4 |
| all ♀ younger adults | 0.6 | 0.8 | 0 | 2 | 0 | 7 |
| all ♀ older adults | 1 | 1.7 | 0 | 3 | 0 | 3 |
| all males (♂) | 0.3 | 0.9 | 0 | 3 | 0 | 11 |
| all ♂ yearlings | 0 | 0 | 0 | 0 | 0 | 2 |
| all ♂ younger adults | 0.4 | 1.1 | 0 | 3 | 0 | 8 |
| all ♂ older adults | 0 | NA | 0 | 0 | 0 | 1 |
