# Supplementary material for: Dispersal patterns in a medium‐density Irish badger population: Implications for understanding the dynamics of tuberculosis transmission
Source: Ecol Evol. 2019 Nov 13;9(23):13142–52. doi: 10.1002/ece3.5753 (PMC6912907; doi:10.1002/ece3.5753)
Supplement: Supplementary file 5 [file ECE3-9-13142-s005.docx]

**SI Text 1. Summary of genetic analyses results for N11 badgers.**

DNA samples collected during fieldwork were sequenced by Eurofins Genomics GmbH. I used the computer programs CERVUS (Kalinowski et al. 2007) and COLONY (Wang and Santure 2009) to perform sibship and parentage analyses on the data. In CERVUS, we were able to assign a Sampled parent (either a mother or a father) at >85% confidence levels to 47.7% of candidate offspring (41 of 86, Table A). **Both parents** were assigned to 34.9% of candidate offspring (30 of 86). Sampled **mothers** were assigned to 44.2% of candidate offspring (38 of 86). Sampled **fathers** were assigned to 38.4% of candidate offspring (33 of 86). In one case, COLONY rejected a maternity assigned by CERVUS (F13 as F14’s mother), and in another case, it assigned a paternity not assigned by CERVUS (M14 as F15’s father). These revisions were applied to sibship analysis in COLONY. 52.3% of candidate offspring (45 of 86) had no Sampled parents assigned in CERVUS. However, in COLONY inferred parentage was assigned to 4 additional individuals (F16, M15, F17 and M08, Table A) based on COLONY's ability to detect sibship relationships even when parents are not Sampled.  Of the 14 females who were assigned offspring, the mean number of offspring over the course of the study was 2.6 (range 1–7) (Table B). Of the 16 males who were assigned offspring, the mean no. of offspring over the course of the study was 1.9 (range 1–5) (Table C). In COLONY 14 **full sibling** (FS) dyads were assigned but only 10 of these were assigned at a >85% confidence levels (Table D). A total of 252 **half sibling** (HS) dyads were assigned by COLONY with 76 of these at <85% confidence levels (Table E), including one instance when both parents were unsampled but inferred.

Our analysis in COLONY identified 23 cases of **extra-group paternity** (70% of all assigned paternities). The majority (59%) of EGPs were from adjacent social groups. There were 13 cases of **multiple paternity** within the same litter (40% of all assigned paternities). According to trapping records from Spring trapping session, there were 12 cases of **plural breeding** within social groups in total (Table F). On average, there were 1.6 instances of plural breeding per year (range 0–3). F18 and F02 from The Oak social group were observed to be lactating simultaneously on four different years, F19 and F20 (The Big Tree social group) on three different years, and both F21 and F22 (The Pines social group) and F23 and F24 (Ballad SG) on two different years. Field observations were confirmed by DNA analysis in the case of F18 and F02 in 2015. However, for the other observations a cub was not assigned by DNA analysis to *both* of the females in question in the relevant year, and plural breeding could not be confirmed through genetic analysis.

**Dispersal events** may have been missed if a badger was not wearing a GPS collar, or if it had already dispersed by the time it was first trapped. Dispersal was inferred for four badgers through assignment of maternity and confirmed for one badger that had trapping and partial GPS records that suggested dispersal (Table G).

**Table A. Results of Parentage Analysis performed in CERVUS (and COLONY where indicated)**

| **Offspring ID** | **YOB** | **Natal Social Group~** | **Assigned Maternity** | **Assigned Paternity** |
| --- | --- | --- | --- | --- |
| F01 | 2013 | The Quarry | F23* | M04* |
| M01 | 2009 | Bluebell Woods | NA | M17 |
| M16^ | 2011 | Bluebell Woods | F03^ | M10 |
| M18 | 2014 | Driving Range | F25 | M17 |
| F03 | 2009 | Bluebell Woods | NA | M19 |
| M20 | 2013 | Hawthorn | F14 | M01 |
| F04 | 2015 | The Briars | F23 | NA |
| F16 | 2014 | Oak | F02 | UNKWNM1** |
| M21 | 2016 | JBs | F28 | M08 |
| F26 | 2015 | Oak | F18 | M16 |
| F27 | 2015 | Ballad | F23* | M10* |
| F29 | 2010 | Hawthorn | F13 | M14 |
| F30 | 2014 | Ballad | F23* | M16* |
| M15 | 2014 | Oak | F02 | UNKWNM1** |
| F14 | 2011 | Hawthorn | F13*** | M22 |
| M23 | 2015 | Oak | F18 | M16 |
| M05 | 2008 | Cemetery | F31 | NA |
| F17 | 2013 | The Orchard | UNKWNF1** | M24 |
| F32 | 2011 | Sycamore | F34* | M24* |
| F33 | 2012 | Oak | F02* | M24* |
| F07 | 2014 | Oak | F02 | NA |
| M25 | 2011 | The Pines | F06* | M26* |
| M08 | 2013 | The Orchard | UNKWNF1** | M24 |
| F08^ | 2015 | Cemetery | F03^ | M16^ |
| F15 | 2009 | Hawthorn | F13 | M14*** |
| M27 | 2016 | JBs | F28 | NA |
| M29 | 2014 | Ballad | F23* | M16* |
| F09 | 2011 | Sycamore | F34* | M24* |
| F10 | 2014 | Oak | F02 | NA |
| M28 | 2010 | Bluebell Woods | F35* | M26* |
| F36 | 2011 | Oak | F18* | M05* |
| F11 | 2013 | Ballad | F23 | M30 |
| M31 | 2015 | Oak | F02 | M32 |
| M17 | 2007 | Cemetery | F31 | NA |
| F37 | 2014 | Cemetery | F03 | M10 |
| M33 | 2015 | Big Tree | F19 | M34 |
| M11 | 2013 | Big Tree | F19 | M04 |
| F23 | 2011 | Bluebell Woods | F35 | NA |
| M35 | 2015 | Big Tree | F19* | M34* |
| F12 | 2015 | Ballad | F23* | M16* |
| F38 | 2012 | Hawthorn | F15 | M14*** |

~ Where badger not first trapped as a cub/yearling, natal group is based on GPS tracking/ trapping data

* Assigned at >95% confidence in CERVUS (all others assigned at >85% confidence level)

** Parentage assigned in COLONY but not CERVUS

*** Parentage assigned in CERVUS but rejected in COLONY

^ Inbreeding

**Table B.** **Number of offspring per mother over the course of the study 2010–2016**

| **Mother** | **No.** | | **OS1** | | **OS2** | | **OS3** | | **OS4** | | **OS5** | | **OS6** | | **OS7** |  |  |
| --- | --- | --- | --- | --- | --- | --- | --- | --- | --- | --- | --- | --- | --- | --- | --- | --- | --- |
| F02 | 6 | | F16 | | M15 | | F33 | | F07 | | F10 | | M31 | |  |  |  |
| F03 | 3 | | M16 | | F08 | | F37 | |  | |  | |  | |  |  |  |
| F25 | 1 | | M18 | |  | |  | |  | |  | |  | |  |  |  |
| F06 | 2 | | M25 | | KB RTA F | |  | |  | |  | |  | |  |  |  |
| F13 | 2 | | F29 | | F15 | |  | |  | |  | |  | |  |  |  |
| F28 | 2 | | M21 | | M27 | |  | |  | |  | |  | |  |  |  |
| F14 | 1 | | M20 | |  | |  | |  | |  | |  | |  |  |  |
| F31 | 2 | | M05 | | M17 | |  | |  | |  | |  | |  |  |  |
| F35 | 2 | | M28 | | F23 | |  | |  | |  | |  | |  |  |  |
| F34 | 2 | | F32 | | F09 | |  | |  | |  | |  | |  |  |  |
| F15 | 1 | | F38 | |  | |  | |  | |  | |  | |  |  |  |
| F18 | 2 | | F26 | | F36 | |  | |  | |  | |  | |  |  |  |
| F23 | 7 | | F01 | | F04 | | F27 | | F30 | | M29 | | F11 | | F12 |  |  |
| F19 | 3 | | M33 | | M11 | | M35 | |  | |  | |  | |  |  |  |
|  | |  | |  | |  | |  | |  | |  | |  | | |  |

**Table C.** **Number of offspring per father over the course of the study 2010–2016. Super-rangers are marked with an ***

| **Father** | **No.** | **OS1** | **OS2** | **OS3** | **OS4** | **OS5** |
| --- | --- | --- | --- | --- | --- | --- |
| M01 | 1 | M20 |  |  |  |  |
| M16 | 5 | F26 | F30 | F08 | M29 | F12 |
| M14 | 2 | F29 | F15 |  |  |  |
| M22* | 1 | F14 |  |  |  |  |
| M26* | 2 | M25 | M28 |  |  |  |
| M04* | 2 | F01 | M11 |  |  |  |
| M30 | 1 | F11 |  |  |  |  |
| M05 | 1 | F36 |  |  |  |  |
| M08 | 1 | F28 |  |  |  |  |
| M36 | 1 | F25 |  |  |  |  |
| M34* | 2 | M33 | M35 |  |  |  |
| M10* | 3 | M16 | F27 | F37 |  |  |
| M19 | 1 | F03 |  |  |  |  |
| M17 | 2 | M01 | M18 |  |  |  |
| M31 | 1 | M32 |  |  |  |  |
| M24* | 5 | F17 | F32 | F33 | M08 | F09 |

**Table D. Full sibships identified in COLONY at > 85% confidence levels.**

| **Offspring #1** | **Offspring #2** | **Probability (>0.85)** |
| --- | --- | --- |
| M16 | F37 | 1 |
| F16 | M15 | 1 |
| F26 | M23 | 1 |
| F29 | F15 | 1 |
| F30 | M29 | 1 |
| F30 | F12 | 1 |
| F17 | M08 | 1 |
| F32 | F09 | 1 |
| M29 | F12 | 1 |
| M33 | M35 | 1 |
| F39 | M34 | 0.87 |

**Table E. Half sibships identified in COLONY at > 85% confidence levels.**

| **Offspring1** | **Offspring2** | **Probability (>0.85)** |
| --- | --- | --- |
| F01 | F04 | 1 |
| F01 | F27 | 1 |
| F01 | F30 | 1 |
| F01 | M29 | 1 |
| F01 | F11 | 1 |
| F01 | M11 | 1 |
| F01 | F12 | 1 |
| M01 | M18 | 1 |
| M16 | F27 | 1 |
| M16 | F08 | 1 |
| F04 | F27 | 1 |
| F04 | F30 | 1 |
| F04 | M29 | 1 |
| F04 | F11 | 1 |
| F04 | F12 | 1 |
| F16 | F33 | 1 |
| F16 | F07 | 1 |
| F16 | F10 | 1 |
| F16 | M31 | 1 |
| M21 | M27 | 1 |
| F26 | F30 | 1 |
| F26 | F08 | 1 |
| F26 | M29 | 1 |
| F26 | F36 | 1 |
| F26 | F12 | 1 |
| **Offspring1** | **Offspring2** | **Probability (>0.85)** |
| F27 | F30 | 1 |
| F27 | M29 | 1 |
| F27 | F11 | 1 |
| F27 | F37 | 1 |
| F27 | F12 | 1 |
| F30 | M23 | 1 |
| F30 | F08 | 1 |
| F30 | F11 | 1 |
| M15 | F33 | 1 |
| M15 | F07 | 1 |
| M15 | F10 | 1 |
| M15 | M31 | 1 |
| M05 | M17 | 1 |
| F17 | F32 | 1 |
| F17 | F33 | 1 |
| F17 | F09 | 1 |
| F32 | F33 | 1 |
| F32 | M08 | 1 |
| M23 | F08 | 1 |
| M23 | M29 | 1 |
| M23 | F36 | 1 |
| M23 | F12 | 1 |
| F33 | F07 | 1 |
| F33 | M08 | 1 |
| F33 | F09 | 1 |
| F33 | F10 | 1 |
| F33 | M31 | 1 |
| F07 | F10 | 1 |
| F07 | M31 | 1 |
| M25 | M28 | 1 |
| M08 | F09 | 1 |
| F08 | M29 | 1 |
| F08 | F37 | 1 |
| F08 | F12 | 1 |
| M29 | F11 | 1 |
| F10 | M31 | 1 |
| M28 | F23 | 1 |
| F11 | F12 | 1 |
| M33 | M11 | 1 |
| M11 | M35 | 1 |
| F06 | F17 | 0.973 |
| F06 | M08 | 0.971 |
| M22 | M08 | 0.964 |
| F35 | M08 | 0.951 |
| **Offspring1** | **Offspring2** | **Probability (>0.85)** |
| F17 | F35 | 0.95 |
| M37 | RTA Tohigs M | 0.944 |
| M38 | F19 | 0.894 |
| M03 | F40 | 0.878 |
| M22 | F17 | 0.872 |
| M37 | F40 | 0.866 |
| M37 | M03 | 0.861 |

**Table F. Plural breeding.** Female badgers captured during the spring trapping round of each year that were observed to be lactating. Plural breeders in bold. Plural breeding was confirmed by DNA analysis for F18 and F02 in 2015.

| **Year** | **2010** | **2011** | **2012** | **2013** | **2014** | **2015** | **2016** | **2017** |
| --- | --- | --- | --- | --- | --- | --- | --- | --- |
|  | **F13** | F18 | F06 | F41 | F25 | F44 | F14 | F46 |
|  | **F15** | F03 | **F18** | **F18** | F43 | **F18** | **F18** | F47 |
|  | F05 | F05 | **F02** | **F02** | F02 | **F02** | **F02** | F02 |
|  |  | F06 | F35 | F42 | **F19** | F25 | F43 | F48 |
|  |  |  | F15 |  | **F20** | F45 | **F22** | **F22** |
|  |  |  |  |  |  | F14 | **F21** | **F21** |
|  |  |  |  |  |  | F43 | F44 | F49 |
|  |  |  |  |  |  | **F19** | **F23** | **F23** |
|  |  |  |  |  |  | **F20** | **F24** | **F24** |
|  |  |  |  |  |  | F34 | F20 | F44 |
|  |  |  |  |  |  |  |  | **F50** |
|  |  |  |  |  |  |  |  | **F51** |
|  |  |  |  |  |  |  |  |  |
| **No. Social Groups** | 1 | 0 | 1 | 1 | 1 | 2 | 3 | 3 |
| **Mean** |  |  |  |  |  |  |  | **1.6** |
| Min |  |  |  |  |  |  |  | 0 |
| Max |  |  |  |  |  |  |  | 3 |

**Table G.** **Inferred dispersal from maternal assignments.**

| **Offspring ID** | **Assigned Social Group** | **Assigned Maternity** | **Assigned Paternity** | **Inferred Natal Social Group** | **Comments** |
| --- | --- | --- | --- | --- | --- |
| F01 | The Quarry | F23 | M04 | Ballad | First trapped as an autumn yearling in The Quarry. |
| F04 | The Briars | F23 | NA | Ballad | First trapped in The Vines (beside The Briars) but only once. Subsequently always trapped in The Briars. |
| M03 | Cemetery | NA | NA | Sycamore | First trapped as an autumn yearling in The Cemetery. |
| F07 | The Orchard | F02 | NA | Oak | First trapped as young adult in The Orchard. |
| M08 | The Orchard | UNKWNF1 | M24 | The Orchard | DNA assignment appears to confirm The Orchard as natal group as per early trapping and early GPS records. |

**References**

Kalinowski ST, Taper ML, Marshall TC (2007) Revising how the computer program CERVUS accommodates genotyping error increases success in paternity assignment. Molecular Ecology 16:1099–1106

Wang J, Santure AW (2009) Parentage and sibship inference from multilocus genotype data under polygamy. Genetics 181:1579–1594
